# Quality of life in patients with HBV infection: A systematic review and meta-analysis

**DOI:** 10.1016/j.jhepr.2024.101312

**Published:** 2025-01-08

**Authors:** Michael X. Fu, Gabriel Lambert, Amelia Cook, Gibril Ndow, Yazan Haddadin, Yusuke Shimakawa, Timothy B. Hallett, Heli Harvala, Elisa Sicuri, Maud Lemoine, Shevanthi Nayagam

**Affiliations:** 1 Department of Metabolism, Digestion and Reproduction, Division of Digestive Diseases, Imperial College London, London, UK; 2 Nuffield Department of Medicine, University of Oxford, Oxford, UK; 3 Cicely Saunders Institute for Palliative Care, Policy and Rehabilitation, King's College London, London, UK; 4 Medical Research Council Unit, The Gambia, London School of Hygiene & Tropical Medicine, Banjul, The Gambia; 5 Department of Clinical and Experimental Medicine, University of Sussex, Brighton, UK; 6 Unité d'Épidémiologie des Maladies Émergentes, Institut Pasteur, Paris, France; 7 MRC Centre for Global Infectious Disease Analysis, Department of Infectious Disease Epidemiology, Faculty of Medicine, Imperial College London, London, UK; 8 Microbiology Services, NHS Blood and Transplant, London, UK; 9 Radcliffe Department of Medicine, University of Oxford, Oxford, UK; 10 Division of Infection and Immunity, University College London, London, UK; 11 LSE Health, London School of Economics and Political Science, London, UK; 12 ISGlobal, Barcelona, Spain; 13 Centro de Investigação em Saúde de Manhiça (CISM), Maputo, Mozambique; 14 Facultat de Medicina i Ciències de la Salut, Universitat de Barcelona (UB), Barcelona, Spain

**Keywords:** Health-related quality of life, Health utilities, Global health, Liver cirrhosis, Hepatitis, Health status

## Abstract

**Background & Aims:**

Despite nearly 250 million people worldwide estimated to have chronic HBV infection, health-related quality of life (HRQOL) in HBV-related disease has not been well characterised. Here, we summarise existing data on HBV-related HRQOL and quantify summary utility values by stage of disease.

**Methods:**

Embase, Global Health, PubMed, and Web of Science were searched for articles investigating HBV HRQOL. Meta-analyses for utility scores were pooled by stage of disease and utility instrument; meta-regression was further adjusted for the effect of current health expenditure as a percentage of gross domestic product (CHE/GDP), as a proxy of the importance of healthcare perceived by different countries.

**Results:**

Twenty-two articles from 19 studies, comprising 10,311 patients, were included. Of these studies, 74% were performed in the Western Pacific Region, and 47% used the EuroQoL-5D-3L instrument. HRQOL was found to decrease with advancing stages of HBV-related disease. Meta-regression showed the following predicted mean utility scores for the different stages of chronic HBV infection: non-cirrhotic, 0.842; compensated cirrhosis, 0.820 (*p* = 0.474 compared with non-cirrhotic); decompensated cirrhosis, 0.722 (*p* = 0.001); and hepatocellular carcinoma, 0.749 (*p* = 0.008). The type of tool affected HRQOL and populations with a higher CHE/GDP were associated with higher predicted utility values.

**Conclusions:**

Chronic HBV infection impairs the HRQOL of patients, even when there is no evidence of cirrhosis. HRQOL is particularly impaired in the advanced stages of decompensated cirrhosis and hepatocellular carcinoma. These results have important implications for global hepatitis elimination efforts and are useful for economic analyses. However, further research is needed, particularly in high-burden, low-income settings where data are lacking.

**Impact and implications:**

This study, based on 22 articles and 10,311 patients, provides a comprehensive synthesis of data on the impact of chronic hepatitis B virus (HBV) infection on patients’ health-related quality of life (HRQOL) worldwide. These findings, of how HRQOL is affected in people living with HBV, highlight the importance of patient-centred care and holistic approaches to management, even at the early stages of disease. These results are useful for cost-effectiveness analyses and may help inform decision-making in improving public health policy towards the elimination of viral hepatitis. The study also underscores the need for further data from low-to middle-income settings, and on the effects of treatment on HRQOL.

## Highlights

• Global data show that quality of life is impacted by HBV infection.
• Complications of HBV infection significantly decrease quality of life.
• Country-level inequalities in access to healthcare affect quality of life.
• More attention should be placed on the quality of life of patients with HBV.

## Graphical abstract

**Figure:**
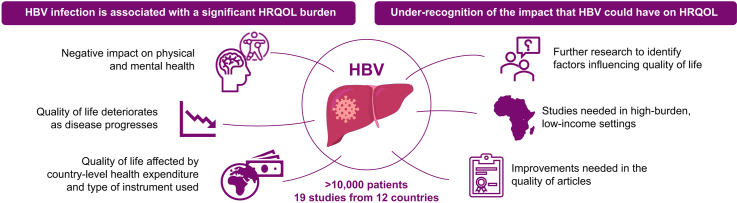

## Introduction

In 2016, the World Health Organization (WHO) adopted the first global targets to eliminate viral hepatitis.^1^ In 2022, 246 million people worldwide were estimated to be chronically infected with HBV, resulting in 1.1 million annual deaths.^2^ Infected individuals can develop complications, including cirrhosis and hepatocellular carcinoma (HCC).^3^ This growing disease burden represents a clinical and economic challenge for healthcare systems, particularly in low- and middle-income countries (LMICs), where the burden is the highest and resources are most constrained.^4^ In addition to the significant morbidity and mortality caused by the disease, HBV infection can also affect patients’ health-related quality of life (HRQOL). Accurate quantification can help better guide public health policies to improve overall health and well-being and target interventions appropriately.

HRQOL refers to the impact of health on a patient’s functioning and well-being and is a multidimensional concept that incorporates physical, mental, and social functions.^5^ Chronic HBV infection (CHB) has both a complex natural history and often a long asymptomatic phase.^6^ However, a comprehensive, holistic evaluation through HRQOL allows for other factors that affect patient well-being to be considered, including HBV-related stigma, fear of transmission to others, and early impact on activities of daily living.^7^^,^^8^ Utility values from certain HRQOL instruments provide a summary score of a patient’s or the general population’s preference and valuation for a specific level of health status, and are commonly scored on an interval scale from 0 (worst imaginable health) to 1 (perfect health).^9^ Health utilities are useful for quantifying the health burden of disease, can be used to calculate quality-adjusted life years (QALYs) which are routinely used in economic analyses.^10^ These have not been well characterised in HBV-related diseases, with existing studies using disparate tools and methods and focussing on different stages of liver disease and different population groups.

In this study, we summarise existing data on HBV-related HRQOL and quantify health utility scores by stage of disease and instrument used through a systematic review and meta-analysis. This will enable a better understanding of factors driving HRQOL in patients living with CHB, be useful for more accurately parameterising cost-effectiveness analyses, and identify key data gaps.

## Materials and methods

### Search strategy and selection criteria

We searched four databases (Embase, Global Health, PubMed, and Web of Science) from their inception until January 9, 2024. The search strategy combined the following terms and their variations; ‘HBV’, ‘quality of life’, and ‘health utilities’ (Appendix S1). We reviewed references from relevant reviews and articles on cost-effectiveness to ensure the comprehensiveness of the search results.

Original articles of any study design, excluding abstracts, which reported original health utility data for patients diagnosed with chronic HBV infection (HBsAg positivity for at least 6 months), were included. For inclusion, articles needed to report a composite utility estimate and a measure of uncertainty where the standard error could be estimated, such as CIs, SD, and sample size. We only included articles available in English. We excluded articles reporting utility values for a mixed cohort that included patients living with CHB but did not report the HBV-specific utilities, as well as articles reporting post-transplantation results. We also excluded articles including patients with multiple aetiologies for their liver disease (such as co-infection with HCV or HIV) because we could not determine which disease was primarily responsible for HRQOL impairments.

Following duplicate removal, two independent reviewers (MXF, GL, or AC) screened titles and abstracts to identify articles meeting the inclusion criteria and reviewed eligible full texts. Disagreements were resolved by consensus. The following data were extracted from each included article using a standardised data extraction template: study setting (country, year, and study design), patient demographics (age, sex, and ethnicity), clinical characteristics (stage of disease and treatment status), and health utility estimates (including measures of uncertainty and utility instrument used). Data were extracted by a single reviewer (MXF or GL) and then verified by a second reviewer (MXF or HH). If HBV utilities were reported in more than one article for the same cohort, data were only extracted from the article with more comprehensive data (*i.e.* more recent timepoint, larger sample size, or inclusion of an assessment of different tools). These are referred to as separate articles from the same study hereafter. The risk of bias assessment was based on the criteria outlined in the National Institute for Health and Care Excellence (NICE) guidance document on systematic reviews of utilities^11^ and a checklist of HRQOL studies from a previous systematic review^12^ (see Appendix S2 for the risk of bias assessment checklist created).

### Data analysis

We categorised composite utility scores into the following mutually exclusive health states based on patients’ liver disease severity: non-cirrhotic (no evidence of cirrhosis), compensated cirrhosis (CC; cirrhosis with no symptoms of decompensation), decompensated cirrhosis (DC; cirrhosis with a history of symptoms, such as jaundice, ascites, encephalopathy, or variceal bleeding), and HCC. Control groups were excluded from the meta-analysis because there were only four studies that included control groups as direct comparators to patients with HBV infection; from these studies, there was significant heterogeneity in the definitions of the control populations, HRQOL tools utilised, and disease stages. Where the disease stage was only provided in aggregated format or was unclear in a particular study, these data were excluded from the meta-analyses. Stage-specific utility values were only included if articles provided both aggregated and stage-specific values. In addition, if there was more than one timepoint of data presented for a cohort of patients, for example, through follow-up or following treatment, only the baseline data were included.

For each article, the WHO region and the 2021 current health expenditure as a percentage of gross domestic product (CHE/GDP) from the WHO Global Health Observatory^12^ were obtained (Appendix S3). CHE/GDP indicates the proportion of public and private spending for healthcare relative to the output of an economy and has previously been suggested to serve as an indicator of the societal importance of the healthcare sector to the overall economy and, crucially, to that population.^13^

If more than one study used the same HRQOL tool to describe the same stage of CHB, results from these studies for the same tool and stage of disease were pooled via meta-analysis. Here, studies were weighted by the inverse squared standard error. DerSimonian–Laird random effects models were used to analyse pooled subgroups with four or more studies. Subgroups with fewer than four studies were deemed insufficient to estimate interstudy heterogeneity and were analysed instead with fixed-effects models.^14^

We then performed a meta-regression to predict mean utility estimates for each stage of disease across all utility instruments and CHE/GDP values. In contrast to the meta-analyses, the inclusion of interactions in the multivariate meta-regression model accounted for multiple utility estimates from the same study cohorts evaluated by different tools. Chronic Liver Disease Questionnaire (CLDQ) utilities were normalised to 0–1 scales in the model to enable comparisons with the other instruments.^15^ The *I*^2^ statistic evaluated statistical heterogeneity, whereas funnel plots and Egger’s regression tests assessed publication bias. Data analysis was conducted using the ‘metafor’ package in R (R Foundation for Statistical Computing, Vienna, Austria)^16^ and Microsoft Excel (Microsoft Corporation, Redmond, WA, USA). PRISMA guidelines were adhered to, and the study protocol was registered in PROSPERO: CRD42021134803. Ethical approval was not required because this was a systematic review and meta-analysis using published aggregated data.

## Results

### Study selection

**Fig. 1:**
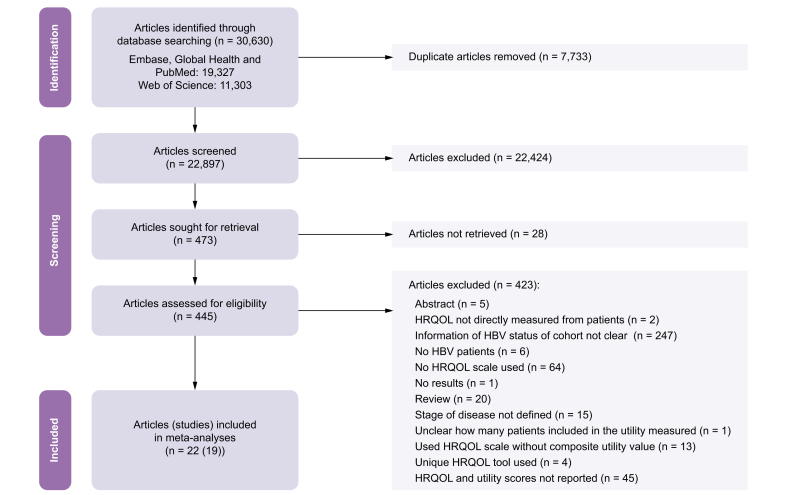
PRISMA flow diagram^67^ outlining identifying, screening, and including articles and unique studies. HRQOL, health-related quality of life; PRISMA, Preferred Reporting Items for Systematic Reviews and Meta-Analyses.

Of 30,630 articles identified, 22 (including 19 unique studies) met the inclusion criteria for this systematic review[17], [18], [19], [20], [21], [22], [23], [24], [25], [26], [27], [28], [29], [30], [31], [32], [33], [34], [35], [36], [37], [38] (Fig. 1). Appendix S4 details the reasons for excluding full texts (including 13 articles where HRQOL scales without a composite utility value were used: Short Form [SF]-36; WHO Quality of Life-abbreviated form (WHOQOL-BREF), and Hepatitis Quality of Life Questionnaire [HQLQ]), and Appendix S5 details the 15 excluded articles in which the stage of HBV disease was unclear. The 19 included studies had 16,451 individual utility measurements from 10,311 unique patients (Table 1). Most studies were conducted in the Western Pacific WHO region (74%), with minimal studies in all other regions and none in the African or Eastern Mediterranean regions. The most utilised utility instruments were the EuroQol-5D-3L (EQ-5D-3L) (47%), visual analogue scale (VAS) (47%), and CLDQ (42%). Of the 13 studies that reported patients’ age, the mean age was 43. Fifteen studies reported sex, of which 67% identified as male. Three studies had longitudinal follow-up data,^17^^,^^31^^,^^32^ three had a general cirrhosis stage (which did not differentiate compensated and decompensated cirrhosis);^19^^,^^25^^,^^30^ these six studies were excluded from meta-analyses and meta-regression. Health Utilities Index 2 (HUI2) and HUI3 tools were excluded from the meta-analyses because of the low number of studies. Only one study provided composite utility values for the HBV quality of life (HBQOL) instrument but without enough information to ascertain the stage of disease; thus, this study and instrument were excluded from analyses (Appendix S4). A summary of the findings from included studies that had control and treatment populations is provided in Appendix S6.

**Table 1:** Characteristics of each of the included studies (n = 19).

Study	Period of study procedures	Study design	Country	WHO region	Identify as male (%)	Age (mean)	Non-white race (%)	Treatment details	Stage(s) of disease	No. of patients	Utility instrument(s)
Ansari *et al.* 2019^17^	2015–2017	Int	India	Southeast Asia	ND	ND	ND	Traditional medicine for 90 days	Non-cirrhotic, *treatment*	30	EQ-5D-3L, VAS
Che *et al.* 2014^18^	2012–2013	Obs	China	Western Pacific	72.0	45.0	ND	ND	Non-cirrhotic CC DC HCC	520 91 198 131	CLDQ, EQ-5D-3L, VAS
Chen *et al.* 2021^19^	2013	Obs	China	Western Pacific	69.5	ND	ND	100% on antiviral	Non-cirrhotic *Cirrhotic*	98 *56*	CLDQ, EQ-5D-3L, VAS
Cortesi *et al.* 2020^20^	2011–2013	Obs	Italy	Europe	ND	ND	ND	ND	Non-cirrhotic	284	EQ-5D-3L, VAS
Dan *et al.* 2008^21^	1997–2005	Obs	USA	Americas	74.5	47.3	ND	Exclude interferon	Non-cirrhotic (3.9% were cirrhotic)	51	SF-6D, *HUI2*
Gupta *et al.* 2020^22^	2014–2015	Obs	India	Southeast Asia	85.3	39.0	ND	ND	*All stages* Non-cirrhotic CC	*150* 75 75	CLDQ
Jia *et al.* 2014^23^	2013	Obs	China	Western Pacific	75.0	43.9	ND	ND	Non-cirrhotic CC DC HCC	319 114 107 105	EQ-5D-3L, EQ-5D-5L, VAS
Kim *et al.* 2012^24^	2007	Int	South Korea	Western Pacific	72.1	43.3	ND	40.8% previous treatment	Non-cirrhotic CC DC	2,286 367 103	CLDQ
Lam *et al.* 2009^25^	2006–2008	Obs	Hong Kong	Western Pacific	73.8	50.4	ND	42.9% previous antiviral	*All stages* Non-cirrhotic *Cirrhotic* HCC	520 258 139 123	SF-6D, CLDQ
Levy *et al.* 2008^27^	Pre-2008	Obs	Mixed	—	ND	ND	ND	ND	Non-cirrhotic CC DC HCC	225 98 49 39	SG
Liu *et al.* 2016^28^	2011–2012	Obs	China	Western Pacific	58.4	38.8	ND	71.7% on antiviral	Non-cirrhotic CC DC	405 53 61	CLDQ
Siew *et al.* 2008^29^	2003–2006	Obs	Singapore	Western Pacific	ND	ND	ND	ND	Non-cirrhotic CC DC HCC	298 66 24 22	EQ-5D-3L, VAS
Sugimori *et al.* 2022^38^	2012	Obs	Japan	Western Pacific	54.5	ND	ND	ND	Non-cirrhotic CC DC	1,021 141 35	EQ-5D-5L
Woo *et al.* 2012^37^	2007–2009	Obs	Canada	Americas	70.5	50.0	ND	47.7% on antiviral	Non-cirrhotic CC DC HCC	294 79 7 23	EQ-5D-3L, VAS, *HUI3*, SG
Wu *et al.* 2021^31^	2013–2015	Int	China	Western Pacific	77.6	48.0	ND	Treatment naïve, then treated with entecavir for 5 years	CC *treatment*	161 *133*	EQ-5D-3L, VAS
Xue *et al.* 2017^32^	Pre-2017	Int	China	Western Pacific	79.4	36.6	100	47.1% previously treated, then treated with antivirals for 48 weeks	Non-cirrhotic, *treatment*	102	VAS
Younossi *et al.* 2018^33^	2015–2017	Int	Mixed	—	67.4	43.5	78.5	Antivirals >12 months ± vestatolimod 11 weeks	Non-cirrhotic	242	SF-6D, CLDQ
Zhang *et al.* 2021^35^	2019–2020	Obs	China	Western Pacific	69.1	42.5	ND	75.0% on antiviral	Non-cirrhotic CC DC HCC	639 125 85 222	SF-6D
Zhuang *et al.* 2014^36^	2010	Obs	China	Western Pacific	66.3	35.8	100	38.9% on antiviral	*All stages* Non-cirrhotic CC DC	*460* 323 54, 83	SF-6D, CLDQ

Italicised stages of disease or tools denote that these were not included in the analyses. CC, compensated cirrhosis; CLDQ, Chronic Liver Disease Questionnaire; DC, decompensated cirrhosis; EQ-5D-3L, EuroQol-5D 3 levels; EQ-5D-5L, EuroQol-5D 5 levels; HCC, hepatocellular carcinoma; Int, interventional study; ND, no data available; obs, observational study; SF-6D, Short Form-6D; SG, Standard Gamble; VAS, visual analogue scale.

### Risk of bias assessment

Appendix S7 details the risk of bias assessment for individual studies. Most studies did not adequately describe their study design or mention the exclusion of other aetiologies of liver disease. Most studies also failed to assess the stage of CHB and did not provide adequate diagnostic criteria. The disease stages in studies that evaluated this were generally not well defined. The timing of HRQOL assessment and response rate was missing from most studies, and reporting of the presence or absence of missing data was also lacking.

### Meta-analysis: health utility by disease stage and utility instrument

The most extensive data were available for the non-cirrhotic stage of HBV (Table 2). Compared with the non-cirrhotic stage, utilities were lower for CC in all utility instruments except VAS. Utilities were also lower for DC than CC across all instruments and most pronounced in the Standard Gamble (SG) and VAS instruments. HCC utility scores were lower than for DC when pooled using SF-6D, but HCC scores were higher than for DC for the other instruments; HCC values for all instruments were lower than for CC. Forest plots and *I*^2^ values are presented in Appendix S8.

**Table 2:** Meta-analysis results by stage of disease and utility instrument.

Subgroup	Utility instrument					
	CLDQ (1.00–7.00)	EQ-5D-3L (0.000–1.000)	EQ-5D-5L (0.000–1.000)	SF-6D (0.000–1.000)	SG (0.000–1.000)	VAS (0.0–100.0)
Non-cirrhotic	5.39 ± 0.10 8 studies 4,207 patients	0.834 ± 0.050 7 studies 1,843 patients	0.817 ± 0.002 2 studies 1,340 patients	0.753 ± 0.019 5 studies 1,513 patients	0.813 ± 0.006 2 studies 519 patients	71.5 ± 3.0 8 studies 1,945 patients
Compensated cirrhosis	4.76 ± 0.25 5 studies 640 patients	0.816 ± 0.043 5 studies 511 patients	0.774 ± 0.005 2 studies 255 patients	0.698 ± 0.010 2 studies 179 patients	0.743 ± 0.011 2 studies 177 patients	73.7 ± 4.2 5 studies 511 patients
Decompensated cirrhosis	4.62 ± 0.13 4 studies 445 patients	0.712 ± 0.054 4 studies 336 patients	0.665 ± 0.010 2 studies 142 patients	0.673 ± 0.010 2 studies 168 patients	0.361 ± 0.013 2 studies 56 patients	59.0 ± 5.1 4 studies 336 patients
Hepatocellular carcinoma	4.67 ± 0.07 2 studies 254 patients	0.731 ± 0.050 4 studies 281 patients	0.699 ± 0.119 1 study 105 patients	0.662 ± 0.008 2 studies 345 patients	0.433 ± 0.012 2 studies 62 patients	66.5 ± 7.1 4 studies 281 patients

Data are presented as mean ± SE, with the number of studies and the total number of patients for each subgroup indicated. Scales for each utility instrument are shown as (worse health state utility to best health state utility). CLDQ, Chronic Liver Disease Questionnaire; EQ-5D-3L, EuroQol-5D 3 levels; EQ-5D-5L, EuroQol-5D 5 levels; SF-6D, Short Form-6D; SG, Standard Gamble; VAS, visual analogue scale.

### Meta-regression

**Fig. 2:**
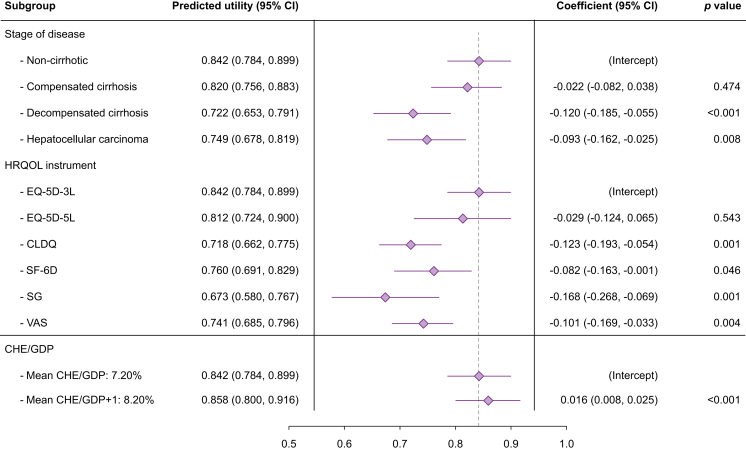
Results of meta-regression showing the predicted HRQOL utility estimates (mean, 95% CIs) for each subgroup in the meta-regression, along with the coefficients and *p* values for each subgroup. The utility for the mean CHE/GDP and 1% CHE/GDP increase from the mean are shown. *P* values are derived from the restricted maximum likelihood meta-regression model, where *p* <0.05 indicates statistical significance. CLDQ, Chronic Liver Disease Questionnaire; EQ-5D-3L, EuroQol-5D 3 levels; EQ-5D-5L, EuroQol-5D 5 levels; HRQOL, health-related quality of life; SF-6D, Short Form-6D; SG, Standard Gamble; VAS, visual analogue scale.

The meta-regression model (Fig. 2), performed on 86 subgroups of patients from the 19 included studies,[17], [18], [19], [20], [21], [22], [23], [24], [25]^,^[27], [28], [29]^,^[31], [32], [33]^,^[35], [36], [37], [38] had an intercept of 0.842 ± 0.029, representing the pooled HRQOL utility score for the most common stage of HBV disease (non-cirrhotic), most common utility instrument (EQ-5D-3L), and mean CHE/GDP of 7.20%. Utility estimates were significantly lower for the DC (0.722 ± 0.035; *p* <0.001) and HCC (0.749 ± 0.036; *p* = 0.008) stages than for the non-cirrhotic stage. EQ-5D-3L brought about the highest utility estimates, followed by EQ-5D-5L (0.812 ± 0.048; *p =* 0.543) and SF-6D (0.760 ± 0.041; *p* = 0.046). CLDQ (0.718 ± 0.036; *p* = 0.001) and VAS (0.741 ± 0.035; *p* = 0.004) had significantly lower estimates compared with EQ-5D-3L, whereas SG produced the lowest coefficient (0.673 ± 0.051; *p* = 0.001). Moreover, with every 1% increase in CHE/GDP, the utility was predicted to increase significantly by 0.016 (*p* <0.001), and countries with lower CHE/GDP were expected to have lower utility scores (Table 3).

**Table 3:** Predicted HRQOL utility estimates generated from the meta-regression model by stage of disease and CHE/GDP, standardised to each utility instrument.

Utility instrument	Stage of disease	CHE/GDP 3.60% (95% CI)	CHE/GDP 7.20% (95% CI)	CHE/GDP 14.39% (95% CI)
EQ-5D-3L	Non-cirrhotic	0.783 (0.718–0.848)	0.842 (0.784–0.899)	0.959 (0.873–1.046)
	Compensated cirrhosis	0.761 (0.693–0.829)	0.820 (0.756–0.883)	0.937 (0.844–1.031)
	Decompensated cirrhosis	0.663 (0.590–0.736)	0.722 (0.653–0.791)	0.840 (0.741–0.938)
	Hepatocellular carcinoma	0.690 (0.615–0.764)	0.749 (0.678–0.819)	0.866 (0.766–0.966)
EQ-5D-5L	Non-cirrhotic	0.753 (0.657–0.850)	0.812 (0.724–0.900)	0.930 (0.826–1.034)
	Compensated cirrhosis	0.731 (0.636–0.827)	0.790 (0.701–0.880)	0.908 (0.800–1.016)
	Decompensated cirrhosis	0.634 (0.536–0.731)	0.692 (0.601–0.784)	0.810 (0.700–0.920)
	Hepatocellular carcinoma	0.660 (0.558–0.762)	0.719 (0.622–0.816)	0.837 (0.722–0.952)
CLDQ	Non-cirrhotic	4.96 (4.59–5.33)	5.31 (4.97–5.65)	6.02 (5.48–6.55)
	Compensated cirrhosis	4.83 (4.43–5.22)	5.18 (4.79–5.56)	5.88 (5.30–6.47)
	Decompensated cirrhosis	4.24 (3.82–4.66)	4.59 (4.18–5.00)	5.30 (4.69–5.90)
	Hepatocellular carcinoma	4.40 (3.94–4.86)	4.75 (4.30–5.20)	5.46 (4.82–6.10)
SF-6D	Non-cirrhotic	0.701 (0.624–0.778)	0.760 (0.691–0.829)	0.877 (0.785–0.970)
	Compensated cirrhosis	0.679 (0.596–0.762)	0.738 (0.660–0.816)	0.855 (0.753–0.958)
	Decompensated cirrhosis	0.581 (0.496–0.666)	0.640 (0.560–0.720)	0.758 (0.653–0.862)
	Hepatocellular carcinoma	0.608 (0.521–0.694)	0.666 (0.584–0.749)	0.784 (0.677–0.891)
SG	Non-cirrhotic	0.615 (0.504–0.725)	0.673 (0.580–0.767)	0.791 (0.702–0.880)
	Compensated cirrhosis	0.593 (0.483–0.702)	0.651 (0.558–0.745)	0.769 (0.677–0.861)
	Decompensated cirrhosis	0.495 (0.384–0.606)	0.554 (0.457–0.650)	0.671 (0.576–0.766)
	Hepatocellular carcinoma	0.521 (0.411–0.632)	0.580 (0.484–0.676)	0.698 (0.602–0.793)
VAS	Non-cirrhotic	68.2 (61.9–74.5)	74.1 (68.5–79.6)	85.8 (77.3–94.4)
	Compensated cirrhosis	66.0 (59.2–72.7)	71.9 (65.6–78.2)	83.6 (74.3–93.0)
	Decompensated cirrhosis	56.2 (49.0–63.5)	62.1 (55.2–68.9)	73.9 (64.1–83.6)
	Hepatocellular carcinoma	58.9 (51.5–66.2)	64.7 (57.7–71.8)	76.5 (66.5–86.5)

The mean CHE/GDP (7.40%) is shown, as well as half of the mean CHE/GDP and double the mean CHE/GDP. CHE/GDP, current health expenditure as a percentage of gross domestic product; CLDQ = Chronic Liver Disease Questionnaire; EQ-5D-3L = EuroQol-5D 3 levels; HRQOL, health-related quality of life; SF-6D = Short Form-6D; SG = Standard Gamble; VAS = Visual Analogue Scale.

### Heterogeneity and funnel plots

The random effects meta-analysis by disease stage and utility tool had *I*^2^ values ranging from 82.1% to 99.7% (see Appendix S8 for all *I*^2^ values). In addition, the meta-regression model had an *I*^2^ index of 99.1%. These values indicate that a substantial proportion of the observed variances was due to variance in actual effect sizes rather than to any sampling variance, where other unexplained factors resulted in between-study differences. Funnel plots and Egger’s regression test for funnel plot asymmetry did not reveal any substantial asymmetry for all stages of disease except non-cirrhotic and DC (Appendix S9).

## Discussion

To the best of our knowledge, this study is the first to comprehensively synthesise data on HRQOL in people living with CHB and quantify health utility estimates by stage of disease and utility instrument used. Our results confirm that HRQOL is affected in CHB individuals but is most pronounced at the end stages of liver disease (ESLD) when decompensated cirrhosis and HCC have developed. We also provide a pooled estimate of health utilities by stage of CHB infection, HRQOL tool, and a proxy for economic status that could be used in economic analyses, particularly in countries with no empirical data on HRQOL in HBV. These results enable an improved understanding of the burden of HBV disease and may inform decision-making in improving public health policies toward the elimination of viral hepatitis.

The observed trend in the decrease of HRQOL utility values with the severity of liver disease is likely to result from multiple factors affecting different domains, including symptom burden, psychological impact, and stigma.^39^ Symptoms of decompensated cirrhosis can range from ascites to upper gastrointestinal bleeding requiring recurrent hospital admissions, which contrasts with the non-cirrhotic stages, where patients are largely asymptomatic.^39^^,^^40^ In addition to physical symptoms, ESLD can also have a significant impact on anxiety because the ability of patients to perform their usual activities of daily living and employment opportunities are often affected.^8^ Combined with the potential awareness that ESLD is usually associated with a limited estimated life expectancy of 2 years,^41^ it is understandable that health utilities deteriorate in later stages. However, there might be a selection bias because patients with HBV-related advanced liver disease may be under-represented in such studies. Severe symptoms or encephalopathy, for example, could affect patients' ability to consent to participate in, or complete, HRQOL assessments that might be time-consuming and mostly performed in the outpatient setting. This could mean that the real-life utilities of patients with CHB in the more advanced stages are even lower than found in this study.

Our predicted EQ-5D-3L score for the non-cirrhotic stage is less than the utility value for the population norm in China (0.842 *vs.* 0.966, respectively),^42^ where most of the studies included in this review were conducted. Our predicted EQ-5D-3L score for patients with non-cirrhotic HBV was also lower than that of non-institutionalised residents with no health condition in England (0.949)^43^ and the USA (0.952).^44^ This finding is echoed by the few included studies that had control groups (Appendix S6). The lower HRQOL for non-cirrhotic patients compared with the general population could be explained by the nonspecific symptoms associated with HBV, such as psychological symptoms^45^ or stigma, which can create access barriers to education and employment.^46^ Increasing awareness among healthcare workers that patients with clinically asymptomatic HBV might perceive certain aspects of their lives to be adversely affected by HBV infection is crucial for managing patient experience and encouraging adherence to follow-up.^47^ Our findings support recent calls to create an enabling environment to address stigma and discrimination for individuals following an HBV diagnosis.^3^

Given that an estimated 246 million people are living with HBV globally, it is striking that there were only 19 studies quantifying health utilities in HBV (and another 14 if including studies that evaluated HBV but did not disaggregate or define stages adequately, with another 13 articles that used HRQOL scales without a composite utility value) and no studies from the African region, where the burden of HBV is very high. This is in sharp contrast with other chronic diseases. For example, in HIV, a systematic review found 700 studies performed from 2010 to 2021 using over 65 different HRQOL instruments, across various settings and subpopulations,^48^ and a systematic review of HCV found 51 studies.^14^ The reason for this under-representation is likely to be multifactorial. First, there may be under-recognition of the impact that HBV can have on HRQOL, because it is often considered a ‘silent’ disease. Second, in the case of HCV, the higher number of studies may have been driven by the upsurge in recent clinical trials for directly acting antivirals that included HRQOL as an outcome measure. Incorporating patient-reported outcomes in HBV and HDV trials for novel therapies was recommended in the recent joint American Association for the Study of Live Disease–European Associated for the Study of the Liver ‘Endpoints Meeting’ and should improve this knowledge gap.^49^ The lack of studies could also reflect that research and funding for HBV are not commensurate with its disease burden.^50^ In the field of HIV, where patient-centred care is paramount, it has been proposed that a good HRQOL should be added as a ‘4th pillar’ of elimination.^51^

The predicted pooled utility estimates from our meta-regression model, adjusted to each HRQOL instrument and CHE/GDP values, can enhance the quality and applicability of cost–utility analyses and thereby be used to inform decision-making. Although it is preferable to use empirically derived estimates for each specific population under consideration, our review has demonstrated that these are not currently available, and we recognise that it is costly and not always practical to replicate studies in every setting. However, this should not hinder efforts to perform health technology assessments and advance policy decisions. In the UK, for example, NICE used health utilities from persons living with HBV in Canada to inform their economic analyses of HBV treatment.^52^ Another option, where country-specific health utilities are lacking, is to draw on a pool of studies, whereby the model reduces the likelihood of having inconsistent utilities from varying studies and utility instruments.^11^ Our study provides such values that can be used as a proxy (Table 3).

Around half of the studies included in this review were performed in China/Hong Kong, where the burden of HBV disease is high, with ∼84 million individuals chronically infected.^53^ However, HRQOL tools are underutilised in LMICs despite high HBV prevalence. Instead, disability-adjusted life years (DALYs) are more often used to quantify disease burden in LMICs. DALYs measure the societal disease burden, combining mortality and morbidity, whereas QALYs measure patients’ individual-level utility and preferences.^54^ Factors, such as the limited availability of utility instruments in local languages, mean that QALYs are seldom used in LMICs. However, EQ-5D-5L and other HRQOL instruments are increasingly used in African settings.^55^ More research is necessary to examine HRQOL in LMICs, especially in African settings where the HBV disease burden is highest,^4^ because no data in this WHO region were available at the time of analyses.

Patient-derived utility scales are needed to calculate QALYs in economic evaluations. Quantification of how interventions affect DALYs, QALYs, and overall productivity helps guide health policy. Direct HRQOL tools, SG and VAS, were found to elicit lower utility values compared with indirect tools, the latter of which incorporates societal preferences. These differences suggest that patients with HBV have worse HRQOL utilities compared with societal predictions for the same health states. As seen in HCV-related liver disease,^56^ we also found that the disease-specific CLDQ instrument yielded lower utilities compared with generic instruments. This difference could indicate that generic tools miss other clinically important liver-specific aspects, such as worry, which has previously been found to have a negative impact on HRQOL.^57^^,^^58^ The domain of worry in CLDQ includes specific worries about the impact and development of liver disease, its symptoms, prognosis, and availability of a liver transplant.^59^ Given the paucity of eligible studies, HBV-specific tools, such as the HBQOL^60^ and the CLDQ-HBV,^61^ were not included in the meta-analyses, but exist as further disease-targeted HRQOL instruments to consider.

CHE/GDP is an important public health indicator of country-level healthcare expenditure and is high in developed countries.^62^ When we evaluated the range of CHE/GDP values from 3.28% to 17.36% in included studies, our finding from the meta-regression model that a 1% rise in CHE/GDP significantly increased predicted HRQOL utility (*p* <0.001) suggests that better availability of healthcare resources increases patients’ and societal valuations of health. Our predicted utility of 0.959 for the non-cirrhotic stage, where CHE/GDP is 14.39%, is comparable to the population norm for England (0.949)^43^ and the USA (0.952),^44^ where CHE/GDP is 12.36% and 17.36%, respectively. Although these comparisons do not confirm a causal relationship between HBV infection and HRQOL, they suggest that patients with HBV infection in countries with more effective healthcare systems and access to treatment have better HRQOL. This could also partially explain the striking result where patients with decompensated cirrhosis in countries with high CHE/GDP had better predicted HRQOL compared with non-cirrhotic patients in low CHE/GDP countries. Well-developed countries with increased healthcare funding might have more specialised screening programs for HBV-related complications, such as HCC, where detection of early-stage and, often asymptomatic, HCC is improved.^63^ Furthermore, countries with better-funded healthcare systems could also have less HBV-associated stigma.^64^

Although antiviral therapy is known to slow disease progression effectively,^65^ the long-term effects of HBV treatment regimens on HRQOL are less clear. In our meta-analysis, there were insufficient (five) studies to formally quantify the impact of HBV treatment on HRQOL. Overall, these studies found that the HBV treatments assessed significantly increased HRQOL utility values across different tools and stages of HBV disease (Appendix S6). One of these studies found that 5 years of entecavir treatment significantly enhanced HRQOL in patients with CC and with an improvement in mental health that was likely brought on by the physiological improvements from antiviral therapy.^31^ Similarly, another study found that administering oral antivirals significantly increased CLDQ utility scores, especially in the fatigue and worry domains.^33^

Significant interstudy heterogeneity in quality, methodology, use of utility tools, and how stages of infection were defined limited the synthesis of data. Future empirical studies should be more explicit in defining the stage of HBV-related disease, because this appears to be the most influential factor in determining HRQOL. Further, aggregating HRQOL across disease stages could obscure important variations. There might be other factors, such as patient knowledge, availability of healthcare, and cultural factors, which are common across all stages of liver disease and might have been missed by our meta-analysis. Future research should also clarify treatment specificities since varying regimens, such as interferon and oral antivirals, differ in side-effect profiles.^66^

In conclusion, this systematic review suggests that HRQOL and health utility values are impacted by HBV infection, even in non-cirrhotic patients, and worsen with advancing sequelae of HBV-related liver disease (notably decompensated cirrhosis and HCC). The severity of HBV disease does not solely influence HRQOL, and country-level factors, including access to healthcare, could also be negative determinants of HRQOL. Future studies are needed in high-burden LMICs, including in Africa, where data are currently lacking. Our findings are also important for healthcare professionals and highlight that they should consider patients’ HRQOL in clinical management approaches.

## Abbreviations

CC, compensated cirrhosis; CHB, chronic HBV infection; CHE/GDP, current health expenditure as a percentage of gross domestic product; CLDQ, chronic liver disease questionnaire; DALY, disability-adjusted life year; DC, decompensated cirrhosis; EQ-5D, EuroQol-5D; ESLD, end stages of liver disease; HBQOL, HBV quality of life; HCC, hepatocellular carcinoma; HQLQ, hepatitis quality of life questionnaire; HRQOL, health-related quality of life; HUI, Health Utilities Index; LMIC, low- or middle-income country; NICE, National Institute for Health and Care Excellence; PRISMA, Preferred Reporting Items for Systematic reviews and Meta-Analyses; QALY, quality-adjusted life year; SF-36, Short Form-36; SG, Standard Gamble; VAS, visual analogue scale; WHO, World Health Organization; WHOQOL-BREF WHO Quality of Life-abbreviated form.

## Financial support

The authors did not receive any financial support to produce this manuscript.

## Authors’ contributions

Study concept and design: SN, GL, AC. Data screening and extraction: GL, AC, MXF, HH. Data analysis: MXF. Supervision: SN, ES. Writing of the first draft of the manuscript: MXF, SN, GL. All authors reviewed and approved the manuscript.

## Data availability statement

The data used to support the findings of this study, if not found in the manuscript and its supporting information files, are available from the corresponding author upon request.

## Conflicts of interest

The authors declare no conflicts of interest that pertain to this work.

Please refer to the accompanying ICMJE disclosure forms for further details.
