# Supplementary material for: Quality of life in patients with HBV infection: A systematic review and meta-analysis
Source: JHEP Rep. 2025 Jan 8;7(4):101312. doi: 10.1016/j.jhepr.2024.101312 (PMC11919624; doi:10.1016/j.jhepr.2024.101312)
Supplement: Multimedia component 1 [file mmc1.pdf]

Quality of life in patients with HBV infection: A systematic review and meta-analysis

Michael X Fu, Gabriel Lambert, Amelia Cook, Gibril Ndow, Yazan Haddadin, Yusuke Shimakawa, Timothy B Hallett, Heli Harvala, Elisa Sicuri, Maud Lemoine, Shevanthi Nayagam

Table of contents

Appendix 1……………………..…………………………………………………….……….... 2

Appendix 2……………………………..………………………………………..……...… 3

Appendix 3…………………………………………………………………….…..…………4

Appendix 4…………………………………………………………………………………...6

Appendix 5……………………………………………………………………………………43

Appendix 6……………………………………………………………………………………44

Appendix 7……………………………………………………………………………………46

Appendix 8……………………………………………………………………………………50

Appendix 9……………………………………………………………………………………62

Supplementary references…………………………………………………………………..67

Appendix 1: Search strategies

**Databases searched**: Embase, Embase Classic, Global Health, PubMed, Web of Science	
**Date of search:** 20-21 March 2019, updated 5 September 2022 and 9 January 2024	
**#**	**Search**
1	Viral hepatitis
2	Hepatitis B
3	HBV
4	Liver failure
5	Hepatic failure
6	Liver insufficiency
7	Hepatic insufficiency
8	Chronic hepatitis disease
9	End stage liver disease
10	#1 OR #2 OR #3 OR #4 OR #5 OR #6 OR #7 OR #8 OR #9
11	Quality of life
12	Health related quality of life
13	QoL
14	HRQL
15	Patient reported outcome
16	Health utility
17	Activities of daily living
18	ADLs
19	Quality adjusted life year
20	QALY
21	Disability-adjusted life year
22	DALY
23	Short form 36
24	SF 36
25	EuroQol Group 5 dimensions
26	EQ 5D
27	Functional Assessment of Chronic Illness Therapy Fatigue
28	Fatigue Severity Scale
29	FACIT F
30	FSS
31	Chronic Liver Disease Questionnaire
32	Work Productivity and Activity Instrument
33	WPAI
34	Gamble Utility
35	Liver Disease Quality of Life Instrument
36	LDQOL
37	Sickness Impact Profile
38	SIP
39	#11 OR #12 OR #13 OR #14 OR #15 OR #16 OR #17 OR #18 OR #19 OR #20 OR #21 OR #22 OR #23 OR #24 OR #25 OR #26 OR #27 OR #28 OR #29 OR #30 OR #31 OR #32 OR #33 OR #34 OR #35 OR #36 OR #37 OR #38
40	#10 AND #39

Appendix 2: Quality assessment checklist, gathered from National Institute for Health and Care Excellence (NICE) Decision Support Unit guidance document on systematic reviews of utilities[1] and a checklist of HRQOL studies from a previous systematic review[2].

Study Design
**Study objectives**: were the objectives of the study clearly stated? HRQOL primary or secondary outcome?
**Study design**: was the design of the study clearly described?
**Respondent selection and recruitment**: was the sampling method for participant recruitment adequately described?
**Sample size**: was the sample size appropriately justified?
**Participant characteristics**: were characteristics clearly described (demographics and clinical variables)?
**Inclusion/exclusion criteria**: are inclusion/exclusion criteria clearly described? Specifically, efforts to exclude other aetiologies of liver disease.
**Diagnostic criteria**: are there adequate diagnostic criteria for HBV (serology markers)?
**Stage assessment**: is stage of HBV disease assessed?
HRQOL Instrument and Results
**Instrument justification**: was the choice of HRQOL instrument justified?
**Instrument validation**: was a validated tool used to assess HRQOL in the appropriate population?
**Timing of assessments**: is timing reported and consistent?
**Response rates**: are response rates recorded and if so, are the rates likely to be a threat to validity?
**Missing data**: are the levels of missing data reported? Could this threaten the validity of the results?
**Statistical analysis**: were statistical methods described?
Interpretation
**Study findings**: were the key findings of the study clearly stated?
**Study limitations**: were limitations of the study clearly described?

Appendix 3: Country-level current health expenditure (CHE) as a percentage of gross domestic product (GDP) (%)

CHE as a % of GDP for each country as of 2021 was derived from the WHO Global Health Repository[3].

Country	CHE as % of GDP (%)
Canada	12.33
China/Hong Kong	5.38
India	3.28
Italy	9.38
Japan	10.82
Singapore	5.57
Republic of Korea	9.33
USA	17.36

For the two studies conducted in multiple countries, an estimate of the CHE as a % of the GDP of the whole cohort was calculated based on the proportions of HBV-infected patients in each country:

Levy 2008[4]*:*

Country	CHE as % of GDP (%)	Number of patients
USA	17.36	56
Canada	12.33	100
United Kingdom	12.36	93
Spain	10.74	85
Hong Kong/China	5.38	200
Overall	**10.01**	534

Younossi 2018[5]:

**Country**	**CHE as % of GDP (%)**	**Number of patients**
Canada	12.33	30
Italy	9.38	59
Republic of Korea	9.33	56
USA	17.36	49
Overall	**11.84**	194

Appendix 4: Citations of excluded full texts, with reasons for exclusion

#	Citation	Reason for exclusion
1	Abdo AA. Health-related quality of life of Saudi hepatitis B and C patients. Ann Saudi Med. 2012;32(4):397-403. doi:10.5144/0256-4947.2012.397	Used HRQOL scale without composite utility value (SF-36)
2	Abedi G, Azadeh A, Farideh R. Assessment of quality of life in hepatitis B patients compared with healthy people. Life Sci J. 2012;9(4):5339-5343.	Utility scores not reported
3	Abedi G, Rostami F, Nadi A. Analyzing the Dimensions of the Quality of Life in Hepatitis B Patientsusing Confirmatory Factor Analysis. Glob J Health Sci. 2015;7(7 Spec No):22-31. Published 2015 Mar 26. doi:10.5539/gjhs.v7n7p22	Utility scores not reported
4	Åberg F, Mäklin S, Räsänen P, et al. Cost of a quality-adjusted life year in liver transplantation: the influence of the indication and the model for end-stage liver disease score. Liver Transpl. 2011;17(11):1333-1343. doi:10.1002/lt.22388	Information of HBV status of cohort not clear
5	Adekanle O, Ndububa DA, Orji EO, Ijarotimi O. Assessment of the sexual functions of males with chronic liver disease in South West Nigeria. Ann Afr Med. 2014;13(2):81-86. doi:10.4103/1596-3519.129884	No HRQOL scale used
6	Adekanle O, Sunmonu TA, Komolafe MA, Ndububa DA. Cognitive functions in patients with liver cirrhosis: assessment using community screening interview for dementia. Ann Afr Med. 2012;11(4):222-229. doi:10.4103/1596-3519.102853	No HRQOL scale used
7	Afendy A, Kallman JB, Stepanova M, et al. Predictors of health-related quality of life in patients with chronic liver disease. Aliment Pharmacol Ther. 2009;30(5):469-476. doi:10.1111/j.1365-2036.2009.04061.x	Information of HBV status of cohort not clear
8	Aggarwal R, Ghoshal UC, Naik SR. Treatment of chronic hepatitis B with interferon-alpha: cost-effectiveness in developing countries. Natl Med J India. 2002;15(6):320-327.	No HRQOL scale used
9	Aguiar I, Lins-Kusterer L, Lins LS, Paraná R, Bastos J, Carvalho FM. Quality of life, work ability and oral health among patients with chronic liver diseases. Med Oral Patol Oral Cir Bucal. 2019;24(3):e392-e397. Published 2019 May 1. doi:10.4317/medoral.22918	Information of HBV status of cohort not clear
10	Ahluwalia V, Wade JB, Thacker L, et al. Differential impact of hyponatremia and hepatic encephalopathy on health-related quality of life and brain metabolite abnormalities in cirrhosis. J Hepatol. 2013;59(3):467-473. doi:10.1016/j.jhep.2013.04.023	Information of HBV status of cohort not clear
11	Ahluwalia V, Wade JB, White MB, et al. Brain Integrity Changes Underlying Cognitive and Functional Recovery Postliver Transplant Continue to Evolve Over 1 Year. Transplantation. 2018;102(3):461-470. doi:10.1097/TP.0000000000001991	Information of HBV status of cohort not clear

12	Ahluwalia V, Wade JB, White MB, et al. Liver transplantation significantly improves global functioning and cerebral processing. Liver Transpl. 2016;22(10):1379-1390. doi:10.1002/lt.24498	Information of HBV status of cohort not clear
13	Ahmed J, Butt NF, Rathore R, et al. Prevalence of depression in patients of chronic hepatitis B and C presenting to Mayo Hospital Lahore. Pakistan Journal of Medical and Health Sciences. 2014;11(2):690.	No HRQOL scale used
14	Alam RR, El-Gilany AE. Quality of life of elderly with chronic liver diseases. Hepatology International. 2019;13(S1):S9. doi:10.1007/s12072-019-09936-5	Information of HBV status of cohort not clear
15	Alay H, Özden K, Erol S, Çelik N, Parlak E, Parlak M. Assessment of Quality of Life of Patients with Chronic Hepatitis B and C Treated with Pegylated Interferon-alpha. Viral Hepat J. 2018;24:25-42. doi:10.4274/vhd.2017.0016	Utility scores not reported
16	AlHuthail YR. Comparison of the prevalence of psychiatric co-morbidities in hepatitis C patients and hepatitis B patients in Saudi Arabia. Saudi J Gastroenterol. 2013;19(4):165-171. doi:10.4103/1319-3767.114514	Utility scores not reported
17	Alian S, Masoudzadeh A, Khoddad T, Dadashian A, Ali Mohammadpour R. Depression in hepatitis B and C, and its correlation with hepatitis drugs consumption (interfron/lamivodin/ribaverin). Iran J Psychiatry Behav Sci. 2013;7(1):24-29.	No HRQOL scale used
18	Altindag A, Cadirci D, Sirmatel F. Depression and health related quality of life in non-cirrhotic chronic hepatitis B patients and hepatitis B carriers. Neurosciences (Riyadh). 2009;14(1):56-59.	Utility scores not reported
19	AlZoubi AM, Khalifeh F. The effectiveness of stem cell therapies on health-related quality of life and life expectancy in comparison with conventional supportive medical treatment in patients suffering from end-stage liver disease. Stem Cell Res Ther. 2013;4(1):16. Published 2013 Feb 25. doi:10.1186/scrt164	No HRQOL scale used
20	Amarasena S, Skoien R, Bernardes C, et al. Health-related quality of life in patients with cirrhosis. 2019;34(S2):77. doi:10.1111/jhg.14799	Information of HBV status of cohort not clear
21	Anil Kumar BN, Mattoo SK. Organ transplant & the psychiatrist: An overview. Indian J Med Res. 2015;141(4):408-416. doi:10.4103/0971-5916.159268	Review
22	Annema C, Drent G, Roodbol PF, et al. Trajectories of Anxiety and Depression After Liver Transplantation as Related to Outcomes During 2-Year Follow-Up: A Prospective Cohort Study. Psychosom Med. 2018;80(2):174-183. doi:10.1097/PSY.0000000000000539	Information of HBV status of cohort not clear
23	Arguedas MR, DeLawrence TG, McGuire BM. Influence of hepatic encephalopathy on health-related quality of life in patients with cirrhosis. Dig Dis Sci. 2003;48(8):1622-1626. doi:10.1023/a:1024784327783	Information of HBV status of cohort not clear

24	Arvand J, Shafiabadi A, Falsafinejad MR, Naderi N. Depression in patients with chronic hepatitis B: an experience on individual solution- focused therapy. Gastroenterol Hepatol Bed Bench. 2012;5(3):166-168.	No HRQOL scale used
25	Asadi Noghabi AA, Zandi M, Mehran A, Alavian SM, Dehkordi AH. The effect of education on quality of life in patients under interferon therapy. Hepat Mon. 2010;10(3):218-222.	Information of HBV status of cohort not clear
26	Ashrafi M, Modabbernia A, Dalir M, et al. Predictors of mental and physical health in non-cirrhotic patients with viral hepatitis: a case control study. J Psychosom Res. 2012;73(3):218-224. doi:10.1016/j.jpsychores.2012.06.006	Information of HBV status of cohort not clear
27	Atiq M, Gill ML, Khokhar N. Quality of life assessment in Pakistani patients with chronic liver disease. J Pak Med Assoc. 2004;54(3):113-115.	Information of HBV status of cohort not clear
28	Baghernezhad M, Mani A, Ayoobzadehshirazi A, et al. Psychologic Evaluation in Liver Transplantation: Assessment of Psychologic Profile of End-Stage Liver Disease Patients Before and After Transplant. Exp Clin Transplant. 2020;18(3):339-344. doi:10.6002/ect.2017.0135	No HRQOL scale used
29	Bahardoust, M, Mokhtare M, Agah S, et al. Tehran University Medical Journal. 2019;77(3):186-192	Abstract
30	Bajaj JS, Ahluwalia V, Thacker LR, et al. Brain Training with Video Games in Covert Hepatic Encephalopathy. Am J Gastroenterol. 2017;112(2):316-324. doi:10.1038/ajg.2016.544	Information of HBV status of cohort not clear
31	Bajaj JS, Cordoba J, Mullen KD, et al. Review article: the design of clinical trials in hepatic encephalopathy--an International Society for Hepatic Encephalopathy and Nitrogen Metabolism (ISHEN) consensus statement. Aliment Pharmacol Ther. 2011;33(7):739-747. doi:10.1111/j.1365-2036.2011.04590.x	Review
32	Bajaj JS, Fagan A, Sikaroodi M, et al. Liver transplant modulates gut microbial dysbiosis and cognitive function in cirrhosis. Liver Transpl. 2017;23(7):907-914. doi:10.1002/lt.24754	Information of HBV status of cohort not clear
33	Bajaj JS, Heuman DM, Wade JB, et al. Rifaximin improves driving simulator performance in a randomized trial of patients with minimal hepatic encephalopathy. Gastroenterology. 2011;140(2):478-487.e1. doi:10.1053/j.gastro.2010.08.061	Information of HBV status of cohort not clear
34	Bajaj JS, Saeian K, Christensen KM, et al. Probiotic yogurt for the treatment of minimal hepatic encephalopathy. Am J Gastroenterol. 2008;103(7):1707-1715. doi:10.1111/j.1572-0241.2008.01861.x	Information of HBV status of cohort not clear
35	Bajaj JS, Wade JB, Gibson DP, et al. The multi-dimensional burden of cirrhosis and hepatic encephalopathy on patients and caregivers. Am J Gastroenterol. 2011;106(9):1646-1653. doi:10.1038/ajg.2011.157	Information of HBV status of cohort not clear

36	Bajaj JS, White MB, Unser AB, et al. Cirrhotic patients have good insight into their daily functional impairment despite prior hepatic encephalopathy: comparison with PROMIS norms. Metab Brain Dis. 2016;31(5):1199-1203. doi:10.1007/s11011-016-9860-8	Information of HBV status of cohort not clear
37	Bakhshi Aliabad MH, Jafari E, Karimi Kakh M, et al. Anxiety leads to up-regulation of CD36 on the monocytes of chronic hepatitis B-infected patients. Int J Psychiatry Med. 2016;51(5):467-475. doi:10.1177/0091217416680199	No HRQOL scale used
38	Bakshi N, Singh K. Nutrition status and its impact on quality of life and performance status in end-stage liver disease. Indian Journal of Transplantation. 2019;13(1):31-37. doi:10.4103/ijot.ijot_39_18	Information of HBV status of cohort not clear
39	Bao ZJ, Qiu DK, Ma X, et al. Assessment of health-related quality of life in Chinese patients with minimal hepatic encephalopathy. World J Gastroenterol. 2007;13(21):3003-3008. doi:10.3748/wjg.v13.i21.3003	Information of HBV status of cohort not clear
40	Basu P, Shah NJ, Farhat S, et al. PWE-127 Restless Leg Syndrome, (RLS) is Associated with Hepatic Encephalopathy (He) in Decompensated Cirrhosis. A Clinical Pilot Study. Gut. 2013;62:A182-A183. doi:10.1136/gutjnl-2013-304907.415	No HRQOL scale used
41	Baumann AJ, Wheeler DS, James M, Turner R, Siegel A, Navarro VJ. Benefit of Early Palliative Care Intervention in End-Stage Liver Disease Patients Awaiting Liver Transplantation. J Pain Symptom Manage. 2015;50(6):882-6.e2. doi:10.1016/j.jpainsymman.2015.07.014	Information of HBV status of cohort not clear
42	Bayat N, Alishiri GH, Salimzadeh A, et al. Symptoms of anxiety and depression: A comparison among patients with different chronic conditions. J Res Med Sci. 2011;16(11):1441-1447.	No HRQOL scale used
43	Belle SH, Porayko MK, Hoofnagle JH, Lake JR, Zetterman RK. Changes in quality of life after liver transplantation among adults. National Institute of Diabetes and Digestive and Kidney Diseases (NIDDK) Liver Transplantation Database (LTD). Liver Transpl Surg. 1997;3(2):93-104. doi:10.1002/lt.500030201	Information of HBV status of cohort not clear
44	Benzing C, Krezdorn N, Förster J, et al. Health-related quality of life and affective status in liver transplant recipients and patients on the waiting list with low MELD scores. HPB (Oxford). 2016;18(5):449-455. doi:10.1016/j.hpb.2016.01.546	Information of HBV status of cohort not clear
45	Benzing C, Krezdorn N, Hinz A, et al. Mental Status in Patients Before and After Liver Transplantation. Ann Transplant. 2015;20:683-693. Published 2015 Nov 17. doi:10.12659/aot.894916	No HRQOL scale used
46	Berzigotti A, Albillos A, Villanueva C, et al. Effects of an intensive lifestyle intervention program on portal hypertension in patients with cirrhosis and obesity: The SportDiet study. Hepatology. 2017;65(4):1293-1305. doi:10.1002/hep.28992	Information of HBV status of cohort not clear

47	Bhanji RA, Carey EJ, Watt KD. Review article: maximising quality of life while aspiring for quantity of life in end-stage liver disease. Aliment Pharmacol Ther. 2017;46(1):16-25. doi:10.1111/apt.14078	Review
48	Bhat M, Wyse JM, Moodie E, et al. Prevalence and predictors of sleep disturbance among liver diseases in long-term transplant survivors. Can J Gastroenterol Hepatol. 2015;29(8):440-444. doi:10.1155/2015/359640	No HRQOL scale used
49	Bianchi G, Loguercio C, Sgarbi D, et al. Reduced quality of life of patients with hepatocellular carcinoma. Dig Liver Dis. 2003;35(1):46-54. doi:10.1016/s1590-8658(02)00011-7	Information of HBV status of cohort not clear
50	Bianchi G, Marchesini G, Nicolino F, et al. Psychological status and depression in patients with liver cirrhosis. Dig Liver Dis. 2005;37(8):593-600. doi:10.1016/j.dld.2005.01.020	Information of HBV status of cohort not clear
51	Blanco Vela CI, Poo Ramírez JL. Efficacy of oral L-ornithine L-aspartate in cirrhotic patients with hyperammonemic hepatic encephalopathy. Ann Hepatol. 2011;10 Suppl 2:S55-S59.	Review
52	Borgaonkar MR, Irvine EJ. Quality of life measurement in gastrointestinal and liver disorders. Gut. 2000;47(3):444-454. doi:10.1136/gut.47.3.444	Review
53	Bruyneel M, Sersté T, Libert W, et al. Improvement of sleep architecture parameters in cirrhotic patients with recurrent hepatic encephalopathy with the use of rifaximin. Eur J Gastroenterol Hepatol. 2017;29(3):302-308. doi:10.1097/MEG.0000000000000786	Information of HBV status of cohort not clear
54	Bryce CL, Angus DC, Switala J, Roberts MS, Tsevat J. Health status versus utilities of patients with end-stage liver disease. Qual Life Res. 2004;13(4):773-782. doi:10.1023/B:QURE.0000021685.83961.88	Information of HBV status of cohort not clear
55	Buganza-Torio E, Mitchell N, Abraldes JG, et al. Depression in cirrhosis - a prospective evaluation of the prevalence, predictors and development of a screening nomogram. Aliment Pharmacol Ther. 2019;49(2):194-201. doi:10.1111/apt.15068	Information of HBV status of cohort not clear
56	Burkard T, Biedermann A, Herold C, Dietlein M, Rauch M, Diefenbach M. Treatment with a potassium-iron-phosphate-citrate complex improves PSE scores and quality of life in patients with minimal hepatic encephalopathy: a multicenter, randomized, placebo-controlled, double-blind clinical trial. Eur J Gastroenterol Hepatol. 2013;25(3):352-358. doi:10.1097/MEG.0b013e32835afaa5	Information of HBV status of cohort not clear
57	Butt Z, Parikh ND, Skaro AI, Ladner D, Cella D. Quality of life, risk assessment, and safety research in liver transplantation: new frontiers in health services and outcomes research. Curr Opin Organ Transplant. 2012;17(3):241-247. doi:10.1097/MOT.0b013e32835365c6	Review
58	Campbell MS, Brensinger CM, Sanyal AJ, et al. Quality of life in refractory ascites: transjugular intrahepatic portal-systemic shunting versus medical therapy. Hepatology. 2005;42(3):635-640. doi:10.1002/hep.20840	Information of HBV status of cohort not clear

59	Casanovas T, Herdman M, Chandía A, Peña MC, Fabregat J, Vilallonga JS. Identifying Improved and Non-improved Aspects of Health-related Quality of Life After Liver Transplantation Based on the Assessment of the Specific Questionnaire Liver Disease Quality of Life. Transplant Proc. 2016;48(1):132-137. doi:10.1016/j.transproceed.2015.11.009	Information of HBV status of cohort not clear
60	Casanovas T, Jané L, Herdman M, et al. Assessing outcomes in liver disease patients: reliability and validity of the Spanish version of the Liver Disease Quality of Life Questionnaire (LDQOL 1.0). Value Health. 2010;13(4):455-462. doi:10.1111/j.1524-4733.2009.00688.x	Information of HBV status of cohort not clear
61	Casanovas Taltavull T, Chandia Frias A, Villallonga Vilarmau JS, et al. Prospective validation of the spanish version of the Short Form-Liver Disease Quality of Life. J Gastrohep. 2016;39(4):243-254. doi:10.1016/j.gastrohep.2015.08.001	Utility scores not reported
62	Casanovas Taltavull T, Jané Cabré L, Herdman M, Casado Collado A, Pubill BP, Fabregat Prous J. Validation of the Spanish version of the liver disease quality of life instrument among candidates for liver transplant. Transplant Proc. 2007;39(7):2274-2277. doi:10.1016/j.transproceed.2007.07.006	Information of HBV status of cohort not clear
63	Cavaggioni G, Poli E, Ferri F, et al. MELDNa score is associated with psychopathology and reduced quality of life in cirrhotic patients with a liver transplant perspective. Metab Brain Dis. 2017;32(3):923-933. doi:10.1007/s11011-017-9987-2	Information of HBV status of cohort not clear
64	Chan H, Yu CS, Li SY. Psychiatric morbidity in Chinese patients with chronic hepatitis B infection in a local infectious disease clinic. East Asian Arch Psychiatry. 2012;22(4):160-168.	No HRQOL scale used
65	Chang PE, Tan HK, Lee Y, et al. Clinical validation of the chronic liver disease questionnaire for the Chinese population in Singapore. JGH Open. 2019;4(2):191-197. doi:10.1002/jgh3.12239	Information of HBV status of cohort not clear
66	Chantrakul R, Kaewdech A, Sripongpun P, Nachamroonkul N, Phisalprapa P. Health-related quality of life in Thai chronic hepatitis B patients. Hepatology International. 2022;16(S1):S179. doi:10.1007/s12072-022-10337-4	Abstract
67	Chao J, Song L, Zhang H, et al. Effects of comprehensive intervention on health-related quality of life in patients with chronic hepatitis B in China. BMC Health Serv Res. 2013;13:386. Published 2013 Oct 4. doi:10.1186/1472-6963-13-386	Used HRQOL scale without composite utility value (SF-36)
68	Chatrath H, Liangpunsakul S, Ghabril M, Otte J, Chalasani N, Vuppalanchi R. Prevalence and morbidity associated with muscle cramps in patients with cirrhosis. Am J Med. 2012;125(10):1019-1025. doi:10.1016/j.amjmed.2012.03.012	Information of HBV status of cohort not clear
69	Chen F, Pang X, Dai X. Effect of comprehensive nursing on the pain, anxiety and malnutrition of hepatitis B patients. Am J Transl Res. 2021;13(5):4656-4665.	Utility scores not reported

70	Chen MC, Hung HC, Chang HJ, Yang SS, Tsai WC, Chang SC. Assessment of Educational Needs and Quality of Life of Chronic Hepatitis Patients. BMC Health Serv Res. 2017;17(1):148. Published 2017 Feb 17. doi:10.1186/s12913-017-2082-x	Information of HBV status of cohort not clear
71	Chen ZJ, Al-Mahtab M, Rahman S, Tan NC, Luo N, Tai BC. Validity and reliability of the Bengali version of the Hepatitis Quality of Life Questionnaire. Qual Life Res. 2010;19(9):1343-1348. doi:10.1007/s11136-010-9702-9	Used HRQOL scale without composite utility value (HQLQ)
72	Chien YC, Chiang HC, Lin PY, Chen YL. Erectile function in men with end-stage liver disease improves after living donor liver transplantation. BMC Urol. 2015;15:83. Published 2015 Aug 13. doi:10.1186/s12894-015-0078-6	No HRQOL scale used
73	Cole CR, Bucuvalas JC, Hornung RW, et al. Impact of liver transplantation on HRQOL in children less than 5 years old. Pediatr Transplant. 2004;8(3):222-227. doi:10.1111/j.1399-3046.2004.00126.x	Information of HBV status of cohort not clear
74	Compañy L, Zapater P, Pérez-Mateo M, Jover R. Extrapyramidal signs predict the development of overt hepatic encephalopathy in patients with liver cirrhosis. Eur J Gastroenterol Hepatol. 2010;22(5):519-525. doi:10.1097/MEG.0b013e328333df0f	Information of HBV status of cohort not clear
75	Córdoba J, Cabrera J, Lataif L, Penev P, Zee P, Blei AT. High prevalence of sleep disturbance in cirrhosis. Hepatology. 1998;27(2):339-345. doi:10.1002/hep.510270204	No HRQOL scale used
76	Corruble E, Barry C, Varescon I, et al. Report of depressive symptoms on waiting list and mortality after liver and kidney transplantation: a prospective cohort study. BMC Psychiatry. 2011;11:182. Published 2011 Nov 21. doi:10.1186/1471-244X-11-182	No HRQOL scale used
77	Corruble E, Barry C, Varescon I, Falissard B, Castaing D, Samuel D. Depressive symptoms predict long-term mortality after liver transplantation. J Psychosom Res. 2011;71(1):32-37. doi:10.1016/j.jpsychores.2010.12.008	No HRQOL scale used
78	Costache C, Popescu E, Angelescu G, et al. Journal of Gastrointestinal and Liver Diseases. 2019;28(S2):63. doi:10.15403/jgld.2014.1121.281.hpc	Information of HBV status of cohort not clear
79	Cowling T, Jennings LW, Goldstein RM, et al. Liver transplantation and health-related quality of life: scoring differences between men and women. Liver Transpl. 2004;10(1):88-96. doi:10.1002/lt.20013	Information of HBV status of cohort not clear
80	Cron DC, Friedman JF, Winder GS, et al. Depression and Frailty in Patients With End-Stage Liver Disease Referred for Transplant Evaluation. Am J Transplant. 2016;16(6):1805-1811. doi:10.1111/ajt.13639	Information of HBV status of cohort not clear
81	Dąbrowska-Bender M, Michałowicz B, Pączek L. Assessment of the Quality of Life in Patients After Liver Transplantation as an Important Part of Treatment Results. Transplant Proc. 2016;48(5):1697-1702. doi:10.1016/j.transproceed.2015.12.139	Information of HBV status of cohort not clear

82	De Bona M, Ponton P, Ermani M, et al. The impact of liver disease and medical complications on quality of life and psychological distress before and after liver transplantation. J Hepatol. 2000;33(4):609-615. doi:10.1034/j.1600-0641.2000.033004609.x	Information of HBV status of cohort not clear
83	Deng X, Liang J, Wu FS, Li YB, Tang YF. Effects of the Ganning formula on liver fibrosis in patients with chronic hepatitis B. J Tradit Chin Med. 2011;31(4):282-287. doi:10.1016/s0254-6272(12)60005-3	Used HRQOL scale without composite utility value (WHOQOL-BREF)
84	Denny B, Beyerle K, Kienhuis M, Cora A, Gavidia-Payne S, Hardikar W. New insights into family functioning and quality of life after pediatric liver transplantation. Pediatr Transplant. 2012;16(7):711-715. doi:10.1111/j.1399-3046.2012.01738.x	Information of HBV status of cohort not clear
85	Denny B, Kienhuis M, Gavidia-Payne S. Explaining the quality of life of organ transplant patients by using crisis theory. Prog Transplant. 2015;25(4):324-331. doi:10.7182/pit2015904	Information of HBV status of cohort not clear
86	Derck JE, Thelen AE, Cron DC, et al. Quality of life in liver transplant candidates: frailty is a better indicator than severity of liver disease. Transplantation. 2015;99(2):340-344. doi:10.1097/TP.0000000000000593	Information of HBV status of cohort not clear
87	Desai AP, Hobbs L, McCarty J, et al. Improving the interpretation of health-related quality of life scores by establishing minimal important differences in promis scale scores in liver disease. Hepatology. 2021;74(S1):391A-392A. doi:10.1002/hep.32188	No HRQOL scale used
88	Dias Teixeira MC, de Fátima Gomes de Sá Ribeiro M, Strauss E. A new insight into the differences among non-cirrhotic and cirrhotic patients using the liver disease quality of life instrument (LDQOL). Ann Hepatol. 2005;4(4):264-271.	Information of HBV status of cohort not clear
89	Díaz-Domínguez R, Pérez-Bernal J, Pérez-San-Gregorio MA, Martín-Rodríguez A. Quality of life in patients with kidney, liver or heart failure during the waiting list period. Transplant Proc. 2006;38(8):2459-2461. doi:10.1016/j.transproceed.2006.08.014	Information of HBV status of cohort not clear
90	Dirks M, Haag K, Pflugrad H, et al. Neuropsychiatric symptoms in hepatitis C patients resemble those of patients with autoimmune liver disease but are different from those in hepatitis B patients. J Viral Hepat. 2019;26(4):422-431. doi:10.1111/jvh.12979	Utility scores not reported
91	Dittner AJ, Wessely SC, Brown RG. The assessment of fatigue: a practical guide for clinicians and researchers. J Psychosom Res. 2004;56(2):157-170. doi:10.1016/S0022-3999(03)00371-4	Review
92	Domingos MF, Coelho JCU, Nogueira IR, et al. Quality of Life after 10 Years of Liver Transplantation. J Gastrointestin Liver Dis. 2020;29(4):611-616. Published 2020 Oct 27. doi:10.15403/jgld-2829	Information of HBV status of cohort not clear

93	dos Santos DC, Limongi V, de Oliveira da Silva AM, et al. Evaluation of functional status, pulmonary capacity, body composition, and quality of life of end-stage liver disease patients who are candidates for liver surgery. Transplant Proc. 2014;46(6):1771-1774. doi:10.1016/j.transproceed.2014.05.043	Information of HBV status of cohort not clear
94	Eaton CK, Lee JL, Loiselle KA, et al. Pretransplant patient, parent, and family psychosocial functioning varies by organ type and patient age. Pediatr Transplant. 2016;20(8):1137-1147. doi:10.1111/petr.12826	Information of HBV status of cohort not clear
95	Elliott C, Frith J, Pairman J, Jones DE, Newton JL. Reduction in functional ability is significant postliver transplantation compared with matched liver disease and community dwelling controls. Transpl Int. 2011;24(6):588-595. doi:10.1111/j.1432-2277.2011.01240.x	Information of HBV status of cohort not clear
96	Ellul MA, Gholkar SA, Cross TJ. Hepatic encephalopathy due to liver cirrhosis. BMJ. 2015;351:h4187. Published 2015 Aug 11. doi:10.1136/bmj.h4187	Review
97	Eltawil KM, Berry R, Abdolell M, Molinari M. Quality of life and survival analysis of patients undergoing transarterial chemoembolization for primary hepatic malignancies: a prospective cohort study. HPB (Oxford). 2012;14(5):341-350. doi:10.1111/j.1477-2574.2012.00455.x	Information of HBV status of cohort not clear
98	Enescu A, Mitrut P, Balasoiu M, Turculeanu A, Enescu AS. Psychosocial issues in patients with chronic hepatitis B and C. Curr Health Sci J. 2014;40(2):93-96. doi:10.12865/CHSJ.40.02.02	Review
99	Eraydın A, Akarsu M, Derviş Hakim G, Keskinoğlu P, Ellidokuz H. The validity and reliability of "The liver disease symptom index 2.0" for Turkish society. Turk J Gastroenterol. 2014;25(5):531-538. doi:10.5152/tjg.2014.7509	Information of HBV status of cohort not clear
100	Estraviz B, Quintana JM, Valdivieso A, et al. Factors influencing change in health-related quality of life after liver transplantation. Clin Transplant. 2007;21(4):481-499. doi:10.1111/j.1399-0012.2007.00672.x	Information of HBV status of cohort not clear
101	Evon DM, Lin HS, Khalili M, et al. Patient-reported outcomes in a large North American cohort living with chronic hepatitis B virus: a cross-sectional analysis. Aliment Pharmacol Ther. 2020;51(4):457-468. doi:10.1111/apt.15618	Used HRQOL scale without composite utility value (SF-36)
102	Evon DM, Wahed AS, Johnson G, et al. Fatigue in Patients with Chronic Hepatitis B Living in North America: Results from the Hepatitis B Research Network (HBRN). Dig Dis Sci. 2016;61(4):1186-1196. doi:10.1007/s10620-015-4006-0	No HRQOL scale used
103	Fallon MB, Krowka MJ, Brown RS, et al. Impact of hepatopulmonary syndrome on quality of life and survival in liver transplant candidates. Gastroenterology. 2008;135(4):1168-1175. doi:10.1053/j.gastro.2008.06.038	Information of HBV status of cohort not clear

104	Fazekas C, Matzer F, Kniepeiss D, et al. Health related quality of life, workability and return to work of patients after liver transplantation. Transplant International. 2021;34(S3):30. doi:10.1111/tri.14036	Utility scores not reported
105	Ferreira FA, de Almeida-Neto C, Teixeira MC, Strauss E. Health-related quality of life among blood donors with hepatitis B and hepatitis C: longitudinal study before and after diagnosis. Rev Bras Hematol Hemoter. 2015;37(6):381-387. doi:10.1016/j.bjhh.2015.08.004	No HRQOL scale used
106	Findikli E, Ates S, Kandemir B, et al. A case-control study on the temperament and Psychological mood of patients with chronic Hepatitis B. Eur J Gen Med. 2017;14(3):58-62. doi:10.29333/ejgm/81887	No HRQOL scale used
107	Foster C, Baki J, Nikirk S, Williams S, Parikh ND, Tapper EB. Comprehensive Health-State Utilities in Contemporary Patients With Cirrhosis. Hepatol Commun. 2020;4(6):852-858. Published 2020 Apr 2. doi:10.1002/hep4.1512	Information of HBV status of cohort not clear
108	Fransiska D, Hutami HT, Purnomo HD. Patient Reported Outcome (PRO) of Chronic Hepatitis B Patients: Case study in Dr. Kariadi General Hospital Medical Center. Hepatology International. 2020;14(S1):S87-S88. doi:10.1007/s12072-020-10030-4	Abstract
109	Fukunishi I, Kita Y, Wakabayashi T, et al. Health status survey of adult patients undergoing living-related liver transplantation. Transplant Proc. 2000;32(7):2149-2151. doi:10.1016/s0041-1345(00)01611-0	Information of HBV status of cohort not clear
110	Gad YZ, Zaher AA, Moussa NH, El-desoky AE, Al-Adarosy HA. Screening for minimal hepatic encephalopathy in asymptomatic drivers with liver cirrhosis. Arab J Gastroenterol. 2011;12(2):58-61. doi:10.1016/j.ajg.2011.04.002	No HRQOL scale used
111	Galant LH, Forgiarini Junior LA, Dias AS, Marroni CA. Functional status, respiratory muscle strength, and quality of life in patients with cirrhosis. Rev Bras Fisioter. 2012;16(1):30-34.	Utility scores not reported
112	Gandhi S, Khubchandani S, Iyer R. Quality of life and hepatocellular carcinoma. J Gastrointest Oncol. 2014;5(4):296-317. doi:10.3978/j.issn.2078-6891.2014.046	Review
113	Gao F, Gao R, Li G, Shang ZM, Hao JY. Health-related quality of life and survival in Chinese patients with chronic liver disease. Health Qual Life Outcomes. 2013;11:131. Published 2013 Aug 1. doi:10.1186/1477-7525-11-131	Information of HBV status of cohort not clear
114	Gao R, Gao F, Li G, Hao JY. Health-related quality of life in chinese patients with chronic liver disease. Gastroenterol Res Pract. 2012;2012:516140. doi:10.1155/2012/516140	Information of HBV status of cohort not clear
115	Gazineo D, Godino L, Bui V, et al. Health-related quality of life in outpatients with chronic liver disease: a cross-sectional study. BMC Gastroenterol. 2021;21(1):318. Published 2021 Aug 7. doi:10.1186/s12876-021-01890-7	Information of HBV status of cohort not clear

116	Girgenti R, Tropea A, Buttafarro MA, Ragusa R, Ammirata M. Quality of Life in Liver Transplant Recipients: A Retrospective Study. Int J Environ Res Public Health. 2020;17(11):3809. Published 2020 May 27. doi:10.3390/ijerph17113809	Information of HBV status of cohort not clear
117	Girgrah N, Reid G, MacKenzie S, Wong F. Cirrhotic cardiomyopathy: does it contribute to chronic fatigue and decreased health-related quality of life in cirrhosis?. Can J Gastroenterol. 2003;17(9):545-551. doi:10.1155/2003/213213	Information of HBV status of cohort not clear
118	Goel A, Arivazhagan K, Sasi A, et al. Translation and validation of chronic liver disease questionnaire (CLDQ) in Tamil language. Indian J Gastroenterol. 2017;36(3):217-226. doi:10.1007/s12664-017-0756-2	Information of HBV status of cohort not clear
119	Goel A, Jat SL, Sasi A, Paliwal VK, Aggarwal R. Prevalence, severity, and impact on quality of life of restless leg syndrome in patients with liver cirrhosis in India. Indian J Gastroenterol. 2016;35(3):216-221. doi:10.1007/s12664-016-0668-6	Information of HBV status of cohort not clear
120	Goosmann L, Buchholz A, Bangert K, et al. Liver transplantation for acute-on-chronic liver failure predicts post-transplant mortality and impaired long-term quality of life. Liver Int. 2021;41(3):574-584. doi:10.1111/liv.14756	Information of HBV status of cohort not clear
121	Gotardo DR, Strauss E, Teixeira MC, Machado MC. Liver transplantation and quality of life: relevance of a specific liver disease questionnaire. Liver Int. 2008;28(1):99-106. doi:10.1111/j.1478-3231.2007.01606.x	Information of HBV status of cohort not clear
122	Gralnek IM, Hays RD, Kilbourne A, et al. Development and evaluation of the Liver Disease Quality of Life instrument in persons with advanced, chronic liver disease--the LDQOL 1.0. Am J Gastroenterol. 2000;95(12):3552-3565. doi:10.1111/j.1572-0241.2000.03375.x	Information of HBV status of cohort not clear
123	Gritti A, Pisano S, Salvati T, Di Cosmo N, Iorio R, Vajro P. Health-related quality of life in pediatric liver transplanted patients compared with a chronic liver disease group. Ital J Pediatr. 2013;39:55. Published 2013 Sep 11. doi:10.1186/1824-7288-39-55	Information of HBV status of cohort not clear
124	Groeneweg M, Moerland W, Quero JC, Hop WC, Krabbe PF, Schalm SW. Screening of subclinical hepatic encephalopathy. J Hepatol. 2000;32(5):748-753. doi:10.1016/s0168-8278(00)80243-3	Information of HBV status of cohort not clear
125	Groeneweg M, Quero JC, De Bruijn I, et al. Subclinical hepatic encephalopathy impairs daily functioning. Hepatology. 1998;28(1):45-49. doi:10.1002/hep.510280108	Information of HBV status of cohort not clear
126	Guest JF, Nanuwa K, Barden R. Utility values for specific hepatic encephalopathy health states elicited from the general public in the United Kingdom. Health Qual Life Outcomes. 2014;12:89. Published 2014 Jun 10. doi:10.1186/1477-7525-12-89	Information of HBV status of cohort not clear

127	Gutteling JJ, Busschbach JJ, de Man RA, Darlington AS. Logistic feasibility of health related quality of life measurement in clinical practice: results of a prospective study in a large population of chronic liver patients. Health Qual Life Outcomes. 2008;6:97. Published 2008 Nov 10. doi:10.1186/1477-7525-6-97	Information of HBV status of cohort not clear
128	Gutteling JJ, de Man RA, Busschbach JJ, Darlington AS. Overview of research on health-related quality of life in patients with chronic liver disease. Neth J Med. 2007;65(7):227-234.	Review
129	Gutteling JJ, de Man RA, van der Plas SM, Schalm SW, Busschbach JJ, Darlington AS. Determinants of quality of life in chronic liver patients. Aliment Pharmacol Ther. 2006;23(11):1629-1635. doi:10.1111/j.1365-2036.2006.02934.x	Information of HBV status of cohort not clear
130	Ha TT, Pham MD, Trang NA. Assessment pre- and post-treatment quality of life by eortc qlq-30 questionnaire in patients with hepatocellular carcinoma at hanoi medical university, vietnam. Gut. 2021;70(S2):A78. doi:10.1136/gutjnl-2021-IDDF.83	Information of HBV status of cohort not clear
131	Haag S, Senf W, Häuser W, et al. Impairment of health-related quality of life in functional dyspepsia and chronic liver disease: the influence of depression and anxiety. Aliment Pharmacol Ther. 2008;27(7):561-571. doi:10.1111/j.1365-2036.2008.03619.x	Information of HBV status of cohort not clear
132	Hansen L, Chang MF, Lee CS, et al. Physical and Mental Quality of Life in Patients With End-Stage Liver Disease and Their Informal Caregivers. Clin Gastroenterol Hepatol. 2021;19(1):155-161.e1. doi:10.1016/j.cgh.2020.04.014	Information of HBV status of cohort not clear
133	Hartrumpf KJ, Marquardt S, Werncke T, et al. Quality of life in patients undergoing repetitive TACE for the treatment of intermediate stage HCC. J Cancer Res Clin Oncol. 2018;144(10):1991-1999. doi:10.1007/s00432-018-2704-7	Information of HBV status of cohort not clear
134	Hauser W, Shnur M, Steder-Neukamm U, Muthny FA, Grandt D. Validation of the German version of the Chronic Liver Disease Questionnaire. European Journal of Gastroenterology & Hepatology. 2004;16:599-606. doi:10.1097/01.meg.0000108324.52416.18	Information of HBV status of cohort not clear
135	Heidarzadeh A, Yousefi-Mashhour M, Mansour-Ghanaei F, et al. Quality of Life in Chronic Hepatitis B and C Patients. Hep Man. 2007;7(2):67-72.	No HRQOL scale used
136	Hickman IJ, Jonsson JR, Prins JB, et al. Modest weight loss and physical activity in overweight patients with chronic liver disease results in sustained improvements in alanine aminotransferase, fasting insulin, and quality of life. Gut. 2004;53(3):413-419. doi:10.1136/gut.2003.027581	Information of HBV status of cohort not clear
137	Hinrichs JB, Hasdemir DB, Nordlohne M, et al. Health-Related Quality of Life in Patients with Hepatocellular Carcinoma Treated with Initial Transarterial Chemoembolization. Cardiovasc Intervent Radiol. 2017;40(10):1559-1566. doi:10.1007/s00270-017-1681-6	Information of HBV status of cohort not clear

138	Huang E, Esrailian E, Spiegel BM. The cost-effectiveness and budget impact of competing therapies in hepatic encephalopathy - a decision analysis. Aliment Pharmacol Ther. 2007;26(8):1147-1161. doi:10.1111/j.1365-2036.2007.03464.x	No HRQOL scale used
139	Huang G, Chen X, Lau WY, et al. Quality of life after surgical resection compared with radiofrequency ablation for small hepatocellular carcinomas. Br J Surg. 2014;101(8):1006-1015. doi:10.1002/bjs.9539	Utility scores not reported
140	Huang HC, Lin KC, Wu CS, Miao NF, Chen MY. Health-promoting behaviors benefit the mental health of cirrhotic outpatients. Qual Life Res. 2018;27(6):1521-1532. doi:10.1007/s11136-018-1818-3	Utility scores not reported
141	Huang YW, Hu JT, Hu FC, et al. Biphasic pattern of depression and its predictors during pegylated interferon-based therapy in chronic hepatitis B and C patients. Antivir Ther. 2013;18(4):567-573. doi:10.3851/IMP2441	No HRQOL scale used
142	Hui Y, Li N, Yu Z, et al. Health-Related Quality of Life and Its Contributors According to a Preference-Based Generic Instrument in Cirrhosis. Hepatol Commun. 2022;6(3):610-620. doi:10.1002/hep4.1827	Information of HBV status of cohort not clear
143	Iorio R, Pensati P, Botta S, et al. Side effects of alpha-interferon therapy and impact on health-related quality of life in children with chronic viral hepatitis. Pediatr Infect Dis J. 1997;16(10):984-990. doi:10.1097/00006454-199710000-00016	Information of HBV status of cohort not clear
144	Iwasa M, Karino Y, Kawaguchi T, et al. Relationship of muscle cramps to quality of life and sleep disturbance in patients with chronic liver diseases: A nationwide study. Liver Int. 2018;38(12):2309-2316. doi:10.1111/liv.13745	Utility scores not reported
145	Jalan R, Gooday R, O'Carroll RE, Redhead DN, Elton RA, Hayes PC. A prospective evaluation of changes in neuropsychological and liver function tests following transjugular intrahepatic portosystemic stent-shunt. J Hepatol. 1995;23(6):697-705. doi:10.1016/0168-8278(95)80036-0	Information of HBV status of cohort not clear
146	Janani K, Jain M, Vargese J, et al. Health-related quality of life in liver cirrhosis patients using SF-36 and CLDQ questionnaires. Clin Exp Hepatol. 2018;4(4):232-239. doi:10.5114/ceh.2018.80124	Information of HBV status of cohort not clear
147	Janani K, Varghese J, Jain M, et al. HRQOL using SF36 (generic specific) in liver cirrhosis. Indian J Gastroenterol. 2017;36(4):313-317. doi:10.1007/s12664-017-0773-1	Information of HBV status of cohort not clear
148	Jang Y, Ahn SH, Lee K, Lee J, Kim JH. Psychometric evaluation of the Korean version of the Hepatitis B Quality of Life Questionnaire. PLoS One. 2019;14(2):e0213001. Published 2019 Feb 27. doi:10.1371/journal.pone.0213001	Utility scores not reported
149	Jang Y, Boo S, Yoo H. Hepatitis B Virus Infection: Fatigue-Associated Illness Experiences Among Koreans. Gastroenterol	No HRQOL scale used

	Nurs. 2018;41(5):388-395. doi:10.1097/SGA.0000000000000335	
150	Jang Y, Kim JH, Lee K. Validation of the revised piper fatigue scale in Koreans with chronic hepatitis B. PLoS One. 2017;12(5):e0177690. Published 2017 May 23. doi:10.1371/journal.pone.0177690	No HRQOL scale used
151	Janssen MF, Pickard AS, Golicki D, et al. Measurement properties of the EQ-5D-5L compared to the EQ-5D-3L across eight patient groups: a multi-country study. Qual Life Res. 2013;22(7):1717-1727. doi:10.1007/s11136-012-0322-4	Information of HBV status of cohort not clear
152	Jara M, Bednarsch J, Malinowski M, et al. Predictors of quality of life in patients evaluated for liver transplantation. Clin Transplant. 2014;28(12):1331-1338. doi:10.1111/ctr.12426	Information of HBV status of cohort not clear
153	Jerson B, D'Urso C, Arnon R, et al. Adolescent transplant recipients as peer mentors: a program to improve self-management and health-related quality of life. Pediatr Transplant. 2013;17(7):612-620. doi:10.1111/petr.12127	Information of HBV status of cohort not clear
154	Jing LU et al. Correlation of quality of life with aspartate aminotransferase-to-platelet ratio index, liver stiffness measurement, and histopathology after antiviral therpay for chronic hepatitis B liver fibrosis. Journal of Clinical Hepatology. 2021;37(4). doi:10.3969/j.issn.1001-5256.2021.04.018	Utility scores not reported
155	Josefsson A, Fu M, Björnsson E, Castedal M, Kalaitzakis E. Impact of cardiac dysfunction on health-related quality of life in cirrhotic liver transplant candidates. Eur J Gastroenterol Hepatol. 2015;27(4):393-398. doi:10.1097/MEG.0000000000000292	Information of HBV status of cohort not clear
156	Jutagir DR, Saracino RM, Cunningham A, et al. The feasibility of a group stress management Liver SMART intervention for patients with end-stage liver disease: A pilot study. Palliat Support Care. 2019;17(1):35-41. doi:10.1017/S147895151800024X	Information of HBV status of cohort not clear
157	Jyoti, Saklani M, Sarin J. Quality of Life Among Chronci Liver Disease Patients: An Exploratory Cross Sectional Survey. Indian Journal of Forensic Medicine & Toxicology. 2021;15(2). doi:10.37506/ijfmt.v15i2.14296	Information of HBV status of cohort not clear
158	Kalaitzakis E, Josefsson A, Björnsson E. Type and etiology of liver cirrhosis are not related to the presence of hepatic encephalopathy or health-related quality of life: a cross-sectional study. BMC Gastroenterol. 2008;8:46. Published 2008 Oct 15. doi:10.1186/1471-230X-8-46	Information of HBV status of cohort not clear
159	Kalaitzakis E, Josefsson A, Castedal M, et al. Factors related to fatigue in patients with cirrhosis before and after liver transplantation. Clin Gastroenterol Hepatol. 2012;10(2):174-181.e1. doi:10.1016/j.cgh.2011.07.029	Information of HBV status of cohort not clear

160	Kanwal F, Gralnek IM, Hays RD, et al. Health-related quality of life predicts mortality in patients with advanced chronic liver disease. Clin Gastroenterol Hepatol. 2009;7(7):793-799. doi:10.1016/j.cgh.2009.03.013	Information of HBV status of cohort not clear
161	Kanwal F, Hays RD, Kilbourne AM, Dulai GS, Gralnek IM. Are physician-derived disease severity indices associated with health-related quality of life in patients with end-stage liver disease?. Am J Gastroenterol. 2004;99(9):1726-1732. doi:10.1111/j.1572-0241.2004.30300.x	Information of HBV status of cohort not clear
162	Kanwal F. Health Related Quality of Life in Patients with Cirrhosis. Current Hepatology Reports. 2014;13(1):8-13. doi:10.1007/s11901-014-0218-6	Review
163	Karaivazoglou K, Iconomou G, Triantos C, et al. Fatigue and depressive symptoms associated with chronic viral hepatitis patients. health-related quality of life (HRQOL). Ann Hepatol. 2010;9(4):419-427.	Used HRQOL scale without composite utility value (SF-36)
164	Kato A, Tanaka H, Kawaguchi T, et al. Nutritional management contributes to improvement in minimal hepatic encephalopathy and quality of life in patients with liver cirrhosis: A preliminary, prospective, open-label study. Hepatol Res. 2013;43(5):452-458. doi:10.1111/j.1872-034X.2012.01092.x	Information of HBV status of cohort not clear
165	Kawashima Vasconcelos MY, Lopes ARF, Mente ÊD, Castro-E-Silva O, Galvão CM, Dal Sasso-Mendes K. Chronic Liver Disease Questionnaire as a Tool to Evaluate the Quality of Life in Liver Transplant Candidates [published online ahead of print, 2018 Dec 25]. Prog Transplant. 2018;1526924818817053. doi:10.1177/1526924818817053	Information of HBV status of cohort not clear
166	Kensinger CD, Feurer ID, O'Dell HW, et al. Patient-reported outcomes in liver transplant recipients with hepatocellular carcinoma. Clin Transplant. 2016;30(9):1036-1045. doi:10.1111/ctr.12785	Information of HBV status of cohort not clear
167	Keown PA, Shackleton CR, Ferguson BM. The influence of long-term morbidity on health status and rehabilitation following paediatric organ transplantation. Eur J Pediatr. 1992;151 Suppl 1:S70-S75. doi:10.1007/BF02125807	Review
168	Keskin G, Gümüs AB, Orgun F. Quality of life, depression, and anxiety among hepatitis B patients. Gastroenterol Nurs. 2013;36(5):346-356. doi:10.1097/SGA.0b013e3182a788cc	Used HRQOL scale without composite utility value (WHOQOL-BREF)
169	Khairullah S, Mahadeva S. Translation, adaptation and validation of two versions of the Chronic Liver Disease Questionnaire in Malaysian patients for speakers of both English and Malay languages: a cross-sectional study. BMJ Open. 2017;7(5):e013873. Published 2017 May 25. doi:10.1136/bmjopen-2016-013873	Information of HBV status of cohort not clear

170	Kilic ZM, Kuran S, Akdogan M, et al. The long-term effects of lamivudine treatment in patients with HBeAg-negative liver cirrhosis. Adv Ther. 2008;25(3):190-200. doi:10.1007/s12325-008-0038-6	No HRQOL scale used
171	Kim HJ, Chu H, Lee S. Factors influencing on health-related quality of life in South Korean with chronic liver disease. Health Qual Life Outcomes. 2018;16(1):142. Published 2018 Jul 18. doi:10.1186/s12955-018-0964-1	Utility scores not reported
172	Kim M, Kim SY, Rou WS, Hwang SW, Lee BS. Erectile dysfunction in patients with liver disease related to chronic hepatitis B. Clin Mol Hepatol. 2015;21(4):352-357. doi:10.3350/cmh.2015.21.4.352	No HRQOL scale used
173	Klein J, Tran SN, Mentha-Dugerdil A, et al. Assessment of sexual function and conjugal satisfaction prior to and after liver transplantation. Ann Transplant. 2013;18:136-145. Published 2013 Mar 26. doi:10.12659/AOT.883860	No HRQOL scale used
174	Kok B, Whitlock R, Ferguson T, et al. Health-Related Quality of Life: A Rapid Predictor of Hospitalization in Patients With Cirrhosis. Am J Gastroenterol. 2020;115(4):575-583. doi:10.14309/ajg.0000000000000545	Information of HBV status of cohort not clear
175	Kondo Y, Yoshida H, Tateishi R, et al. Health-related quality of life of chronic liver disease patients with and without hepatocellular carcinoma. J Gastroenterol Hepatol. 2007;22(2):197-203. doi:10.1111/j.1440-1746.2006.04456.x	Information of HBV status of cohort not clear
176	Koskinas J, Merkouraki P, Manesis E, Hadziyannis S. Assessment of depression in patients with chronic hepatitis: effect of interferon treatment. Dig Dis. 2002;20(3-4):284-288. doi:10.1159/000067682	No HRQOL scale used
177	Kotarska K, Raszeja-Wyszomirska J, Wunsch E, et al. Relationship between pretransplantation liver status and health-related quality of life after grafting: a single-center prospective study. Transplant Proc. 2014;46(8):2770-2773. doi:10.1016/j.transproceed.2014.09.005	Information of HBV status of cohort not clear
178	Kruger C, McNeely ML, Bailey RJ, et al. Home Exercise Training Improves Exercise Capacity in Cirrhosis Patients: Role of Exercise Adherence. Sci Rep. 2018;8(1):99. Published 2018 Jan 8. doi:10.1038/s41598-017-18320-y	Information of HBV status of cohort not clear
179	Küçükbayrak A, Canan F, Alcelik A, et al. Evaluation of psychiatric morbidity and quality of life in inactive HbsAg carriers. African Journal of Microbiology Research. 2011;5(24):4212-4217. doi:10.5897/AJMR11.838	Used HRQOL scale without composite utility value (WHOQOL-BREF)
180	Kulthanan K, Chularojanamontri L, Tuchinda P, Rujitharanawong C, Baiardini I, Braido F. Minimal clinical important difference (MCID) of the Thai Chronic Urticaria Quality of Life Questionnaire (CU-Q2oL). Asian Pac J Allergy Immunol. 2016;34(2):137-145. doi:10.12932/AP0674.34.2.2016	Information of HBV status of cohort not clear

181	Kumar A, Pande G, Negi TS, Saraswat VA. Treatment of erectile dysfunction with PDE 5 inhibitor Tadalafil improves quality of life in male patients with compensated chronic liver disease and may reduce liver fibrosis. Journal of Gastroenterology and Hepatology. 2019;34(S3):279. doi:10.1111/jgh.14879	Utility scores not reported
182	Kunkel EJ, Kim JS, Hann HW, et al. Depression in Korean immigrants with hepatitis B and related liver diseases. Psychosomatics. 2000;41(6):472-480. doi:10.1176/appi.psy.41.6.472	No HRQOL scale used
183	Kuo SZ, Lizaola B, Hayssen H, Lai JC. Beta-blockers and physical frailty in patients with end-stage liver disease. World J Gastroenterol. 2018;24(33):3770-3775. doi:10.3748/wjg.v24.i33.3770	Information of HBV status of cohort not clear
184	Łaba M, Pszenny A, Gutowska D, et al. Quality of life after liver transplantation--preliminary report. Ann Transplant. 2008;13(4):67-71.	Information of HBV status of cohort not clear
185	Labenz C, Toenges G, Schattenberg JM, et al. Health-related quality of life in patients with compensated and decompensated liver cirrhosis. Eur J Intern Med. 2019;70:54-59. doi:10.1016/j.ejim.2019.09.004	Information of HBV status of cohort not clear
186	Labidi A, Hidri S, Hafi M, Serghini M, Zouiten L, Boubaker J. Health-related quality of life in cirrhotic patients: a case-control study. Tunis Med. 2019;97(8-9):990-996.	Utility scores not reported
187	Lark ME, Anderson M, Jabour S, et al. Post-Transplantation Frailty Is Associated with Diminished Quality of Life in Liver Transplant Recipients. Journal of the American College of Surgeons. 2021;233(5S1):S272-S273. doi:10.1016/j.jamcollsurg.2021.07.564	Information of HBV status of cohort not clear
188	Lauridsen MM, Schaffalitzky de Muckadell OB, Vilstrup H. Minimal hepatic encephalopathy characterized by parallel use of the continuous reaction time and portosystemic encephalopathy tests. Metab Brain Dis. 2015;30(5):1187-1192. doi:10.1007/s11011-015-9688-7	Information of HBV status of cohort not clear
189	Le Strat Y, Le Foll B, Dubertret C. Major depression and suicide attempts in patients with liver disease in the United States. Liver Int. 2015;35(7):1910-1916. doi:10.1111/liv.12612	Information of HBV status of cohort not clear
190	Lee K, Otgonsuren M, Younoszai Z, Mir HM, Younossi ZM. Association of chronic liver disease with depression: a population-based study. Psychosomatics. 2013;54(1):52-59. doi:10.1016/j.psym.2012.09.005	No HRQOL scale used
191	Lee MK, Chung WJ. Relationship between symptoms and both stage of change in adopting a healthy life style and quality of life in patients with liver cirrhosis: a cross-sectional study. Health Qual Life Outcomes. 2021;19(1):148. doi:10.1186/s12955-021-01787-9	Utility scores not reported
192	Les I, Doval E, Flavià M, et al. Quality of life in cirrhosis is related to potentially treatable factors. Eur J Gastroenterol Hepatol. 2010;22(2):221-227. doi:10.1097/MEG.0b013e3283319975	Information of HBV status of cohort not clear

193	Li X, Gan D, Li Y, et al. JianPi HuaZhuo XingNao formula (Chinese herbal medicine) for the treatment of minimal hepatic encephalopathy: a protocol for a randomized, placebo-controlled pilot trial. Medicine (Baltimore). 2018;97(17):e0526. doi:10.1097/MD.0000000000010526	No results
194	Lieb K, Engelbrecht MA, Gut O, et al. Cognitive impairment in patients with chronic hepatitis treated with interferon alpha (IFNalpha): results from a prospective study. Eur Psychiatry. 2006;21(3):204-210. doi:10.1016/j.eurpsy.2004.09.030	Information of HBV status of cohort not clear
195	Lins L, Aguiar I, Carvalho FM, et al. Oral Health and Quality of Life in Candidates for Liver Transplantation. Transplant Proc. 2017;49(4):836-840. doi:10.1016/j.transproceed.2017.01.049	Information of HBV status of cohort not clear
197	Lok AS, van Leeuwen DJ, Thomas HC, Sherlock S. Psychosocial impact of chronic infection with hepatitis B virus on British patients. Genitourin Med. 1985;61(4):279-282. doi:10.1136/sti.61.4.279	No HRQOL scale used
198	Ma BO, Shim SG, Yang HJ. Association of erectile dysfunction with depression in patients with chronic viral hepatitis. World J Gastroenterol. 2015;21(18):5641-5646. doi:10.3748/wjg.v21.i18.5641	No HRQOL scale used
199	Ma MX, Huang Y, Adams LA, et al. Prevalence of restless legs syndrome in a tertiary cohort of ambulant patients with chronic liver disease. Intern Med J. 2018;48(3):347-350. doi:10.1111/imj.13719	No HRQOL scale used
200	Mabrouk M, Esmat G, Yosry A, et al. Health-related quality of life in Egyptian patients after liver transplantation. Ann Hepatol. 2012;11(6):882-890.	Information of HBV status of cohort not clear
201	Macdonald S, Jepsen P, Alrubaiy L, Watson H, Vilstrup H, Jalan R. Quality of life measures predict mortality in patients with cirrhosis and severe ascites. Aliment Pharmacol Ther. 2019;49(3):321-330. doi:10.1111/apt.15084	Information of HBV status of cohort not clear
202	Macías-Rodríguez RU, Ruiz-Margáin A, Román-Calleja BM, et al. Effect of non-alcoholic beer, diet and exercise on endothelial function, nutrition and quality of life in patients with cirrhosis. World J Hepatol. 2020;12(12):1299-1313. doi:10.4254/wjh.v12.i12.1299	Information of HBV status of cohort not clear
203	Maharshi S, Sharma BC, Sachdeva S, Srivastava S, Sharma P. Efficacy of Nutritional Therapy for Patients With Cirrhosis and Minimal Hepatic Encephalopathy in a Randomized Trial. Clin Gastroenterol Hepatol. 2016;14(3):454-e33. doi:10.1016/j.cgh.2015.09.028	Information of HBV status of cohort not clear
204	Mahmoudi H, Jafari P, Alizadeh-Naini M, Gholami S, Malek-Hosseini SA, Ghaffaripour S. Validity and reliability of Persian version of Chronic Liver Disease Questionnaire (CLDQ). Qual Life Res. 2012;21(8):1479-1485. doi:10.1007/s11136-011-0059-5	Information of HBV status of cohort not clear
205	Mahmoudi H, Jafari P, Ghaffaripour S. Validation of the Persian version of COOP/WONCA functional health status charts in liver transplant candidates. Prog Transplant. 2014;24(2):126-131. doi:10.7182/pit2014491	Information of HBV status of cohort not clear

206	Majstorović B, Janković S, Dimoski Z, Kekuš D, Kocić S, Mijailović Ž. Assessment of the Reliability of the Serbian Version of the Sickness Impact Profile Questionnaire in Patients with Chronic Viral Hepatitis. Srp Arh Celok Lek. 2015;143(11-12):688-694. doi:10.2298/sarh1512688m	Information of HBV status of cohort not clear
207	Malaguarnera G, Pennisi M, Bertino G, et al. Resveratrol in Patients with Minimal Hepatic Encephalopathy. Nutrients. 2018;10(3):329. Published 2018 Mar 9. doi:10.3390/nu10030329	Information of HBV status of cohort not clear
208	Malaguarnera M, Bella R, Vacante M, et al. Acetyl-L-carnitine reduces depression and improves quality of life in patients with minimal hepatic encephalopathy. Scand J Gastroenterol. 2011;46(6):750-759. doi:10.3109/00365521.2011.565067	Information of HBV status of cohort not clear
209	Malaguarnera M, Vacante M, Giordano M, et al. Oral acetyl-L-carnitine therapy reduces fatigue in overt hepatic encephalopathy: a randomized, double-blind, placebo-controlled study. Am J Clin Nutr. 2011;93(4):799-808. doi:10.3945/ajcn.110.007393	No HRQOL scale used
210	Malaguarnera M. Acetyl-L-carnitine in hepatic encephalopathy. Metab Brain Dis. 2013;28(2):193-199. doi:10.1007/s11011-013-9376-4	Review
211	Malik M, Mushtaq H, Hussain A. Health-related Quality of Life and Depression among patients of liver cirrhosis in Pakistan. International Journal of Current Pharmaceutical Research. 2021;13(2). doi:10.22159/ijcpr.2021v13i2.41546	Information of HBV status of cohort not clear
212	Malik P, Kohl C, Holzner B, et al. Distress in primary caregivers and patients listed for liver transplantation. Psychiatry Res. 2014;215(1):159-162. doi:10.1016/j.psychres.2013.08.046	Information of HBV status of cohort not clear
213	Mantas D, Karounis C, Antoniou E, et al. Complications and long-term quality of life following liver transplantation and major gastrointestinal surgery. Arch Hellen Med. 2017;34(2):221-228	Information of HBV status of cohort not clear
214	Marcellin P, Lau GK, Zeuzem S, et al. Comparing the safety, tolerability and quality of life in patients with chronic hepatitis B vs chronic hepatitis C treated with peginterferon alpha-2a. Liver Int. 2008;28(4):477-485. doi:10.1111/j.1478-3231.2008.01696.x	Used HRQOL scale without composite utility value (SF-36)
215	Marchesini G, Bianchi G, Amodio P, et al. Factors associated with poor health-related quality of life of patients with cirrhosis. Gastroenterology. 2001;120(1):170-178. doi:10.1053/gast.2001.21193	Information of HBV status of cohort not clear
216	Martin-Rodriguez A, Perez-San-Gregorio MA, Dominguez-Cabello E, et al. Biopsychosocial functioning among cirrhotic patients in various stages of transplant process in comparison to liver transplant recipients. Anales de psicologia. 2014;30(1):83-92. doi:10.6018/anapesps.30.1.148241	Information of HBV status of cohort not clear

217	Matthew RW, Pasha JA, Meydilin SG, et al. Correlation between hand grip strength and health-related quality of life in liver cirrhosis. Hepatology International. 2020;14(S1):S410. doi:10.1007/s12072-020-10030-4	Utility scores not reported
218	McLean KA, Drake TM, Sgrò A, et al. The effect of liver transplantation on patient-centred outcomes: a propensity-score matched analysis. Transpl Int. 2019;32(8):808-819. doi:10.1111/tri.13416	Information of HBV status of cohort not clear
219	McPhail SM, Amarasena S, Stuart KA, et al. Assessment of health-related quality of life and health utilities in Australian patients with cirrhosis. JGH Open. 2020;5(1):133-142. doi:10.1002/jgh3.12462	Information of HBV status of cohort not clear
220	Meydilin SG, Adji S, Darmawan OJ, et al. Correlation between sleep disturbance and quality of life in liver cirrhosis. Hepatology International. 2020;14(S1):S411. doi:10.1007/s12072-020-10030-4	Utility scores not reported
221	Mikoshiba N, Miyashita M, Sakai T, Tateishi R, Koike K. Depressive symptoms after treatment in hepatocellular carcinoma survivors: prevalence, determinants, and impact on health-related quality of life. Psychooncology. 2013;22(10):2347-2353. doi:10.1002/pon.3300	Information of HBV status of cohort not clear
222	Miotto EC, Campanholo KR, Machado MA, et al. Cognitive performance and mood in patients on the waiting list for liver transplantation and their relation to the model for end-stage liver disease. Arq Neuropsiquiatr. 2010;68(1):62-66. doi:10.1590/s0004-282x2010000100014	Information of HBV status of cohort not clear
223	Mirabdolhagh Hazaveh M, Dormohammadi Toosi T, Nasiri Toosi M, Tavakoli A, Shahbazi F. Prevalence and severity of depression in chronic viral hepatitis in Iran. Gastroenterol Rep (Oxf). 2015;3(3):234-237. doi:10.1093/gastro/gou091	No HRQOL scale used
224	Miranda-Pettersen K, Morais-de-Jesus M, Daltro-Oliveira R, et al. The fatigue impact scale for daily use in patients with hepatitis B virus and hepatitis C virus chronic infections. Ann Hepatol. 2015;14(3):310-316.	Information of HBV status of cohort not clear
225	Mittal VV, Sharma BC, Sharma P, Sarin SK. A randomized controlled trial comparing lactulose, probiotics, and L-ornithine L-aspartate in treatment of minimal hepatic encephalopathy. Eur J Gastroenterol Hepatol. 2011;23(8):725-732. doi:10.1097/MEG.0b013e32834696f5	Information of HBV status of cohort not clear
226	Mohammad S, Kaurs E, Aguirre VP, Varni JW, Alonso EM. Health-related Quality of Life in Infants With Chronic Liver Disease. J Pediatr Gastroenterol Nutr. 2016;62(5):751-756. doi:10.1097/MPG.0000000000001054	Information of HBV status of cohort not clear
227	Monsky WL, Yoneda KY, MacMillan J, et al. Peritoneal and pleural ports for management of refractory ascites and pleural effusions: assessment of impact on patient quality of life and hospice/home nursing care. J Palliat Med. 2009;12(9):811-817. doi:10.1089/jpm.2009.0061	Information of HBV status of cohort not clear

228	Montagnese S, Amato E, Schiff S, et al. A patients' and caregivers' perspective on hepatic encephalopathy. Metab Brain Dis. 2012;27(4):567-572. doi:10.1007/s11011-012-9325-7	Information of HBV status of cohort not clear
229	Montagnese S, Middleton B, Skene DJ, Morgan MY. Night-time sleep disturbance does not correlate with neuropsychiatric impairment in patients with cirrhosis. Liver Int. 2009;29(9):1372-1382. doi:10.1111/j.1478-3231.2009.02089.x	Information of HBV status of cohort not clear
230	Mooney S, Hasssanein TI, Hilsabeck RC, et al. Utility of the Repeatable Battery for the Assessment of Neuropsychological Status (RBANS) in patients with end-stage liver disease awaiting liver transplant. Arch Clin Neuropsychol. 2007;22(2):175-186. doi:10.1016/j.acn.2006.12.005	No HRQOL scale used
231	Moore KA, Burrows GD, Hardy KJ. Anxiety in Chronic Liver Disease: Changes Post Transplantation. Stress Medicine. 1999;13(1):49-57. doi:10.1002/(SICI)1099-1700(199701)13:1<49::AID-SMI720>3.0.CO;2-5	Information of HBV status of cohort not clear
232	Moore KA, McL Jones R, Burrows GD. Quality of life and cognitive function of liver transplant patients: a prospective study. Liver Transpl. 2000;6(5):633-642. doi:10.1053/jlts.2000.9743	Information of HBV status of cohort not clear
233	Moretti R, Caruso P, Tecchiolli M, Gazzin S, Tiribelli C. Management of restless legs syndrome in chronic liver disease: A challenge for the correct diagnosis and therapy. World J Hepatol. 2018;10(3):379-387. doi:10.4254/wjh.v10.i3.379	No HRQOL scale used
234	Moscucci F, Nardelli S, Pentassuglio I, et al. Previous overt hepatic encephalopathy rather than minimal hepatic encephalopathy impairs health-related quality of life in cirrhotic patients. Liver Int. 2011;31(10):1505-1510. doi:10.1111/j.1478-3231.2011.02598.x	Information of HBV status of cohort not clear
235	Mucci S, de Albuquerque Citero V, Gonzalez AM, et al. Validation of the Brazilian version of Chronic Liver Disease Questionnaire. Qual Life Res. 2013;22(1):167-172. doi:10.1007/s11136-012-0138-2	Information of HBV status of cohort not clear
236	Nabi E, Thacker LR, Wade JB, et al. Diagnosis of covert hepatic encephalopathy without specialized tests. Clin Gastroenterol Hepatol. 2014;12(8):1384-1389.e2. doi:10.1016/j.cgh.2013.12.020	Information of HBV status of cohort not clear
237	Nabors LA, Hoffman AR, Ritchey PN. Four illnesses: lenses for critical topics in child quality of life research. J Pediatr. 2011;158(4):529-531. doi:10.1016/j.jpeds.2010.11.057	Review
238	Nagel M, Labenz C, Wörns MA, et al. Impact of acute-on-chronic liver failure and decompensated liver cirrhosis on psychosocial burden and quality of life of patients and their close relatives. Health Qual Life Outcomes. 2020;18(1):10. Published 2020 Jan 13. doi:10.1186/s12955-019-1268-9	Information of HBV status of cohort not clear

239	Nahaz N, Devadas K, Shanavas N, et al. Association of sarcopenia with health related quality of life in cirrhotics. Journal of Gastroenterology and Hepatology. 2019;34(S3):710. doi:10.1111/jgh.14865	Utility scores not reported
240	Nardelli S, Pentassuglio I, Pasquale C, et al. Depression, anxiety and alexithymia symptoms are major determinants of health related quality of life (HRQoL) in cirrhotic patients. Metab Brain Dis. 2013;28(2):239-243. doi:10.1007/s11011-012-9364-0	Information of HBV status of cohort not clear
241	Nazarian GK, Ferral H, Bjarnason H, et al. Effect of transjugular intrahepatic portosystemic shunt on quality of life. AJR Am J Roentgenol. 1996;167(4):963-969. doi:10.2214/ajr.167.4.8819395	Information of HBV status of cohort not clear
242	Neijenhuis M, Gevers TJG, Atwell TD, et al. Development and Validation of a Patient-Reported Outcome Measurement for Symptom Assessment in Cirrhotic Ascites. Am J Gastroenterol. 2018;113(4):567-575. doi:10.1038/ajg.2018.18	Information of HBV status of cohort not clear
243	Nishikawa H, Enomoto H, Yoh K, et al. Health-Related Quality of Life in Chronic Liver Diseases: A Strong Impact of Hand Grip Strength. J Clin Med. 2018;7(12):553. Published 2018 Dec 15. doi:10.3390/jcm7120553	Information of HBV status of cohort not clear
244	Nishikawa H, Enomoto H, Yoh K, et al. Serum zinc concentration and quality of life in chronic liver diseases. Medicine (Baltimore). 2020;99(1):e18632. doi:10.1097/MD.0000000000018632	Information of HBV status of cohort not clear
245	Nishikawa H, Yoh K, Enomoto H, et al. Health-Related Quality of Life and Frailty in Chronic Liver Diseases. Life (Basel). 2020;10(5):76. doi:10.3390/life10050076	Information of HBV status of cohort not clear
246	Nogueira IR, Coelho JCU, Domingos MF, et al. GOOD QUALITY OF LIFE AFTER MORE THAN A DECADE OF LIVING DONOR LIVER TRANSPLANTATION. Arq Gastroenterol. 2021;58(1):10-16. doi:10.1590/S0004-2803.202100000-04	Information of HBV status of cohort not clear
247	O'Carroll RE, Couston M, Cossar J, Masterton G, Hayes PC. Psychological outcome and quality of life following liver transplantation: a prospective, national, single-center study. Liver Transpl. 2003;9(7):712-720. doi:10.1053/jlts.2003.50138	Information of HBV status of cohort not clear
248	Obradovic M, Gluvic Z, Petrovic N, et al. A quality of life assessment and the correlation between generic and disease-specific questionnaires scores in outpatients with chronic liver disease-pilot study. Rom J Intern Med. 2017;55(3):129-137. doi:10.1515/rjim-2017-0014	Information of HBV status of cohort not clear
249	Ock M, Lim SY, Lee HJ, Kim SH, Jo MW. Estimation of utility weights for major liver diseases according to disease severity in Korea. BMC Gastroenterol. 2017;17(1):103. Published 2017 Sep 5. doi:10.1186/s12876-017-0660-3	HRQOL not directly measured from patients

250	Ohashi K, Ishikawa T, Imai M, et al. Relationship between pre-sarcopenia and quality of life in patients with chronic liver disease: a cross-sectional study. Eur J Gastroenterol Hepatol. 2019;31(11):1408-1413. doi:10.1097/MEG.0000000000001415	Information of HBV status of cohort not clear
251	Okushin H, Ohnishi T, Morii K, Uesaka K, Yuasa S. Short-term intravenous interferon therapy for chronic hepatitis B. World J Gastroenterol. 2008;14(19):3038-3043. doi:10.3748/wjg.14.3038	No HRQOL scale used
252	Ong JP, Oehler G, Krüger-Jansen C, Lambert-Baumann J, Younossi ZM. Oral L-ornithine-L-aspartate improves health-related quality of life in cirrhotic patients with hepatic encephalopathy: an open-label, prospective, multicentre observational study. Clin Drug Investig. 2011;31(4):213-220. doi:10.2165/11586700-000000000-00000	Information of HBV status of cohort not clear
253	Ong SC, Lim SG, Li SC. Cultural adaptation and validation of a questionnaire for use in hepatitis B patients. J Viral Hepat. 2009;16(4):272-278. doi:10.1111/j.1365-2893.2009.01073.x	Utility scores not reported
254	Ong SC, Lim SG, Li SC. Reliability and validity of a Chinese version's health-related quality of life questionnaire for hepatitis B patients. Value Health. 2010;13(2):324-327. doi:10.1111/j.1524-4733.2009.00615.x	Utility scores not reported
255	Ordin YS, Dicle A, Wellard S. Quality of life in recipients before and after liver transplantation in Turkey. Prog Transplant. 2011;21(3):260-267. doi:10.7182/prtr.21.3.pl837214k0276260	Information of HBV status of cohort not clear
256	Ortega T, Deulofeu R, Salamero P, et al. Impact of health related quality of life in Catalonia liver transplant patients. Transplant Proc. 2009;41(6):2187-2188. doi:10.1016/j.transproceed.2009.06.139	Information of HBV status of cohort not clear
257	Owens DK, Cardinalli AB, Nease RF Jr. Physicians' assessments of the utility of health states associated with human immunodeficiency virus (HIV) and hepatitis B virus (HBV) infection. Qual Life Res. 1997;6(1):77-86. doi:10.1023/a:1026473613487	HRQOL not directly measured from patients
258	Ozkan M, Corapçioglu A, Balcioglu I, et al. Psychiatric morbidity and its effect on the quality of life of patients with chronic hepatitis B and hepatitis C. Int J Psychiatry Med. 2006;36(3):283-297. doi:10.2190/D37Y-X0JY-39MJ-PVXQ	Utility scores not reported
259	Palmieri VO, Santovito D, Margari F, et al. Psychopathological profile and health-related quality of life (HRQOL) in patients with hepatocellular carcinoma (HCC) and cirrhosis. Clin Exp Med. 2015;15(1):65-72. doi:10.1007/s10238-013-0267-0	Information of HBV status of cohort not clear
260	Pan Y, Barnhart HX. Methods for assessing the reliability of quality of life based on SF-36. Stat Med. 2016;35(30):5656-5665. doi:10.1002/sim.7085	Utility scores not reported
261	Pappa E, Hatzi F, Papadopoulos AA, Niakas D. Psychometrics of the Greek Chronic Liver Disease Questionnaire for Measuring HRQL. Gastroenterol Res Pract. 2015;2015:395951. doi:10.1155/2015/395951	Information of HBV status of cohort not clear

262	Park SJ, Ahn S, Woo SJ, Park KH. Extent of Exacerbation of Chronic Health Conditions by Visual Impairment in Terms of Health-Related Quality of Life. JAMA Ophthalmol. 2015;133(11):1267-1275. doi:10.1001/jamaophthalmol.2015.3055	Information of HBV status of cohort not clear
263	Parkash O, Iqbal R, Jafri F, Azam I, Jafri W. Frequency of poor quality of life and predictors of health related quality of life in cirrhosis at a tertiary care hospital Pakistan. BMC Res Notes. 2012;5:446. Published 2012 Aug 20. doi:10.1186/1756-0500-5-446	Information of HBV status of cohort not clear
264	Patel AV, Wade JB, Thacker LR, et al. Cognitive reserve is a determinant of health-related quality of life in patients with cirrhosis, independent of covert hepatic encephalopathy and model for end-stage liver disease score. Clin Gastroenterol Hepatol. 2015;13(5):987-991. doi:10.1016/j.cgh.2014.09.049	Information of HBV status of cohort not clear
265	Paternostro R, Heinisch BB, Reiberger T, et al. Erectile dysfunction in cirrhosis is impacted by liver dysfunction, portal hypertension, diabetes and arterial hypertension. Liver Int. 2018;38(8):1427-1436. doi:10.1111/liv.13704	No HRQOL scale used
266	Paulson D, Shah M, Miller-Matero LR, Eshelman A, Abouljoud M. Cognition Predicts Quality of Life Among Patients With End-Stage Liver Disease. Psychosomatics. 2016;57(5):514-521. doi:10.1016/j.psym.2016.03.006	Information of HBV status of cohort not clear
267	Pérez-San-Gregorio MÁ, Martín-Rodríguez A, Domínguez-Cabello E, Fernández-Jiménez E, Bernardos-Rodríguez Á. Quality of life and mental health comparisons among liver transplant recipients and cirrhotic patients with different self-perceptions of health. J Clin Psychol Med Settings. 2013;20(1):97-106. doi:10.1007/s10880-012-9309-0	Information of HBV status of cohort not clear
268	Pérez-San-Gregorio MA, Martín-Rodríguez A, Pérez-Bernal J, Maldonado MD. Quality of life in spanish patients with liver transplant. Clin Pract Epidemiol Ment Health. 2010;6:79-85. Published 2010 Aug 27. doi:10.2174/1745017901006010079	Information of HBV status of cohort not clear
269	Pieber K, Crevenna R, Nuhr MJ, et al. Aerobic capacity, muscle strength and health-related quality of life before and after orthotopic liver transplantation: preliminary data of an Austrian transplantation centre. J Rehabil Med. 2006;38(5):322-328. doi:10.1080/16501970600680288	Information of HBV status of cohort not clear
270	Pojoga C, Dumitrascu DL, Pascu O, et al. Impaired health-relatede quality of life in Romanian patients with chronic viral hepatitis before antiviral therapy. European Journal of Gastroenterology & Hepatology. 2004;16:27-31. doi:10.1097/01.meg.0000085522.79233.f9	Utility scores not reported
271	Poo JL, Góngora J, Sánchez-Avila F, et al. Efficacy of oral L-ornithine-L-aspartate in cirrhotic patients with hyperammonemic hepatic encephalopathy. Results of a randomized, lactulose-controlled study. Ann Hepatol. 2006;5(4):281-288.	No HRQOL scale used

272	Poonja Z, Brisebois A, van Zanten SV, Tandon P, Meeberg G, Karvellas CJ. Patients with cirrhosis and denied liver transplants rarely receive adequate palliative care or appropriate management. Clin Gastroenterol Hepatol. 2014;12(4):692-698. doi:10.1016/j.cgh.2013.08.027	No HRQOL scale used
273	Popovic D, Tepavcevic D, Kovacevic N, et al. Quality of life in patients with chronic liver disease. Vojnosanitetski pregled. 2018;75(5):453-460. doi:10.2298/VSP160616339P	Information of HBV status of cohort not clear
274	Popović DDj, Ćulafić DM, Tepavčević DB, et al. Assessment of depression and anxiety in patients with chronic liver disease. Vojnosanit Pregl. 2015;72(5):414-420. doi:10.2298/vsp130904007p	Information of HBV status of cohort not clear
275	Popovic DDj, Kovacevic NV, Kisic Tepavcevic DB, et al. Validation of the chronic liver disease questionnaire in Serbian patients. World J Gastroenterol. 2013;19(30):4950-4957. doi:10.3748/wjg.v19.i30.4950	Information of HBV status of cohort not clear
276	Prasad S, Dhiman RK, Duseja A, Chawla YK, Sharma A, Agarwal R. Lactulose improves cognitive functions and health-related quality of life in patients with cirrhosis who have minimal hepatic encephalopathy. Hepatology. 2007;45(3):549-559. doi:10.1002/hep.21533	Information of HBV status of cohort not clear
277	Premkumar M, Devurgowda D, Vyas T, et al. Left Ventricular Diastolic Dysfunction is Associated with Renal Dysfunction, Poor Survival and Low Health Related Quality of Life in Cirrhosis. J Clin Exp Hepatol. 2019;9(3):324-333. doi:10.1016/j.jceh.2018.08.008	Information of HBV status of cohort not clear
278	Qiao CX, Zhai XF, Ling CQ, et al. Health-related quality of life evaluated by tumor node metastasis staging system in patients with hepatocellular carcinoma. World J Gastroenterol. 2012;18(21):2689-2694. doi:10.3748/wjg.v18.i21.2689	Information of HBV status of cohort not clear
279	Qureshi MO, Khokhar N, Shafqat F. Severity of depression in hepatitis B and hepatitis C patients. J Coll Physicians Surg Pak. 2012;22(10):632-634. doi:10.2012/JCPSP.632634	No HRQOL scale used
280	Ranawaka CK, Miththinda JK, Senanayake SM, et al. Validation of the Sinhala version of the Chronic Liver Disease Questionnaire (CLDQ) for assessment of health related quality of life among Sri Lankan cirrhotics. Ceylon Med J. 2013;58(4):156-162. doi:10.4038/cmj.v58i3.6103	Information of HBV status of cohort not clear
281	Ratcliffe J, Young T, Longworth L, Buxton M. An assessment of the impact of informative dropout and nonresponse in measuring health-related quality of life using the EuroQol (EQ-5D) descriptive system. Value Health. 2005;8(1):53-58. doi:10.1111/j.1524-4733.2005.03068.x	Information of HBV status of cohort not clear
282	Ray I, Dutta D, Basu P, De BK. Quality of life assessment of patients with chronic liver disease in eastern India using a Bengali translation chronic liver disease questionnaire. Indian J Gastroenterol. 2010;29(5):187-195. doi:10.1007/s12664-010-0036-x	Information of HBV status of cohort not clear

283	Rodrigue JR, Nelson DR, Reed AI, Hanto DW, Curry M. Fatigue and sleep quality before and after liver transplantation. Prog Transplant. 2010;20(3):221-233. doi:10.7182/prtr.20.3.x82q1832184j4733	Information of HBV status of cohort not clear
284	Rodrigue JR, Nelson DR, Reed AI, Hanto DW, Curry MP. Is Model for End-Stage Liver Disease score associated with quality of life after liver transplantation?. Prog Transplant. 2011;21(3):207-214. doi:10.7182/prtr.21.3.c508417x010g552n	Information of HBV status of cohort not clear
285	Rostami Z, Lessan Pezeshki M, Soleimani Najaf Abadi A, Einollahi B. Health related quality of life in Iranian hemodialysis patients with viral hepatitis: changing epidemiology. Hepat Mon. 2013;13(6):e9611. Published 2013 May 30. doi:10.5812/hepatmon.9611	Used HRQOL scale without composite utility value (SF-36)
286	Ruppert K, Kuo S, DiMartini A, Balan V. In a 12-year study, sustainability of quality of life benefits after liver transplantation varies with pretransplantation diagnosis. Gastroenterology. 2010;139(5):1619-1629.e16294. doi:10.1053/j.gastro.2010.06.043	Information of HBV status of cohort not clear
287	Russell RT, Feurer ID, Wisawatapnimit P, Lillie ES, Castaldo ET, Pinson CW. Profile of health-related quality of life outcomes after liver transplantation: univariate effects and multivariate models. HPB (Oxford). 2008;10(1):30-37. doi:10.1080/13651820701883106	Information of HBV status of cohort not clear
288	Russell RT, Feurer ID, Wisawatapnimit P, Salomon RM, Pinson CW. The effects of physical quality of life, time, and gender on change in symptoms of anxiety and depression after liver transplantation. J Gastrointest Surg. 2008;12(1):138-144. doi:10.1007/s11605-007-0382-x	Information of HBV status of cohort not clear
289	Saab S, Bownik H, Ayoub N, et al. Differences in health-related quality of life scores after orthotopic liver transplantation with respect to selected socioeconomic factors. Liver Transpl. 2011;17(5):580-590. doi:10.1002/lt.22268	Information of HBV status of cohort not clear
290	Saab S, Ibrahim AB, Shpaner A, et al. MELD fails to measure quality of life in liver transplant candidates. Liver Transpl. 2005;11(2):218-223. doi:10.1002/lt.20345	Information of HBV status of cohort not clear
291	Saab S, Ibrahim AB, Surti B, et al. Pretransplant variables associated with quality of life in liver transplant recipients. Liver Int. 2008;28(8):1087-1094. doi:10.1111/j.1478-3231.2008.01831.x	Information of HBV status of cohort not clear
292	Saffari M, Alavian SM, Naderi MK, Pakpour AH, Al Zaben F, Koenig HG. Cross-Cultural Adaptation and Psychometric Assessment of the Liver Disease Symptom Index 2.0 to Measure Health-Related Quality of Life Among Iranian Patients With Chronic Hepatitis B. J Transcult Nurs. 2016;27(5):496-508. doi:10.1177/1043659615577698	Utility scores not reported
293	Saffari M, Naderi MK, Piper CN, Koenig HG. Multidimensional Fatigue Inventory in People With Hepatitis B Infection: Cross-cultural Adaptation and Psychometric Evaluation of the Persian Version. Gastroenterol Nurs. 2017;40(5):380-392. doi:10.1097/SGA.0000000000000250	Utility scores not reported

294	Salam M, Matherly S, Farooq IS, et al. Modified-orientation log to assess hepatic encephalopathy. Aliment Pharmacol Ther. 2012;35(8):913-920. doi:10.1111/j.1365-2036.2012.05038.x	No HRQOL scale used
295	Salama H, Zekri AR, Ahmed R, et al. Assessment of health-related quality of life in patients receiving stem cell therapy for end-stage liver disease: an Egyptian study. Stem Cell Res Ther. 2012;3(6):49. Published 2012 Dec 3. doi:10.1186/scrt140	No HBV patients
296	Salcedo J, Viray T, Feun L, et al. Health-related quality of life decreases with disease progression from chronic liver disease to cirrhosis and hepatocellular carcinoma. American Journal of Gastroenterology. 2019;114(S):S613-S614. doi:10.14309/01.ajg.0000593864.74956.f8	Information of HBV status of cohort not clear
297	Samanta J, Dhiman RK, Khatri A, et al. Correlation between degree and quality of sleep disturbance and the level of neuropsychiatric impairment in patients with liver cirrhosis. Metab Brain Dis. 2013;28(2):249-259. doi:10.1007/s11011-013-9393-3	Information of HBV status of cohort not clear
298	Santos Junior R, Miyazaki MC, Domingos NA, Valério NI, Silva RF, Silva RC. Patients undergoing liver transplantation: psychosocial characteristics, depressive symptoms, and quality of life. Transplant Proc. 2008;40(3):802-804. doi:10.1016/j.transproceed.2008.02.059	Information of HBV status of cohort not clear
299	Sanyal A, Younossi ZM, Bass NM, et al. Randomised clinical trial: rifaximin improves health-related quality of life in cirrhotic patients with hepatic encephalopathy - a double-blind placebo-controlled study. Aliment Pharmacol Ther. 2011;34(8):853-861. doi:10.1111/j.1365-2036.2011.04808.x	Information of HBV status of cohort not clear
300	Saracino RM, Jutagir DR, Cunningham A, et al. Psychiatric Comorbidity, Health-Related Quality of Life, and Mental Health Service Utilization Among Patients Awaiting Liver Transplant. J Pain Symptom Manage. 2018;56(1):44-52. doi:10.1016/j.jpainsymman.2018.03.001	Information of HBV status of cohort not clear
301	Saroso Olivia JDA, Law Natasha K, Heriyanto Rivaldo S, Sanjaya Gisela M, Patricia A, Andree K, Prasetya Ignatius B. Correlation between skeletal muscle mass index and healthrelated quality of life in liver cirrhosis. Hepatology International. 2020;14(S1):S411. doi:10.1007/s12072-020-10030-4	Utility scores not reported
302	Schomerus H, Hamster W. Quality of life in cirrhotics with minimal hepatic encephalopathy. Metab Brain Dis. 2001;16(1-2):37-41. doi:10.1023/a:1011610427843	Information of HBV status of cohort not clear
304	Scott LJ. Rifaximin: a review of its use in reducing recurrence of overt hepatic encephalopathy episodes. Drugs. 2014;74(18):2153-2160. doi:10.1007/s40265-014-0300-y	Review

305	Sertoz OO, Tuncel OK, Tasbakan MI, et al. Depression and anxiety disorders during pegylated interferon treatment in patients with chronic hepatitis B. Psychiatry and Clinical Psychopharmacology. 2017;27(1), 47-53. doi: 10.1080/24750573.2017.1293251	No HRQOL scale used
306	Shamsaeefar A, Nikeghbalian S, Kazemi K, et al. Quality of life among liver transplantation recipients before and after surgery: A single-center longitudinal study. Indian Journal of Transplantation. 2020;14(1):48-52. doi:10.4103/ijot.ijot_42_19	Information of HBV status of cohort not clear
307	Sharif F, Mohebbi S, Tabatabaee HR, Saberi-Firoozi M, Gholamzadeh S. Effects of psycho-educational intervention on health-related quality of life (QOL) of patients with chronic liver disease referring to Shiraz University of Medical Sciences. Health Qual Life Outcomes. 2005;3:81. Published 2005 Dec 16. doi:10.1186/1477-7525-3-81	Information of HBV status of cohort not clear
308	Shimakawa Y, Takao Y, Anderson ST, et al. The prevalence and burden of symptoms in patients with chronic liver diseases in The Gambia, West Africa. Palliat Med. 2015;29(2):184-185. doi:10.1177/0269216314547103	No HRQOL scale used
309	Sidhu SS, Goyal O, Mishra BP, Sood A, Chhina RS, Soni RK. Rifaximin improves psychometric performance and health-related quality of life in patients with minimal hepatic encephalopathy (the RIME Trial). Am J Gastroenterol. 2011;106(2):307-316. doi:10.1038/ajg.2010.455	Information of HBV status of cohort not clear
310	Sidhu SS, Goyal O, Parker RA, Kishore H, Sood A. Rifaximin vs. lactulose in treatment of minimal hepatic encephalopathy. Liver Int. 2016;36(3):378-385. doi:10.1111/liv.12921	Information of HBV status of cohort not clear
311	Simsek I, Aslan G, Akarsu M, Koseoglu H, Esen A. Assessment of sexual functions in patients with chronic liver disease. Int J Impot Res. 2005;17(4):343-345. doi:10.1038/sj.ijir.3901316	No HRQOL scale used
312	Singh J, Sharma BC, Puri V, Sachdeva S, Srivastava S. Sleep disturbances in patients of liver cirrhosis with minimal hepatic encephalopathy before and after lactulose therapy. Metab Brain Dis. 2017;32(2):595-605. doi:10.1007/s11011-016-9944-5	No HRQOL scale used
313	Singh N, Gayowski T, Wagener MM, Marino IR. Depression in patients with cirrhosis. Impact on outcome. Dig Dis Sci. 1997;42(7):1421-1427. doi:10.1023/a:1018898106656	No HRQOL scale used
314	Sirivatanauksorn Y, Dumronggittigule W, Limsrichamrern S, et al. Quality of life among liver transplantation patients. Transplant Proc. 2012;44(2):532-538. doi:10.1016/j.transproceed.2011.12.056	Information of HBV status of cohort not clear
315	Skladany L, Vnencakova J, Skvarkova B, Koller T. Frailty assessed by the liver frailty index (LFI) predicts poor health-related quality of life (QOL) in patients hospitalised with liver cirrhosis. Journal of Hepatology. 2020;73(S1):S735. doi:10.1016/S0168-8278	Information of HBV status of cohort not clear

316	Sobhonslidsuk A, Silpakit C, Kongsakon R, Satitpornkul P, Sripetch C, Khanthavit A. Factors influencing health-related quality of life in chronic liver disease. World J Gastroenterol. 2006;12(48):7786-7791. doi:10.3748/wjg.v12.i48.7786	Information of HBV status of cohort not clear
317	Sobhonslidsuk A, Silpakit C, Kongsakon R, Satitpornkul P, Sripetch C. Chronic liver disease questionnaire: translation and validation in Thais. World J Gastroenterol. 2004;10(13):1954-1957. doi:10.3748/wjg.v10.i13.1954	Information of HBV status of cohort not clear
318	Sohn W, Park J, Cho YK, Kim BI. Compliance of the screening for hepatocellular carcinoma in patients with chronic hepatitis B or C. Hepatology International. 2022;16(S1):S399. doi:10.1007/s12072-022-10337-4	No HRQOL scale used
319	Song YN, Sun JJ, Lu YY, et al. Therapeutic efficacy of fuzheng-huayu tablet based traditional chinese medicine syndrome differentiation on hepatitis-B-caused cirrhosis: a multicenter double-blind randomized controlled trail. Evid Based Complement Alternat Med. 2013;2013:709305. doi:10.1155/2013/709305	No HRQOL scale used
320	Soontararatpong R, Pornthisarn B, Vilaichone R, et al. ASSOCIATION BETWEEN FRAILTY STATUS AND QUALITY OF LIFE IN COMPENSATED CIRRHOTIC PATIENT. Gastroenterology. 2020;158(6S1):S-1457. doi:10.1016/S0016-5085	Utility scores not reported
321	Sorrell JH, Zolnikov BJ, Sharma A, Jinnai I. Cognitive impairment in people diagnosed with end-stage liver disease evaluated for liver transplantation. Psychiatry Clin Neurosci. 2006;60(2):174-181. doi:10.1111/j.1440-1819.2006.01483.x	No HBV patients
322	Souza NP, Villar LM, Garbin AJ, Rovida TA, Garbin CA. Assessment of health-related quality of life and related factors in patients with chronic liver disease. Braz J Infect Dis. 2015;19(6):590-595. doi:10.1016/j.bjid.2015.08.003	Information of HBV status of cohort not clear
323	Spiegel BM, Bolus R, Han S, et al. Development and validation of a disease-targeted quality of life instrument in chronic hepatitis B: the hepatitis B quality of life instrument, version 1.0. Hepatology. 2007;46(1):113-121. doi:10.1002/hep.21692	Unique HRQOL tool used (HBQOL)
324	Spina G, Santambrogio R, Opocher E, et al. Improved quality of life after distal splenorenal shunt. A prospective comparison with side-to-side portacaval shunt. Ann Surg. 1988;208(1):104-109. doi:10.1097/00000658-198807000-00015	Information of HBV status of cohort not clear
325	Steel JL, Chopra K, Olek MC, Carr BI. Health-related quality of life: Hepatocellular carcinoma, chronic liver disease, and the general population. Qual Life Res. 2007;16(2):203-215. doi:10.1007/s11136-006-9111-2	Information of HBV status of cohort not clear
326	Stepanova M, De Avila L, Afendy M, et al. Direct and Indirect Economic Burden of Chronic Liver Disease in the United States. Clin Gastroenterol Hepatol. 2017;15(5):759-766.e5. doi:10.1016/j.cgh.2016.07.020	Information of HBV status of cohort not clear

327	Stepanova M, Nader F, Bureau C, et al. Patients with refractory ascites treated with alfapump® system have better health-related quality of life as compared to those treated with large volume paracentesis: the results of a multicenter randomized controlled study. Qual Life Res. 2018;27(6):1513-1520. doi:10.1007/s11136-018-1813-8	Information of HBV status of cohort not clear
328	Stewart KE, Hart RP, Gibson DP, Fisher RA. Illness apprehension, depression, anxiety, and quality of life in liver transplant candidates: implications for psychosocial interventions. Psychosomatics. 2014;55(6):650-658. doi:10.1016/j.psym.2013.10.002	Information of HBV status of cohort not clear
329	Stine JG, Stukenborg GJ, Wang J, et al. Liver transplant candidates have impaired quality of life across health domains as assessed by computerized testing. Ann Hepatol. 2020;19(1):62-68. doi:10.1016/j.aohep.2019.06.018	Information of HBV status of cohort not clear
330	Stotts MJ, Cheung A, Hammami MB, et al. Evaluation of Serum-Derived Bovine Immunoglobulin Protein Isolate in Subjects With Decompensated Cirrhosis With Ascites. Cureus. 2021;13(6):e15403. doi:10.7759/cureus.15403	Information of HBV status of cohort not clear
331	Sumskiene J, Sumskas L, Petrauskas D, Kupcinskas L. Disease-specific health-related quality of life and its determinants in liver cirrhosis patients in Lithuania. World J Gastroenterol. 2006;12(48):7792-7797. doi:10.3748/wjg.v12.i48.7792	Information of HBV status of cohort not clear
332	Susanto JP, Mustika S, Pratomo B. Correlation between quality of life with the severity of liver cirrhosis. Hepatology International. 2020;14(S1):S421. doi:10.1007/s12072-020-10030-4	Information of HBV status of cohort not clear
333	Suzuki K, Suzuki K, Koizumi K, et al. Effect of symptomatic gastroesophageal reflux disease on quality of life of patients with chronic liver disease. Hepatol Res. 2008;38(4):335-339. doi:10.1111/j.1872-034X.2007.00275.x	Information of HBV status of cohort not clear
334	Svirtlih N, Pavic S, Terzic D, et al. Reduced quality of life in patients with chronic viral liver disease as assessed by SF12 questionnaire. J Gastrointestin Liver Dis. 2008;17(4):405-409.	Used HRQOL scale without composite utility value (SF-36)
335	Swanson A, Geller J, DeMartini K, Fernandez A, Fehon D. Active Coping and Perceived Social Support Mediate the Relationship Between Physical Health and Resilience in Liver Transplant Candidates. J Clin Psychol Med Settings. 2018;25(4):485-496. doi:10.1007/s10880-018-9559-6	Information of HBV status of cohort not clear
336	Tan HH, Lee GH, Thia KT, Ng HS, Chow WC, Lui HF. Minimal hepatic encephalopathy runs a fluctuating course: results from a three-year prospective cohort follow-up study. Singapore Med J. 2009;50(3):255-260.	Information of HBV status of cohort not clear

337	Tan HK, Chang PE, Lee Y, et al. Translation and cultural adaption of the Chronic Liver Disease Questionnaire for the Mandarin-speaking Chinese population in Singapore through cognitive debriefing. Proceedings of Singapore Healthcare. 2019;28(1):48-54. doi:10.1177/2010105818782710	Information of HBV status of cohort not clear
338	Tan NC, Cheah SL, Teo EK, Yang LH. Patients with chronic hepatitis B infection: what is their quality of life?. Singapore Med J. 2008;49(9):682-687.	Used HRQOL scale without composite utility value (SF-36, HQLQ)
339	Taneja S, Pathak MN, Rathi S, Duseja A, Chawla YK, Dhiman RK. Extrapyramidal Signs and Health Related Quality of Life in Patients with Cirrhosis. Journal of Clinical and Experimental Hepatology. 2017;7(S1):S22-S83. doi:10.1016/j.jceh.2017.01.094	Utility scores not reported
340	Tanikella R, Kawut SM, Brown RS Jr, et al. Health-related quality of life and survival in liver transplant candidates. Liver Transpl. 2010;16(2):238-245. doi:10.1002/lt.21984	Information of HBV status of cohort not clear
341	Tapper EB, Baki J, Parikh ND, Lok AS. Frailty, Psychoactive Medications, and Cognitive Dysfunction Are Associated With Poor Patient-Reported Outcomes in Cirrhosis. Hepatology. 2019;69(4):1676-1685. doi:10.1002/hep.30336	Information of HBV status of cohort not clear
342	Taru V, Indre MG, Ignat MD, et al. Validation and Performance of Chronic Liver Disease Questionnaire (CLDQ-RO) in the Romanian Population. J Gastrointestin Liver Dis. 2021;30(2):240-246. doi:10.15403/jgld-3405	Information of HBV status of cohort not clear
343	Teixeira HR, Marques DM, Lopes AR, et al. Anxiety and Stress Levels on Liver Transplantation Candidates. Transplant Proc. 2016;48(7):2333-2337. doi:10.1016/j.transproceed.2016.06.031	No HRQOL scale used
344	Thakur J, Rathi S, Grover S, et al. Tadalafil, a Phosphodiesterase-5 Inhibitor, Improves Erectile Dysfunction in Patients With Liver Cirrhosis. J Clin Exp Hepatol. 2019;9(3):312-317. doi:10.1016/j.jceh.2018.07.007	No HRQOL scale used
345	Toda K, Miwa Y, Kuriyama S, et al. Erectile dysfunction in patients with chronic viral liver disease: its relevance to protein malnutrition. J Gastroenterol. 2005;40(9):894-900. doi:10.1007/s00535-005-1634-8	No HRQOL scale used
346	Togashi J, Sugawara Y, Akamatsu N, et al. Quality of life after adult living donor liver transplantation: A longitudinal prospective follow-up study. Hepatol Res. 2013;43(10):1052-1063. doi:10.1111/hepr.12060	Information of HBV status of cohort not clear
347	Tryc AB, Pflugrad H, Goldbecker A, et al. New-onset cognitive dysfunction impairs the quality of life in patients after liver transplantation. Liver Transpl. 2014;20(7):807-814. doi:10.1002/lt.23887	Information of HBV status of cohort not clear
348	Tsai CF, Chu CJ, Wang YP, et al. Increased serum interleukin-6, not minimal hepatic encephalopathy, predicts poor sleep quality in nonalcoholic cirrhotic patients. Aliment Pharmacol Ther. 2016;44(8):836-845. doi:10.1111/apt.13765	No HRQOL scale used

349	Tsushima M, Tsushima W, Tsushima V, et al. Use of ImPACT to diagnose minimal hepatic encephalopathy: an accurate, practical, user-friendly internet-based neuropsychological test battery. Dig Dis Sci. 2013;58(9):2673-2681. doi:10.1007/s10620-013-2668-z	No HRQOL scale used
350	Tufan ZK, Arslan H, Yildiz F, et al. Acupuncture for depression and myalgia in patients with hepatitis: an observational study. Acupunct Med. 2010;28(3):136-139. doi:10.1136/aim.2009.002170	No HRQOL scale used
351	Unal G, de Boer JB, Borsboom GJ, Brouwer JT, Essink-Bot M, de Man RA. A psychometric comparison of health-related quality of life measures in chronic liver disease. J Clin Epidemiol. 2001;54(6):587-596. doi:10.1016/s0895-4356(00)00372-3	Information of HBV status of cohort not clear
352	Urano E, Yamanaka-Okumura H, Teramoto A, et al. Pre- and postoperative nutritional assessment and health-related quality of life in recipients of living donor liver transplantation. Hepatol Res. 2014;44(11):1102-1109. doi:10.1111/hepr.12263	Information of HBV status of cohort not clear
353	van der Plas SM, Hansen BE, de Boer JB, et al. Generic and disease-specific health related quality of life of liver patients with various aetiologies: a survey. Qual Life Res. 2007;16(3):375-388. doi:10.1007/s11136-006-9131-y	Information of HBV status of cohort not clear
354	van der Plas SM, Hansen BE, de Boer JB, et al. The Liver Disease Symptom Index 2.0; validation of a disease-specific questionnaire. Qual Life Res. 2004;13(8):1469-1481. doi:10.1023/B:QURE.0000040797.17449.c0	Information of HBV status of cohort not clear
355	van Hout B, Janssen MF, Feng YS, et al. Interim scoring for the EQ-5D-5L: mapping the EQ-5D-5L to EQ-5D-3L value sets. Value Health. 2012;15(5):708-715. doi:10.1016/j.jval.2012.02.008	Information of HBV status of cohort not clear
356	VanWagner LB, Uttal S, Lapin B, et al. Use of Six-Minute Walk Test to Measure Functional Capacity After Liver Transplantation. Phys Ther. 2016;96(9):1456-1467. doi:10.2522/ptj.20150376	No HBV patients
357	Varakanahalli S, Sharma BC, Srivastava S, Sachdeva S, Dahale AS. Secondary prophylaxis of hepatic encephalopathy in cirrhosis of liver: a double-blind randomized controlled trial of L-ornithine L-aspartate versus placebo. Eur J Gastroenterol Hepatol. 2018;30(8):951-958. doi:10.1097/MEG.0000000000001137	Information of HBV status of cohort not clear
358	Vidyani A, Miftahussurur M, Priyantini D, et al. Quality of life and related factors among hepatitis B virus infected individuals. Systematic Reviews in Pharmacy. 2020;11(3):960-964. doi:10.31838/srp.2020.3.147	Review
359	Wang JY, Zhang NP, Chi BR, et al. Prevalence of minimal hepatic encephalopathy and quality of life evaluations in hospitalized cirrhotic patients in China. World J Gastroenterol. 2013;19(30):4984-4991. doi:10.3748/wjg.v19.i30.4984	Information of HBV status of cohort not clear

360	Weinstein AA, Diao G, Baghi H, Escheik C, Gerber LH, Younossi ZM. Demonstration of two types of fatigue in subjects with chronic liver disease using factor analysis. Qual Life Res. 2017;26(7):1777-1784. doi:10.1007/s11136-017-1516-6	No HBV patients
361	Wells CD, Murrill WB, Arguedas MR. Comparison of health-related quality of life preferences between physicians and cirrhotic patients: implications for cost-utility analyses in chronic liver disease. Dig Dis Sci. 2004;49(3):453-458. doi:10.1023/b:ddas.0000020502.46886.c1	Information of HBV status of cohort not clear
362	Wiesinger GF, Quittan M, Zimmermann K, et al. Physical performance and health-related quality of life in men on a liver transplantation waiting list. J Rehabil Med. 2001;33(6):260-265. doi:10.1080/165019701753236446	Information of HBV status of cohort not clear
363	Wu X, Hong J, Zhou J, et al. Antiviral therapy improves long-term health related quality of life in compensated HBV-cirrhosis patients. Hepatology. 2020;72(1S):453A. doi:10.1002/hep.31579	Unclear how many patients included in utility measures
364	Wunsch E, Koziarska D, Milkiewicz M, et al. In patients with liver cirrhosis, proinflammatory interleukins correlate with health-related quality of life irrespective of minimal hepatic encephalopathy. Eur J Gastroenterol Hepatol. 2013;25(12):1402-1407. doi:10.1097/MEG.0b013e328365a447	Information of HBV status of cohort not clear
365	Wunsch E, Naprawa G, Koziarska D, Milkiewicz M, Nowacki P, Milkiewicz P. Serum natremia affects health-related quality of life in patients with liver cirrhosis: a prospective, single centre study. Ann Hepatol. 2013;12(3):448-455.	Information of HBV status of cohort not clear
366	Wunsch E, Szymanik B, Post M, Marlicz W, Mydłowska M, Milkiewicz P. Minimal hepatic encephalopathy does not impair health-related quality of life in patients with cirrhosis: a prospective study. Liver Int. 2011;31(7):980-984. doi:10.1111/j.1478-3231.2011.02465.x	Information of HBV status of cohort not clear
367	Xiao G, Ye Q, Han T, Yan J, Sun L, Wang F. Study of the sleep quality and psychological state of patients with hepatitis B liver cirrhosis. Hepatol Res. 2018;48(3):E275-E282. doi:10.1111/hepr.12981	No HRQOL scale used
368	Yamanouchi K, Takatsuki M, Hidaka M, et al. Changes in quality of life after hepatectomy and living donor liver transplantation. Hepatogastroenterology. 2012;59(117):1569-1572. doi:10.5754/hge10008	Information of HBV status of cohort not clear
369	Yayan EH, Düken ME. Comparison of Quality of Life and Caregiving Burden of 2- to 4-Year-Old Children Post Liver Transplant and Their Parents. Gastroenterol Nurs. 2020;43(4):310-316. doi:10.1097/SGA.0000000000000448	Information of HBV status of cohort not clear
370	Yeo W, Mo FK, Koh J, et al. Quality of life is predictive of survival in patients with unresectable hepatocellular carcinoma. Ann Oncol. 2006;17(7):1083-1089. doi:10.1093/annonc/mdl065	Information of HBV status of cohort not clear

371	Yilmaz A, Ucmak F, Dönmezdil S, et al. Somatosensory Amplification, Anxiety, and Depression in Patients With Hepatitis B: Impact on Functionality [published correction appears in Medicine (Baltimore). 2016 Jul 18;95(28):e57b2]. Medicine (Baltimore). 2016;95(21):e3779. doi:10.1097/MD.0000000000003779	No HRQOL scale used
372	Yoshimura E, Ichikawa T, Miyaaki H, et al. Screening for minimal hepatic encephalopathy in patients with cirrhosis by cirrhosis-related symptoms and a history of overt hepatic encephalopathy. Biomed Rep. 2016;5(2):193-198. doi:10.3892/br.2016.702	Information of HBV status of cohort not clear
373	Younossi Z, Henry L. Overall health-related quality of life in patients with end-stage liver disease. Clin Liver Dis (Hoboken). 2015;6(1):9-14. Published 2015 Jul 28. doi:10.1002/cld.480	Review
374	Younossi Z, Stepanova M, Younossi I, et al. Long-term follow-up of treated patients with chronic hepatitis b infection: Patient-reported outcomes. Hepatology International. 2019;13(S1):S48-S49. doi:10.1007/s12072-019-09936-5	Abstract
375	Younossi ZM, Boparai N, McCormick M, Price LL, Guyatt G. Assessment of utilities and health-related quality of life in patients with chronic liver disease. Am J Gastroenterol. 2001;96(2):579-583. doi:10.1111/j.1572-0241.2001.03537.x	Information of HBV status of cohort not clear
376	Younossi ZM, Boparai N, Price LL, Kiwi ML, McCormick M, Guyatt G. Health-related quality of life in chronic liver disease: the impact of type and severity of disease. Am J Gastroenterol. 2001;96(7):2199-2205. doi:10.1111/j.1572-0241.2001.03956.x	Information of HBV status of cohort not clear
377	Younossi ZM, Golabi P, Henry L. A Comprehensive Review of Patient-reported Outcomes in Patients With Chronic Liver Diseases. J Clin Gastroenterol. 2019;53(5):331-341. doi:10.1097/MCG.0000000000001179	Review
378	Younossi ZM, McCormick M, Price LL, et al. Impact of liver transplantation on health-related quality of life. Liver Transpl. 2000;6(6):779-783. doi:10.1053/jlts.2000.18499	No HBV patients
379	Younossi ZM, Stepanova M, Younossi I, Racila A. Development and validation of a hepatitis B-specific health-related quality-of-life instrument: CLDQ-HBV. J Viral Hepat. 2021;28(3):484-492. doi:10.1111/jvh.13451	Utility scores not reported
380	Younossi ZM. Patient-Reported Outcomes for Patients With Chronic Liver Disease. Clin Gastroenterol Hepatol. 2018;16(6):793-799. doi:10.1016/j.cgh.2017.12.028	Review
381	Yu J, Yu Y. Therapeutic effects of targeted nursing interventions combined with auricular-plaster therapy on anxiety level and life quality of Hepatitis B patients. Pak J Pharm Sci. 2018;31(4):1375-1378.	Unique HRQOL tool used (with incorrect reference)

382	Zahn A, Seubert L, Jünger J, et al. Factors influencing long-term quality of life and depression in German liver transplant recipients: a single-centre cross-sectional study. Ann Transplant. 2013;18:327-335. Published 2013 Jun 26. doi:10.12659/AOT.883962	Information of HBV status of cohort not clear
383	Zandi M, Adib-Hajbagheri M, Memarian R, Nejhad AK, Alavian SM. Effects of a self-care program on quality of life of cirrhotic patients referring to Tehran Hepatitis Center. Health Qual Life Outcomes. 2005;3:35. Published 2005 May 18. doi:10.1186/1477-7525-3-35	Information of HBV status of cohort not clear
384	Zannella A, Fanella S, Marignani M, Begini P. Quality of life in patients with cirrhosis during the COVID 19 emergency: An Italian awareness survey. United European Gastroenterology Journal. 2020;8(8S):869. doi:10.1177/2050640620927345	Information of HBV status of cohort not clear
385	Zenith L, Meena N, Ramadi A, et al. Eight weeks of exercise training increases aerobic capacity and muscle mass and reduces fatigue in patients with cirrhosis. Clin Gastroenterol Hepatol. 2014;12(11):1920-6.e2. doi:10.1016/j.cgh.2014.04.016	Information of HBV status of cohort not clear
386	Zhang W, Wang LQ, Liu YB. Evaluation on quality of life and analysis on its correlation with TCM syndromes in patients of chronic hepatitis B. Chin J Integr Med. 2009;15(1):30-33. doi:10.1007/s11655-009-0030-z	Utility scores not reported
387	Zhao L, Xu L, Lai Y, Che C, Zhou Y. Temporal changes of smoking status and motivation in Chinese patients with hepatitis B: relationship with anxiety and depression. J Clin Nurs. 2012;21(15-16):2193-2201. doi:10.1111/j.1365-2702.2011.04018.x	No HRQOL scale used
388	Zhou KN, Zhang M, Wu Q, Ji ZH, Zhang XM, Zhuang GH. Psychometrics of chronic liver disease questionnaire in Chinese chronic hepatitis B patients. World J Gastroenterol. 2013;19(22):3494-3501. doi:10.3748/wjg.v19.i22.3494	Utility scores not reported
389	Zhou KN, Zhang M, Wu Q, Ji ZH, Zhang XM, Zhuang GH. Reliability, validity and sensitivity of the Chinese (simple) short form 36 health survey version 2 (SF-36v2) in patients with chronic hepatitis B. J Viral Hepat. 2013;20(4):e47-e55. doi:10.1111/jvh.12030	Utility scores not reported
390	Zhou YQ, Chen SY, Jiang LD, et al. Development and evaluation of the quality of life instrument in chronic liver disease patients with minimal hepatic encephalopathy. J Gastroenterol Hepatol. 2009;24(3):408-415. doi:10.1111/j.1440-1746.2008.05678.x	Information of HBV status of cohort not clear
391	Zhu HP, Gu YR, Zhang GL, et al. Depression in patients with chronic hepatitis B and cirrhosis is closely associated with the severity of liver cirrhosis. Exp Ther Med. 2016;12(1):405-409. doi:10.3892/etm.2016.3271	No HRQOL scale used
392	Zhu L, Chen B, Chen Y, et al. Responsiveness and minimal important changes of the CHBQOL instrument in patients with chronic hepatitis B. Quality of Life Research. 2019;28(S1):S160. doi:10.1007/s11136-019-02257-y	Utility scores not reported

393	Zhu L, Kong J, Zheng Y, et al. Development and initial validation of the chronic hepatitis B quality of life instrument (CHBQOL) among Chinese patients. Qual Life Res. 2019;28(11):3071-3081. doi:10.1007/s11136-019-02240-7	Utility scores not reported
394	Sagara MK, Gangadharan V, Sristhi R, Shaji B, Kellarai A, Joel JJ. Assessment of health-related quality of life and its contributing factors using the WHOQOL-BREF questionnaire in patients with chronic liver disease. J Appl Pharm Sci, 2023; 13(06):246–255. https://doi.org/10.7324/JAPS.2023.120282	Information of HBV status of cohort not clear
395	Natour RT, Midlej A, Mahajna E, Kopelman Y, Abo-Mouch S, Baker FA. Chronic hepatitis B beyond clinical burden: Psychosocial effects and impact on quality of life. J Viral Hepat. 2024 Jan;31(1):12-20. doi: 10.1111/jvh.13894.	Utility scores not reported
396	Tajiri K, Futsukaichi YH, Murayama A, Minemura M, Takahara T, Yasuda I. Chronic liver disease questionnaire to manage patients with chronic liver diseases. Hepatol Res. 2022 Aug;52(8):712-720. doi: 10.1111/hepr.13774.	Utility scores not reported
397	Teshome E, Hailu W, Adane A, Belayneh Melese E, Abebaw Angaw D, Tarekegn GE. Clinical and individual factors of quality of life of chronic liver disease patients at University of Gondar comprehensive specialized hospital, Northwest Ethiopia 2022. Medicine (Baltimore). 2023 Nov 10;102(45):e35425. doi: 10.1097/MD.0000000000035425.	Utility scores not reported
398	Ahmed S, Méndez RY, Naveed S, Akhter S, Mushtaque I, Malik MA, Ahmad W, Figueroa RN, Younas A. Assessment of hepatitis-related knowledge, attitudes, and practices on quality of life with the moderating role of internalized stigma among hepatitis B-positive patients in Pakistan. Health Psychol Behav Med. 2023 Mar 30;11(1):2192782. doi: 10.1080/21642850.2023.2192782.	Utility scores not reported
399	Sirisunhirun P, Bandidniyamanon W, Jrerattakon Y, Muangsomboon K, Pramyothin P, Nimanong S, Tanwandee T, Charatcharoenwitthaya P, Chainuvati S, Chotiyaputta W. Effect of a 12-week home-based exercise training program on aerobic capacity, muscle mass, liver and spleen stiffness, and quality of life in cirrhotic patients: a randomized controlled clinical trial. BMC Gastroenterol. 2022 Feb 14;22(1):66. doi: 10.1186/s12876-022-02147-7.	Utility scores not reported
400	Kiratli K, Dikici O, Kose S. Evaluation of Depression, Anxiety and Health- Related Quality of Life in Patients with Hepatitis B Virus-Infection. 2023; 28(2): 264-274. Doi:10.5578/flora.20239925	Utility scores not reported
401	Nathiya D, Raj P, Singh P, Bareth H, Tejavath AS, Suman S, Tomar BS, Rai RR. Frailty Predicting Health-Related Quality of Life Trajectories in Individuals with Sarcopenia in Liver Cirrhosis: Finding from BCAAS Study. J Clin Med. 2023 Aug 17;12(16):5348. doi: 10.3390/jcm12165348.	Information of HBV status of cohort not clear
402	Onghena L, Berrevoet F, Vanlander A, Van Vlierberghe H, Verhelst X, Hoste E, Poppe C. Illness cognitions and health-related quality of life in liver transplant patients related to length	Information of HBV status of cohort not clear

	of stay, comorbidities and complications. Qual Life Res. 2022 Aug;31(8):2493-2504. doi: 10.1007/s11136-022-03083-5.	
403	Lu M, Rupp LB, Melkonian C, Trudeau S, Daida YG, Schmidt MA, Gordon SC. Persistent pruritus associated with worse quality of life in patients with chronic hepatitis. Liver Int. 2023 Dec 11. doi: 10.1111/liv.15803.	Utility scores not reported
404	Desai AP, Madathanapalli A, Tang Q, Orman ES, Lammert C, Patidar KR, Nephew LD, Ghabril M, Monahan PO, Chalasani N. PROMIS Profile-29 is a valid instrument with distinct advantages over legacy instruments for measuring the quality of life in chronic liver disease. Hepatology. 2023 Dec 1;78(6):1788-1799. doi: 10.1097/HEP.0000000000000480.	No HBV patients
405	Bagheri Lankarani K, Honarvar B, Akbari M, Bozorgnia N, Rabiey Faradonbeh M, Bagherpour M, Nikeghbalian S, Shamsaeefar A, Malekhosseini SA. Quality of Life and Its Determinants in Liver Transplantation Candidates: A Missed Link in Liver Care Program during the Waiting Time for Liver Transplantation. Iran J Med Sci. 2022 May;47(3):227-235. doi: 10.30476/IJMS.2021.88302.1895.	Information of HBV status of cohort not clear
406	Moslemi F, Jaganshahi S, Hashemipour MA. Relationship between Quality of Life and Oral Health Status in Patients with Chronic Liver Disease. PESQUISA BRASILEIRA EM ODONTOPEDIATRIA E CLINICA INTEGRADA 2023;23(): 2023. doi: 10.1590/pboci.2028.080/.	Information of HBV status of cohort not clear
407	Wang T, Kong LN, Yao Y, Li L. Self-Efficacy, Coping Strategies and Quality of Life among Patients with Chronic Hepatitis B. West J Nurs Res. 2023 Sep;45(9):800-806. doi: 10.1177/01939459231184714.	Used HRQOL scale without composite utility value (SF-36)
408	Ibrahim Y, Umstead M, Wang S, Cohen C. The Impact of Living With Chronic Hepatitis B on Quality of Life: Implications for Clinical Management. J Patient Exp. 2023 Nov 13;10:23743735231211069. doi: 10.1177/23743735231211069.	Unique HRQOL tool used
409	Balbinot JC, Souza Aw, Pontarolo R, et al. Validation of the Brazilian version of the hepatitis B quality of life evaluation instrument - HBQOL, and its application to patients with chronic hepatitis B in Cascavel – PR. Brazilian Journal of Pharmaceutical Sciences. 2022;58:e191111. doi: 10.1590/s2175-97902022e191111.	Unique HRQOL tool used
410	Gong X, Chen Z, Zhang X, Zheng Y, Zhang H. Values of Serum HBsAg and HBeAg Levels for Virological Response of Patients with HBV-Related Liver Cirrhosis Treated by Entecavir. Clin Lab. 2023 Jun 1;69(6). doi: 10.7754/Clin.Lab.2022.220913.	Abstract

Abbreviations: HBV, hepatitis B virus; HRQOL, health-related quality of life

Appendix 5: Summary of excluded articles due to unclear stage of disease

First Author and Year	Study Year	Study Design	Study setting	Country	WHO Region	Identify as male (%)	Age (mean)	Non-white race (%)	Treatment details	n	Utility instrument used
Bondini 2007	pre-2007	obs	Database	USA	Americas	65	44.2	84	NA	68	CLDQ, HUI2
Castellanos-Fernandez 2021	2018-2019	obs	Tertiary academic centre	Cuba	Americas	56	45.9	28.6	82.4% on antiviral	91	CLDQ
Drazic 2013	pre-2013	obs	Online survey through advertisements	Australia	Western Pacific	45	39.67	75	30% on antiviral	18	CLDQ
Gutteling 2010	pre-2010	obs	Medical centre	Netherlands	Europe	NA	NA	NA	Exclude IFN past year	55	SF-6D
Kim 2015	2011-2012	obs	Community health survey	South Korea	Western Pacific	55.6	NA	NA	NA	7098	VAS, EQ-5D-3L
Parvizi 2016	2015-2016	obs	Transplant Coordination Office of Hospital	Iran	Eastern Med	NA	NA	NA	Transplant waiting list	32	CLDQ
Scalone 2013	2010-2011	obs	Routine outpt visit or examination 72%, 25% inpt, 3% hospitalised for hepatic condition	Italy	Europe	69.7	52	NA	NA	221	EQ-5D-3L, EQ-5D-5L, VAS
Tanaka 2015	pre-2015	obs	Outpatient clinics of two liver units	Japan	Western Pacific	59.1	64.5	NA	Exclude IFN past 6 months	22	CLDQ
ul Haq 2014	2011	obs	Public hospitals	Pakistan	Eastern Med	59.5	36	NA	NA	390	EQ-5D-3L, VAS
ul Haq 2012	2011	obs	Public hospitals	Pakistan	Eastern Med	59.5	36.07	NA	NA	390	EQ-5D-3L, VAS
Vu 2019	2018	obs	Chronic hepatitis clinic at hospital	Vietnam	Western Pacific	54.5	49.2	NA	NA	298	VAS
Fotos 2018	2014	obs	Outpatient hepatology unit tertiary general hospital	Greece	Europe	55.9	45.15	NA	Exclude IFN past year	59	CLDQ
Weng 2022	2019	obs	Database	China	Western Pacific	43.4	NA	NA	NA	122	EQ-5D-3L, EQ-5D-5L, VAS
Su 2023	2016	obs	Gastroenterology clinics of four hospitals	Taiwan	Western Pacific	60.8	NA	NA	NA	376	EQ-5D-5L, VAS
Younossi 2023	2017-2019	obs	Database	Worldwide	Various	58.2	47.7	NA	43.6% on antiviral	1818	CLDQ

Appendix 6: Summary of HRQOL in control and treatment populations

Table 6.1: Summary of HRQOL in control populations

Study	Definition of control group	HRQOL tool(s) used	Summary of findings
Chen 2021	Patients receiving annual physical examinations at the hospital.	EQ-5D-3L<br><br>VAS	EQ-5D-3L utility scores were similar between non-cirrhotic, cirrhotic and control groups<br>VAS scores were similar between non-cirrhotic and control groups
Levy 2008	Uninfected staff and students at local universities and the population at large.	SG	Non-cirrhotic patients had lower mean utilities than uninfected people
Siew 2008	Healthy randomly selected hospital staff and their friends and families, ensuring no significant illnesses	EQ-5D-3L<br><br>VAS	EQ-5D-3L utility scores were similar between non-cirrhotic and control groups<br>VAS scores were lower for those with abnormal LFTs than controls but similar between those with normal LFTs and controls
Zhuang 2014	Pair-matched healthy controls for sex, age (5 years), and occupation in the infected patient's sub-district. Excluded if any other chronic diseases or co-infections.	SF-6D	Non-cirrhotic patients had lower utility scores than controls

Table 6.2: Summary of HRQOL during treatment regimens

Study	Treatment(s) assessed	HRQOL tool(s) used	Summary of findings
Ansari 2019	Traditional medicine (qust and afsanteen)	EQ-5D-3L<br>VAS	Utility scores were increased after 12 weeks of treatment (non-cirrhotic patients)
Kim 2012	Oral antivirals (lamivudine 60%, adefovir 30%, entecavir 5.9%, clevudine 3.4%)	EQ-5D-3L<br>VAS<br>CLDQ	Utility scores were increased after 24 weeks of treatment, including in all domains of the CLDQ (non-cirrhotic, compensated cirrhosis, decompensated cirrhosis patients)
Wu 2021	Entecavir (in treatment-naïve individuals)	EQ-5D-3L<br>VAS	Utility scores increased after five years of treatment (compensated cirrhosis patients)
Xue 2017	Nucleos(t)ide analogs (in treatment-naïve individuals)	VAS	Utility scores were increased after 48 weeks of treatment (non-cirrhotic patients)
Younossi 2018	Oral antivirals (tenofovir, entecavir, adefovir, lamivudine, or telbivudine, either as a single agent or in combination)	CLDQ	Utility scores were increased at weeks 24 and 48 of treatment, especially in the Fatigue and Worry domains (non-cirrhotic patients)

Appendix 7: Quality assessment

Study design

All included articles defined the study objectives and explained the rationale for assessing HRQOL. More than half of the articles had clearly outlined study designs. However, many articles were brief in their description and may not have described the source population adequately. Fewer than half of the articles had adequate descriptions of sampling methods, with 12 articles requiring inference to conclude this. Of the 22 total articles, 7 used the consecutive sampling method which minimised selection bias, while the rest used a suboptimal strategy such as 9 articles using convenience sampling. 2 articles used purposive sampling, which may have significant selection bias associated, whilst 4 articles had unclear sampling methods. Concerning the sample size, only 1 article provided an appropriate justification for the study sample size. 2 articles did not describe participant characteristics. Most articles stated inclusion and/or exclusion criteria, however, 6 articles did not mention the exclusion of other aetiologies of liver disease, and 2 articles failed to mention any criteria. If articles included other liver disease aetiologies, this may have skewed results since these may not reflect the HRQOL utilities of HBV infection, and these patients may have worse utilities compared to patients with only HBV infection. 4 articles did not mention adequate diagnostic criteria for HBV.

HRQOL instrument and results

All articles justified the choice of HRQOL instrument(s) used, but 1 article did not mention validation of the tool in the appropriate population. Many articles did not mention the timing of the assessment and did not mention a response rate. This raises questions on the validity of the reported results as lower response rates may result in biased outcomes if patients who refused to participate were systematically different from those who participated. Of the 12

which mentioned response rates, 7 did not discuss the response rates, and 2 articles mentioned response rates were low and considered a threat to validity. No articles mentioned whether there were any missing data. It can be assumed that some articles did not have missing data and thus this was not reported, however, unreported missing data would also impact the validity of the results since as previously mentioned, if patients with missing data were systematically different from those with complete data, especially where incomplete questionnaires were discarded. All articles mentioned appropriate statistical analyses.

Interpretation

All studies stated findings clearly. However, 3 articles failed to mention any limitations, and 2 mentioned insufficient limitations when considering the total number of limitations these articles may have had.

Table 7.1: Results of quality assessment

Article	*Study Design*						
	**Study objectives**	**Study design**	**Respondent selection and recruitment**	**Sample size**	**Participant characteristics**	**Inclusion/exclusion criteria**	**Diagnostic criteria**
Ansari 2019	Yes	Yes	Yes	No	No	Partially	Yes
Che 2014	Yes	Partially	Partially	No	Yes	Partially	Yes
Chen 2021	Yes	Yes	Partially	No	Yes	Yes	Yes
Cortesi 2020	Yes	Yes	Yes	No	Yes	Yes	Yes
Dan 2008	Yes	Partially	Partially	No	Yes	Yes	No
Gupta 2020	Yes	Yes	Yes	No	Yes	Partially	Yes
Jia 2014	Yes	Partially	Partially	No	No	No	No
Kim 2012	Yes	Partially	Partially	No	Yes	No	Yes
Lam 2009	Yes	Yes	Partially	No	Yes	Yes	Yes
Lam 2009	Yes	Yes	Partially	No	Yes	Yes	Yes
Levy 2008	Yes	Yes	Yes	Yes	Yes	Yes	Yes
Liu 2016	Yes	Partially	Partially	No	Yes	Yes	Yes
Siew 2008	Yes	Yes	Yes	No	Yes	Partially	Yes
Sugimori 2022	Yes	Yes	Partially	No	Yes	Partially	No
Wong 2013	Yes	Partially	Partially	No	Yes	Yes	Yes
Woo 2012	Yes	Partially	Partially	No	Yes	Partially	No
Wu 2021	Yes	Yes	Partially	No	Yes	Yes	Yes
Xue 2017	Yes	Partially	Yes	No	Yes	Yes	Yes
Younossi 2018	Yes	Yes	Yes	No	Yes	Yes	Yes
Younossi 2019	Yes	Yes	Yes	No	Yes	Yes	Yes
Zhang 2020	Yes	Yes	Yes	No	Yes	Yes	Yes
Zhuang 2014	Yes	Yes	Yes	No	Yes	Yes	Yes

Article	*HRQOL Instrument and Results*						*Interpretation*	
	**Instrument justification**	**Instrument validation**	**Timing of assessments**	**Response rates**	**Missing data**	**Statistical analysis**	**Study findings**	**Study limitations**
Ansari 2019	Yes	Yes	Yes	Yes, but not discussed	Unclear	Yes	Yes	Partially
Che 2014	Yes	Yes	No	No	Unclear	Yes	Yes	No
Chen 2021	Yes	No	No	Yes, but not discussed	Unclear	Yes	Yes	Yes
Cortesi 2020	Yes	Yes	No	No	Unclear	Yes	Yes	No
Dan 2008	Yes	Yes	Yes	No	Unclear	Yes	Yes	Yes
Gupta 2020	Yes	Yes	No	No	Unclear	Yes	Yes	Yes
Jia 2014	Yes	Yes	Partially	Yes, threat	Unclear	Yes	Yes	Yes
Kim 2012	Yes	Yes	Yes	Yes, threat	Unclear	Yes	Yes	Yes
Lam 2009	Yes	Yes	Yes	Yes, not threat	Unclear	Yes	Yes	No
Lam 2009	Yes	Yes	Partially	Yes, not threat	Unclear	Yes	Yes	Partially
Levy 2008	Yes	Yes	No	No	Unclear	Yes	Yes	Yes
Liu 2016	Yes	Yes	No	No	Unclear	Yes	Yes	Yes
Siew 2008	Yes	Yes	Partially	Yes, but not discussed	Unclear	Yes	Yes	Yes
Sugimori 2022	Yes	Yes	Partially	Yes, but not discussed	Unclear	Yes	Yes	Yes
Wong 2013	Yes	Yes	Yes	No	Unclear	Yes	Yes	Yes
Woo 2012	Yes	Yes	No	Yes, but not discussed	Unclear	Yes	Yes	Yes
Wu 2021	Yes	Yes	Yes	No	Unclear	Yes	Yes	Yes
Xue 2017	Yes	Yes	Yes	Yes, but not discussed	Unclear	Yes	Yes	Yes
Younossi 2018	Yes	Yes	Yes	No	Unclear	Yes	Yes	Yes
Younossi 2019	Yes	Yes	Partially	No	Unclear	Yes	Yes	Yes
Zhang 2020	Yes	Yes	No	Yes, but not discussed	Unclear	Yes	Yes	Yes
Zhuang 2014	Yes	Yes	No	Yes, not threat	Unclear	Yes	Yes	Yes

Appendix 8: Subgroup analyses

Section 1: Forest plots for the non-cirrhotic stage, by utility instrument

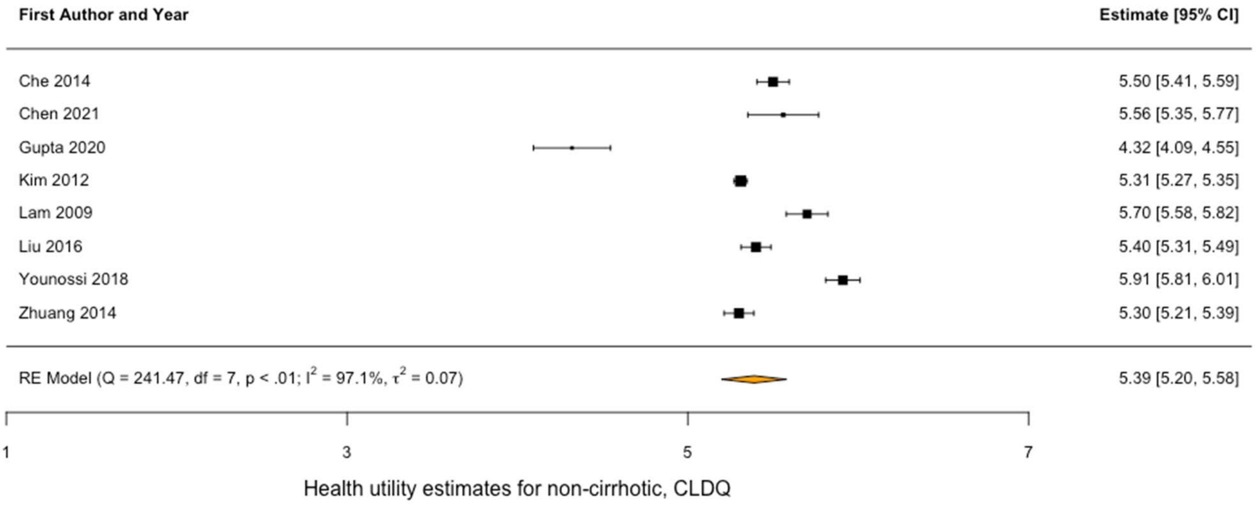

Figure 1.1: Forest plot for the non-cirrhotic stage, CLDQ instrument

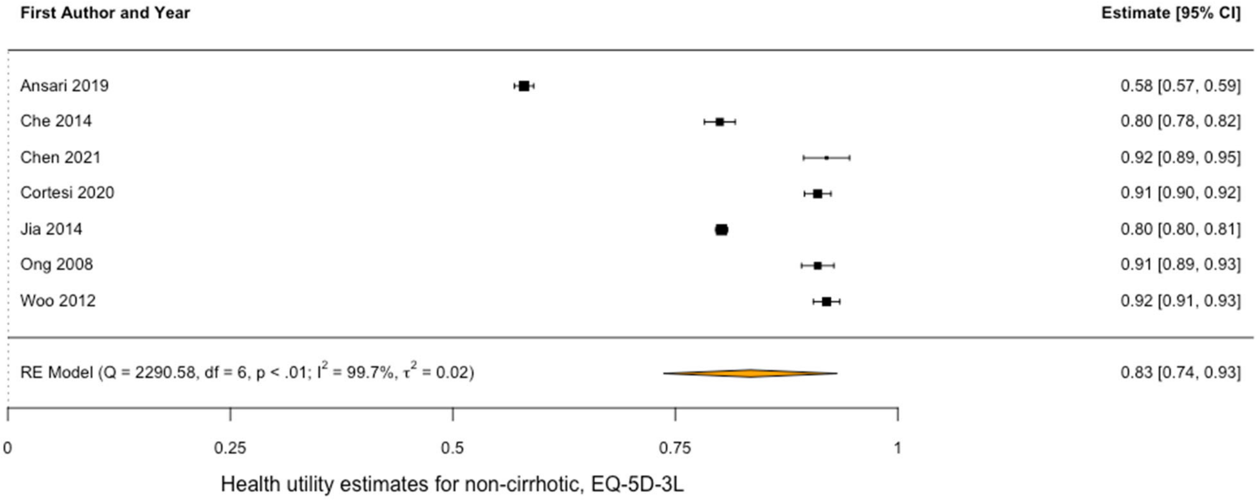

Figure 1.2: Forest plot for the non-cirrhotic stage, EQ-5D-3L instrument

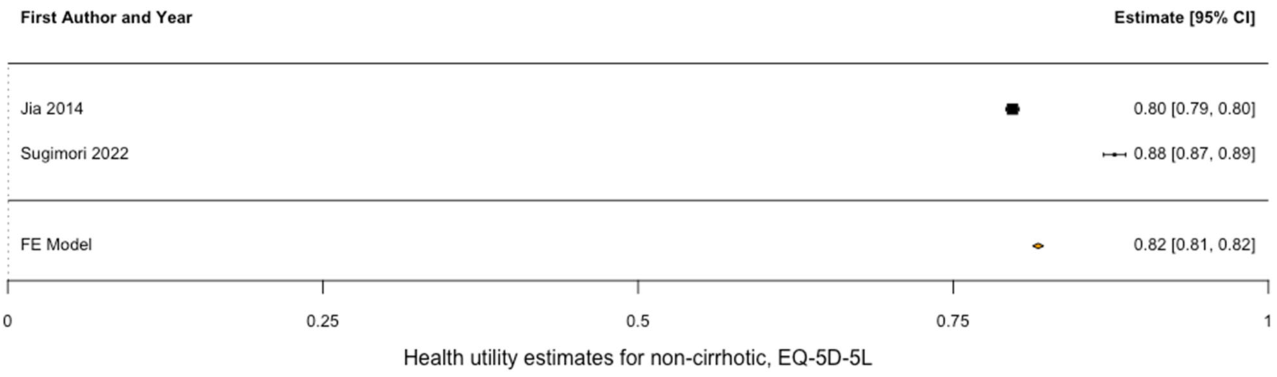

Figure 1.3: Forest plot for the non-cirrhotic stage, EQ-5D-5L instrument

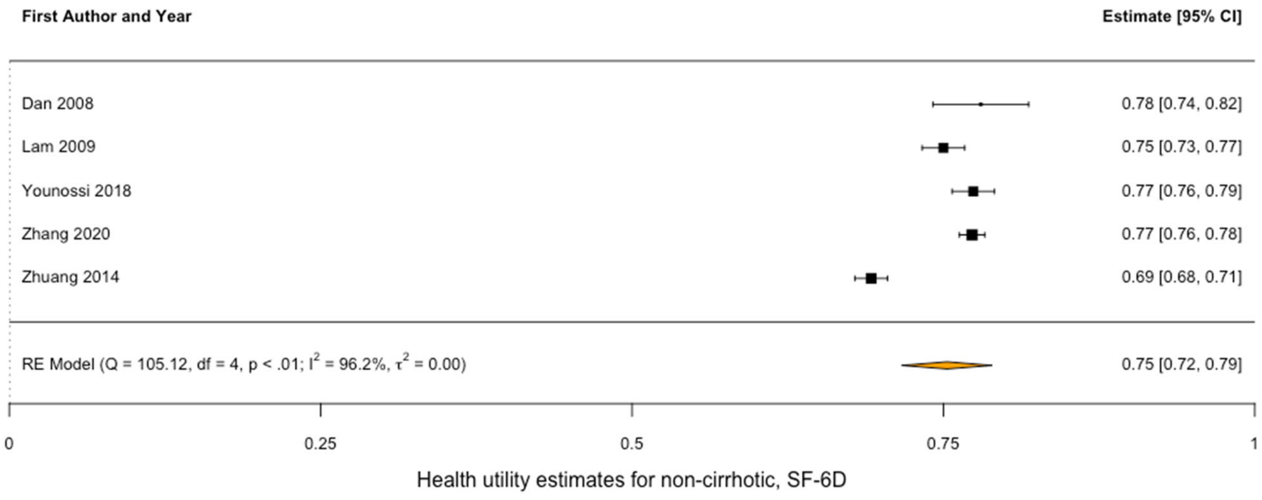

Figure 1.4: Forest plot for the non-cirrhotic stage, SF-6D instrument

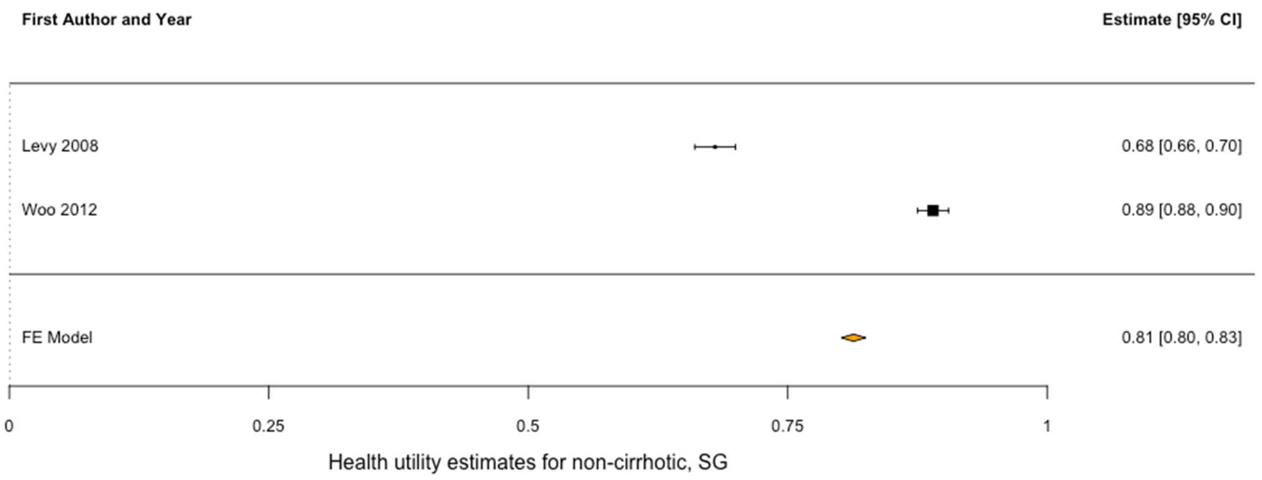

Figure 1.5: Forest plot for the non-cirrhotic stage, SG instrument

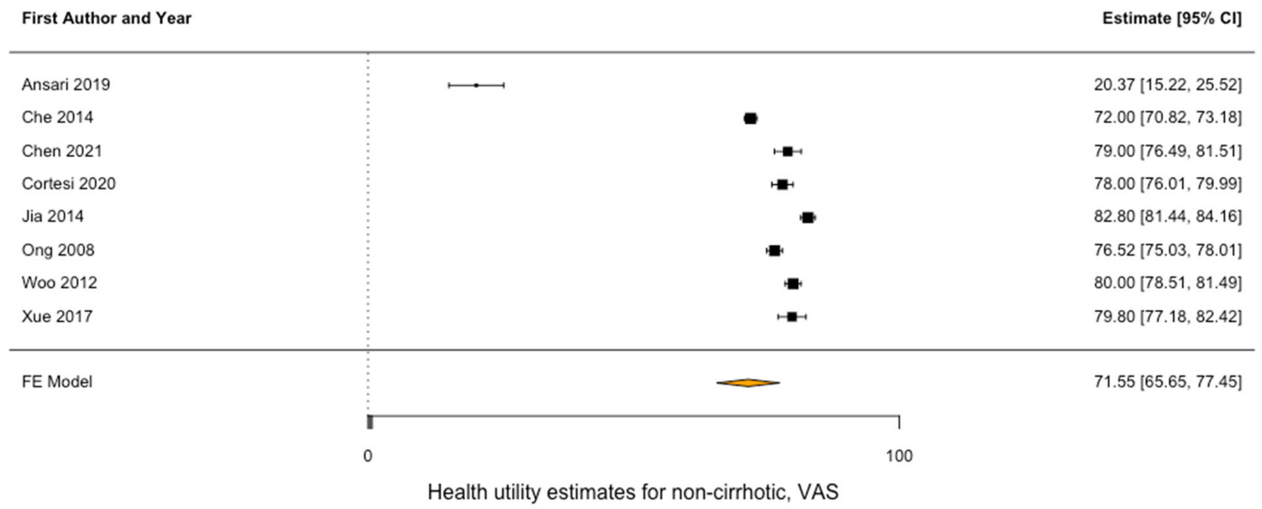

Figure 1.6: Forest plot for the non-cirrhotic stage, VAS instrument

Section 2: Forest plots for the compensated cirrhosis stage, by utility instrument

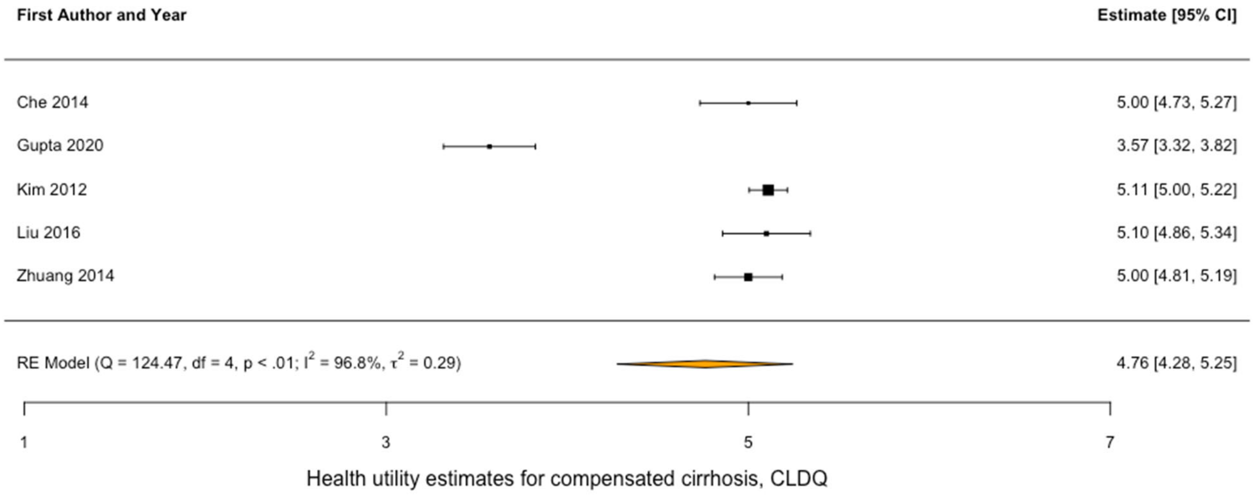

Figure 2.1: Forest plot for the compensated cirrhosis stage, CLDQ instrument

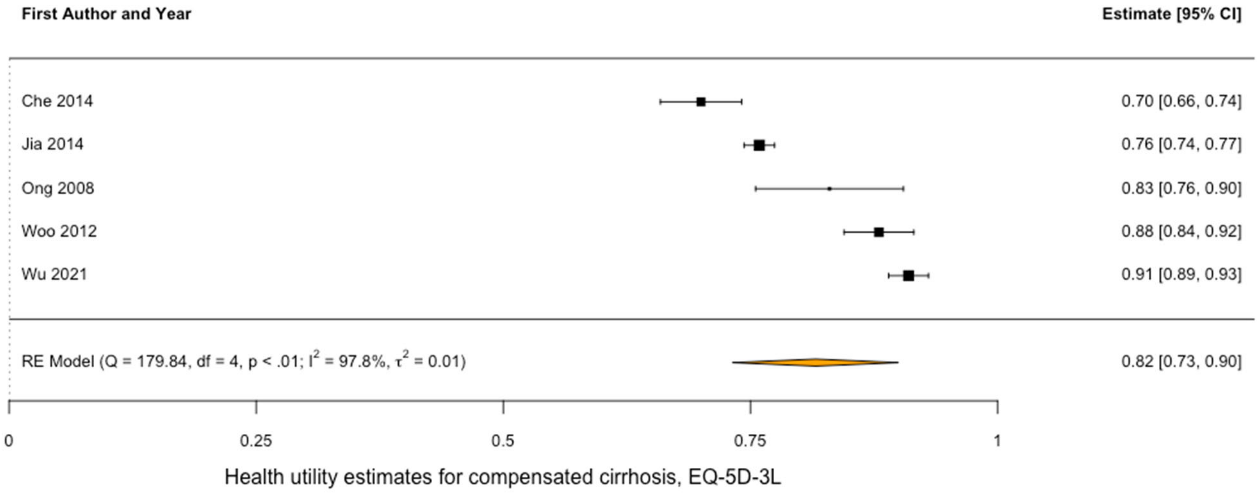

Figure 2.2: Forest plot for the compensated cirrhosis stage, EQ-5D-3L instrument

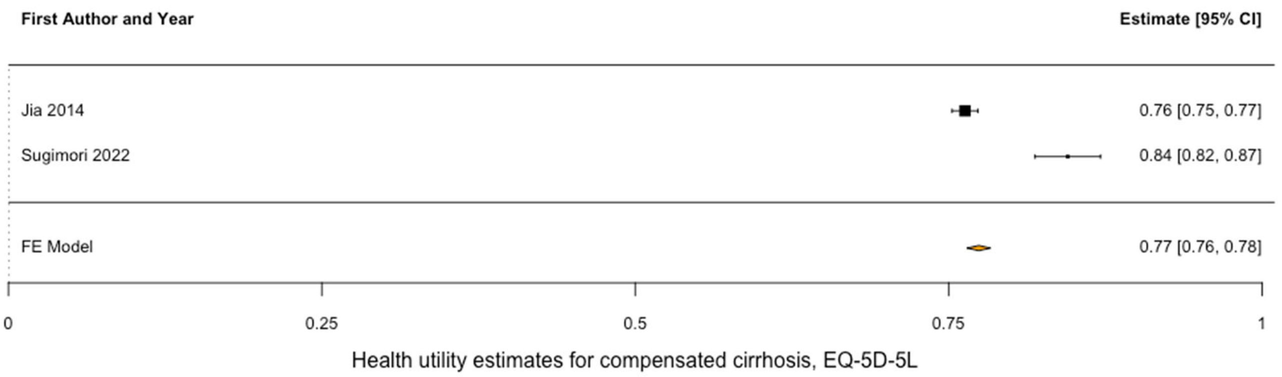

Figure 2.3: Forest plot for the compensated cirrhosis stage, EQ-5D-5L instrument

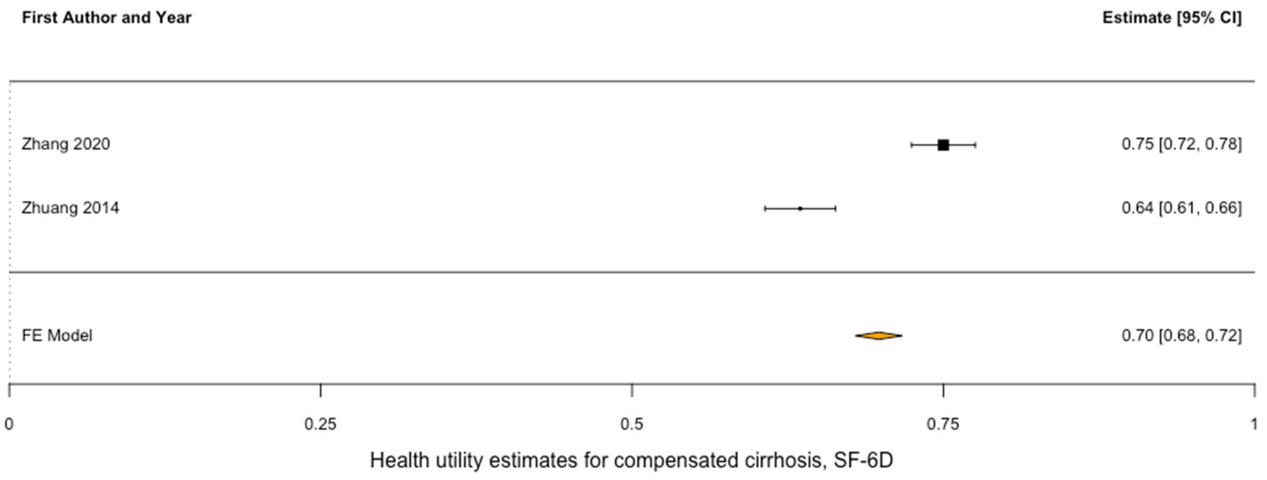

Figure 2.4: Forest plot for the compensated cirrhosis stage, SF-6D instrument

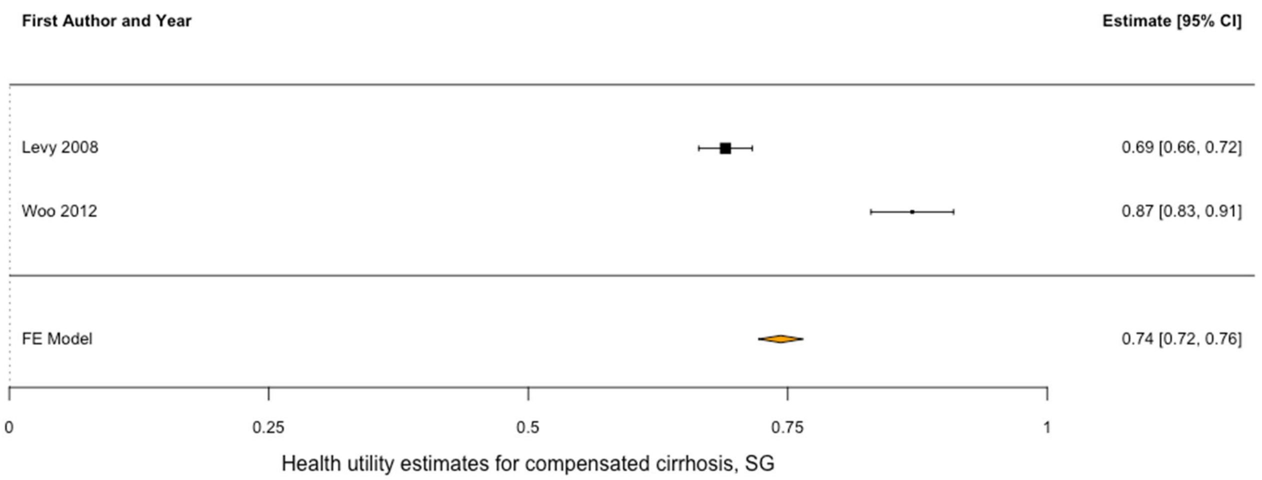

Figure 2.5: Forest plot for the compensated cirrhosis stage, SG instrument

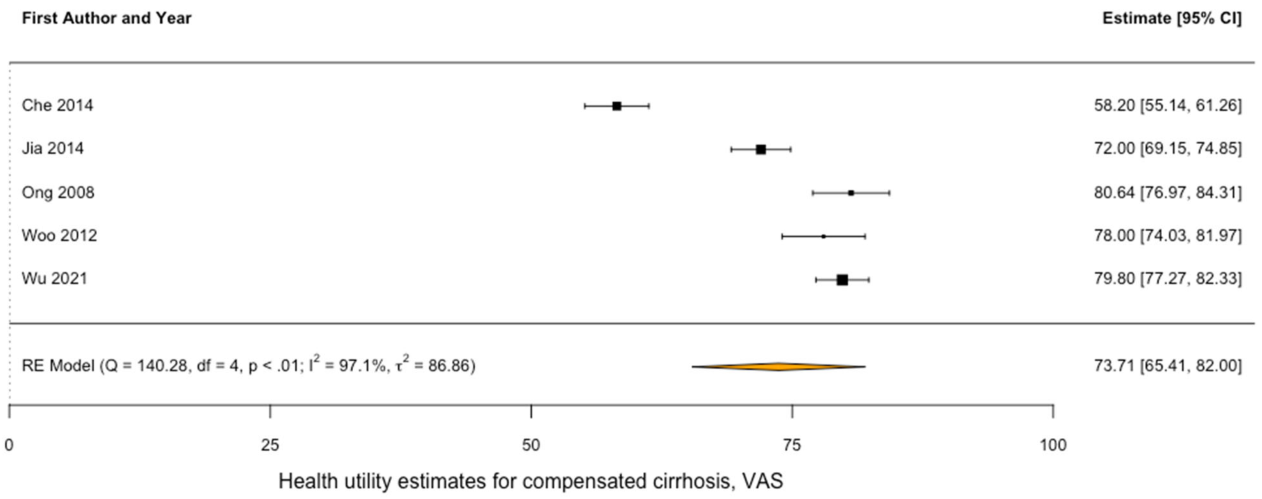

Figure 2.6: Forest plot for the compensated cirrhosis stage, VAS instrument

Section 3: Forest plots for the decompensated cirrhosis stage, by utility instrument

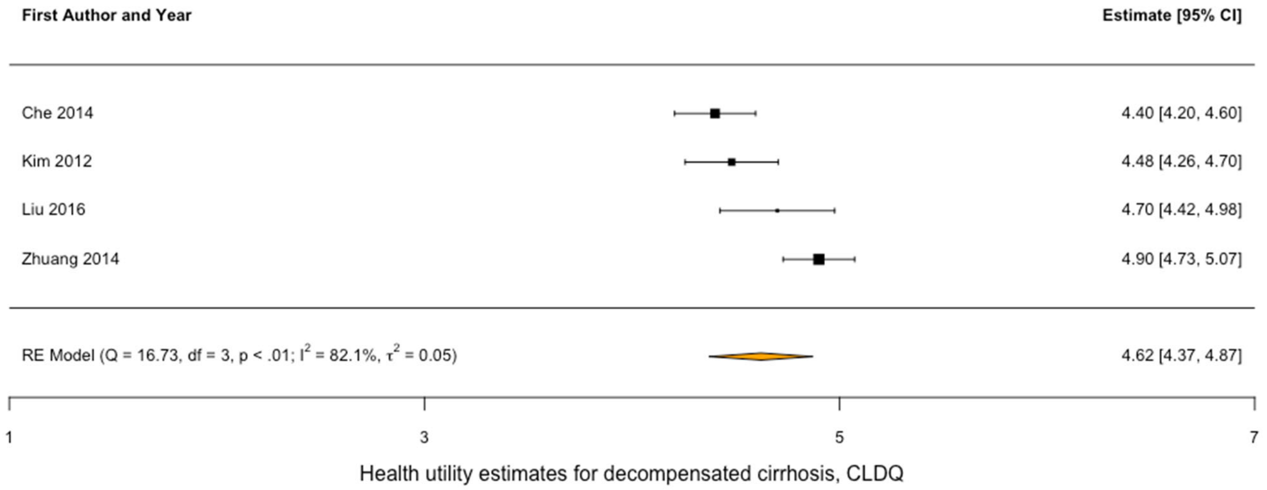

Figure 3.1: Forest plot for the decompensated cirrhosis stage, CLDQ instrument

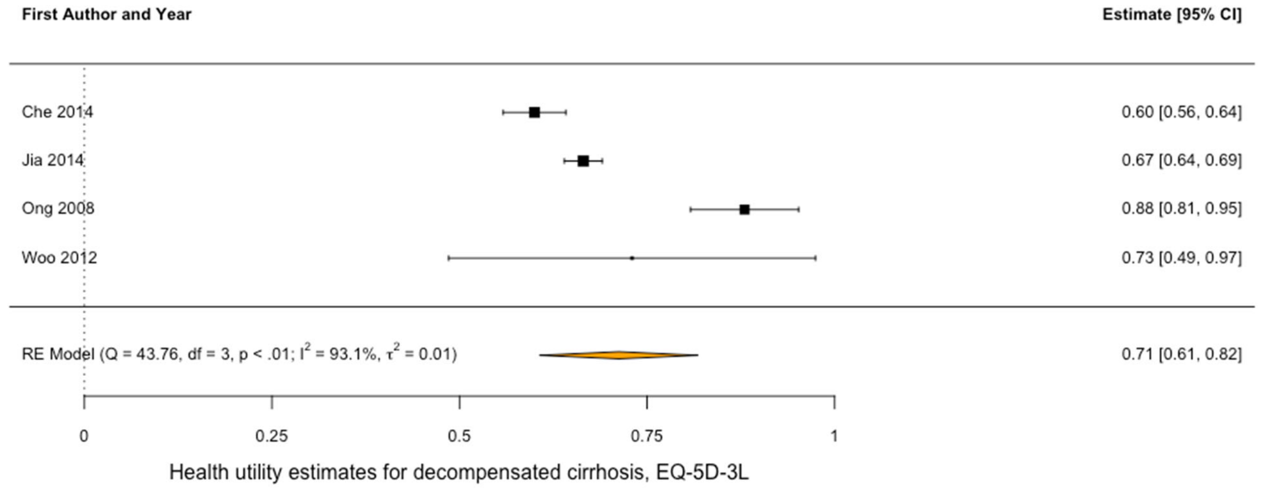

Figure 3.2: Forest plot for the decompensated cirrhosis stage, EQ-5D-3L instrument

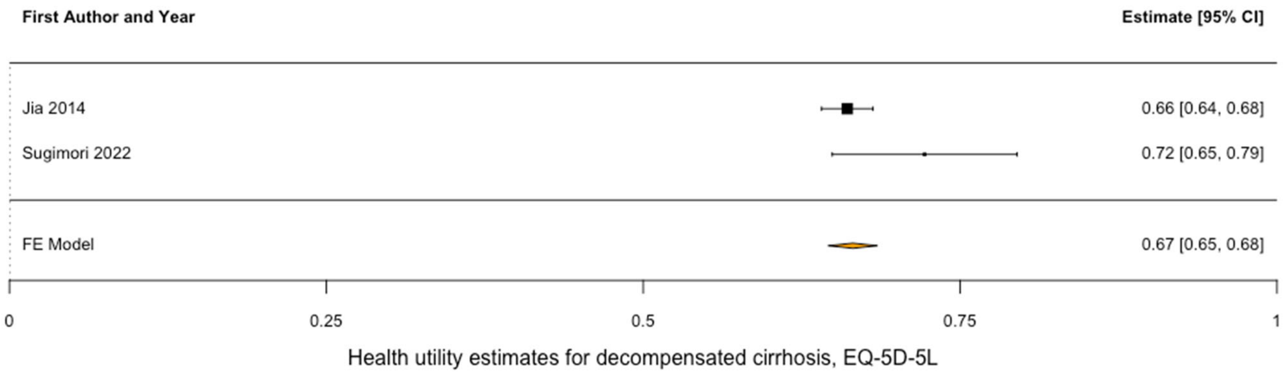

Figure 3.3: Forest plot for the decompensated cirrhosis stage, EQ-5D-5L instrument

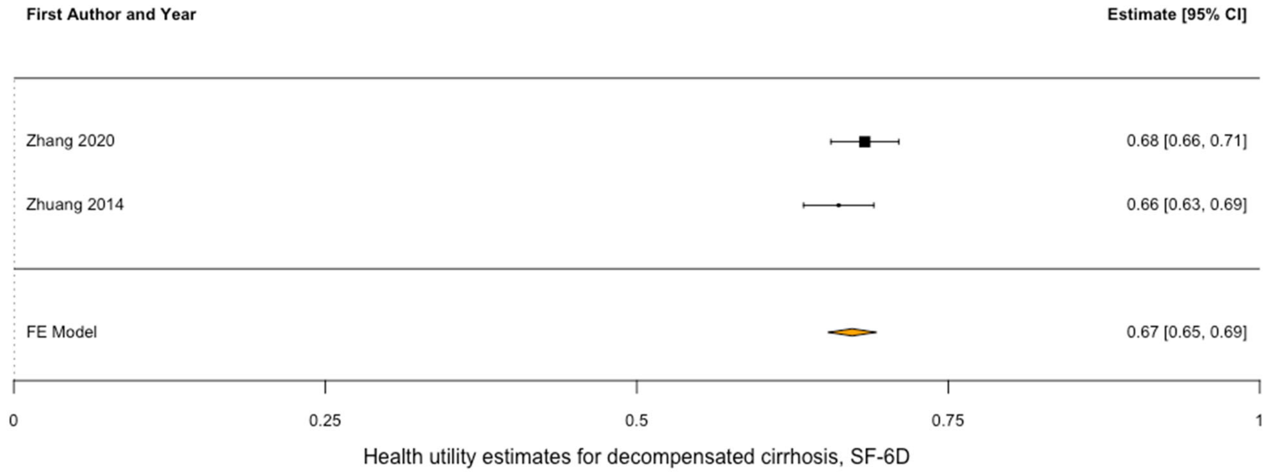

Figure 3.4: Forest plot for the decompensated cirrhosis stage, SF-6D instrument

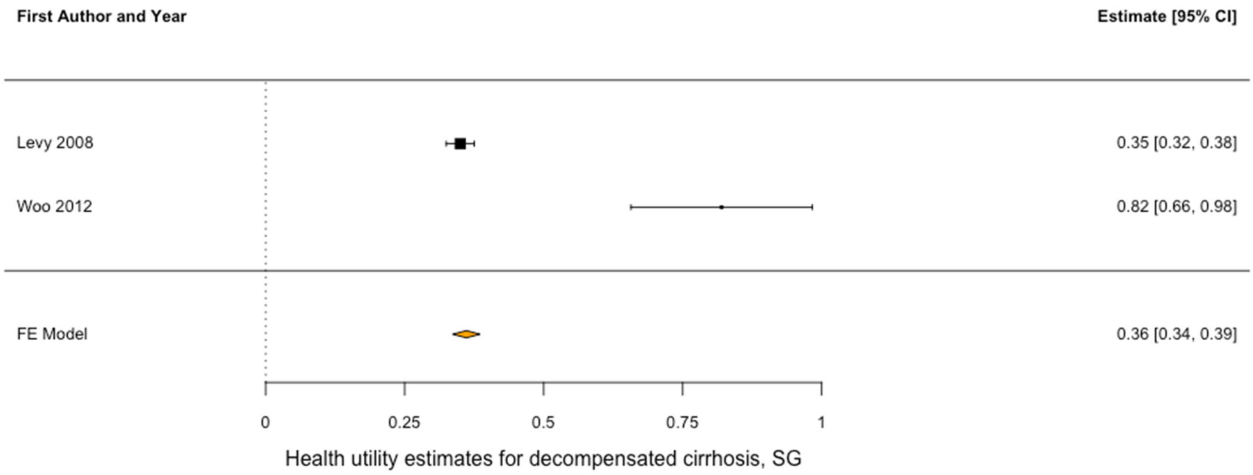

Figure 3.5: Forest plot for the decompensated cirrhosis stage, SG instrument

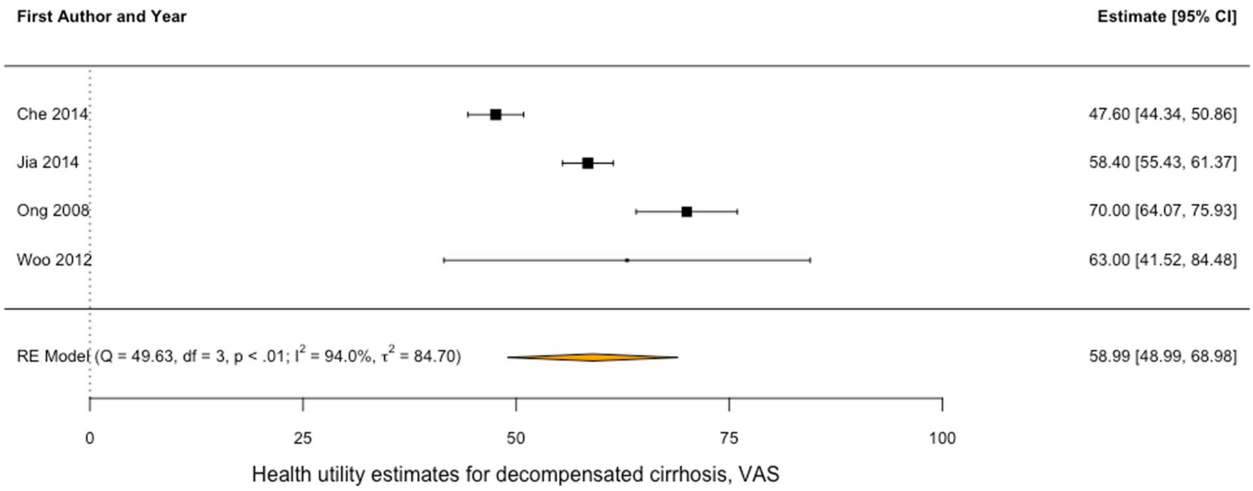

Figure 3.6: Forest plot for the decompensated cirrhosis stage, VAS instrument

Section 4: Forest plots for the hepatocellular carcinoma stage, by utility instrument

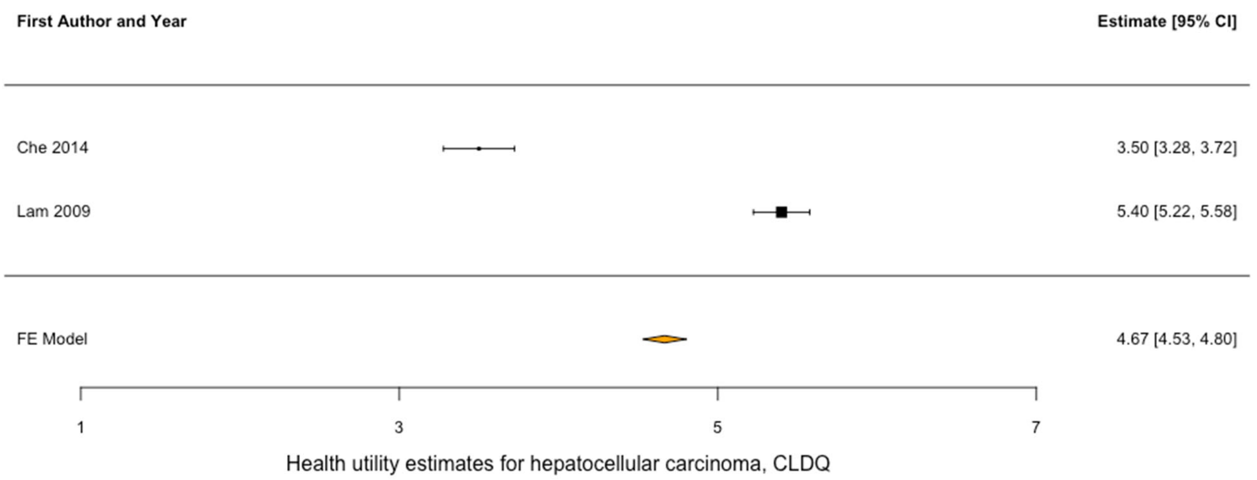

Figure 4.1: Forest plot for the hepatocellular carcinoma stage, CLDQ instrument

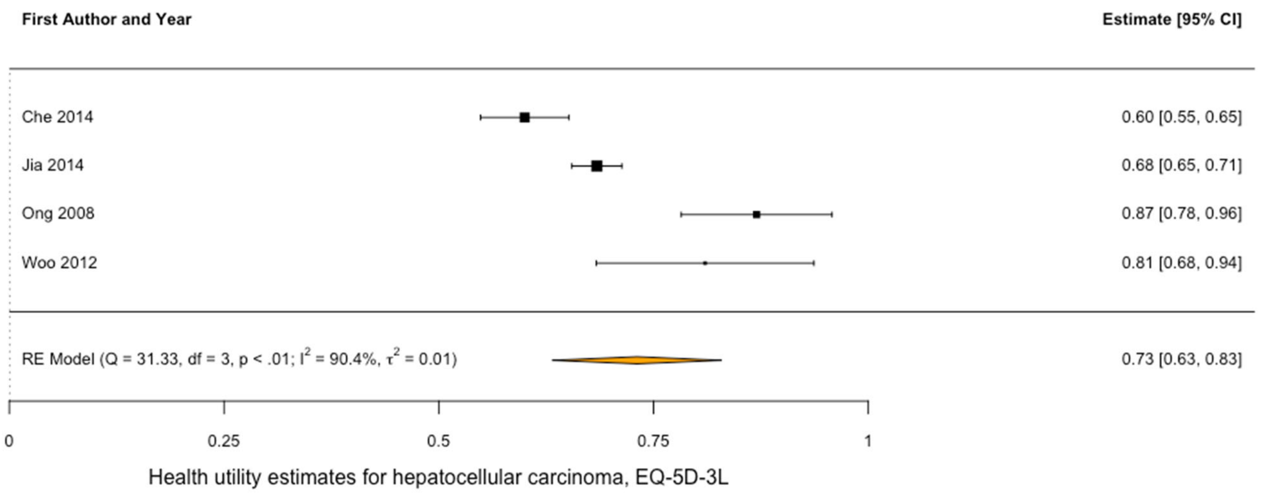

Figure 4.2: Forest plot for the hepatocellular carcinoma stage, EQ-5D-3L instrument

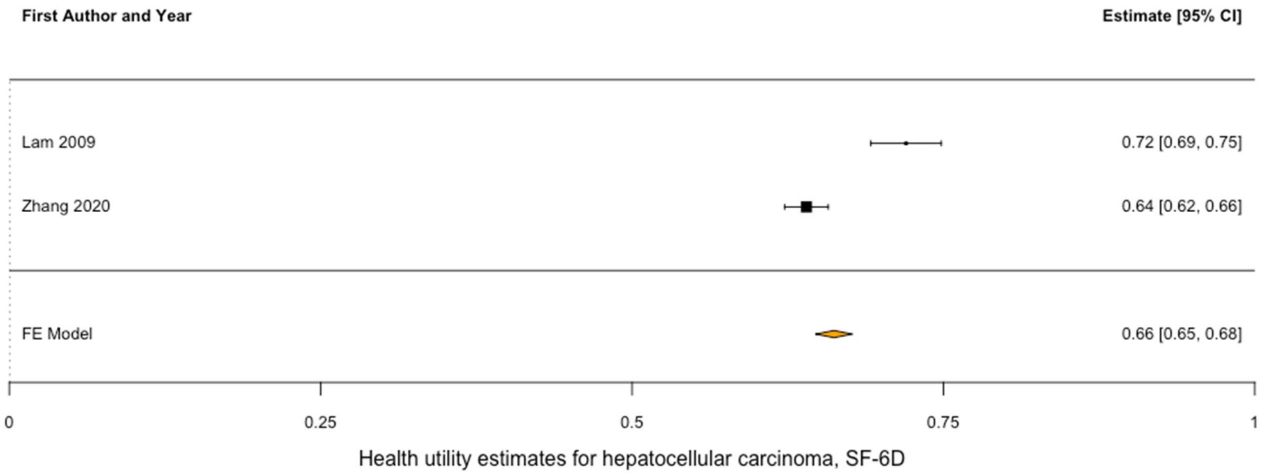

Figure 4.3: Forest plot for the hepatocellular carcinoma stage, SF-6D instrument

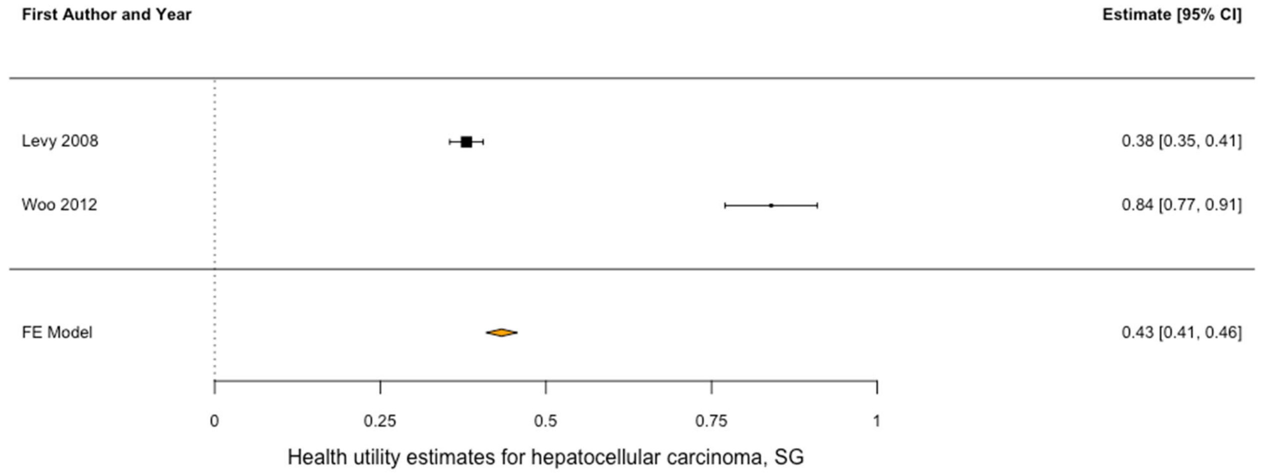

Figure 4.4: Forest plot for the hepatocellular carcinoma stage, SG instrument

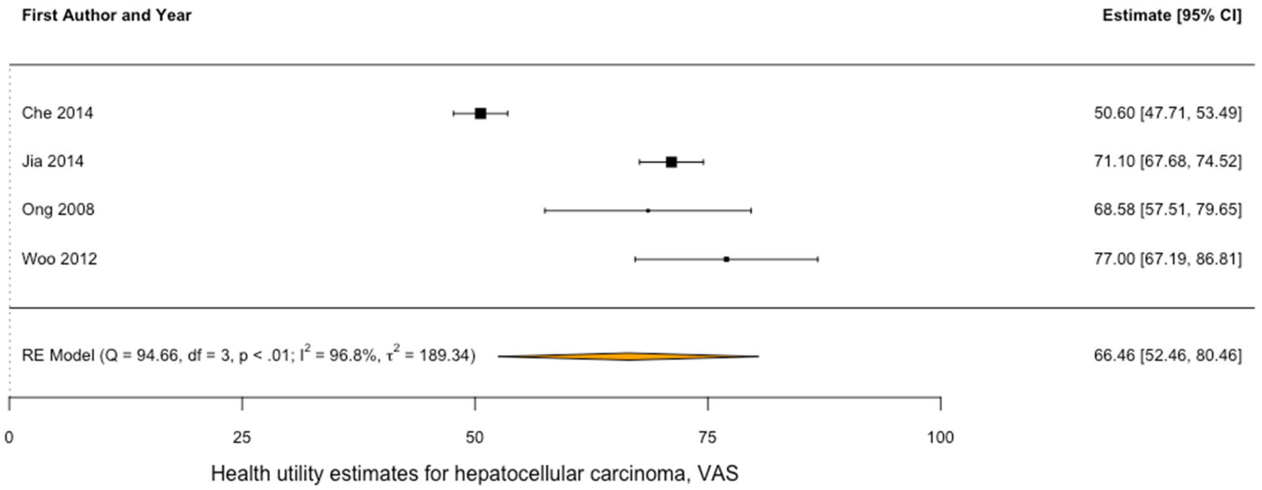

Figure 4.5: Forest plot for the hepatocellular carcinoma stage, VAS instrument

Appendix 9: Publication bias assessment, through Funnel Plots and Egger's Regression Test for Asymmetry

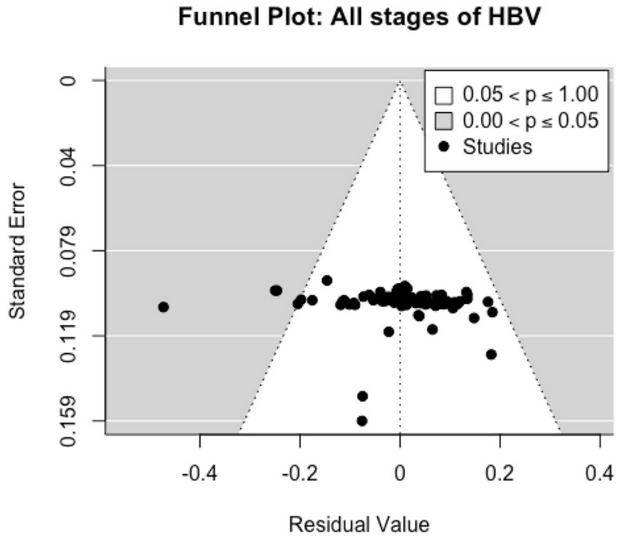

Figure 1: Funnel Plot for all stages of HBV disease

Regression test for funnel plot asymmetry: z = 0.3570, p = 0.7211. A regression test was performed using residual values and standard errors from the meta-regression model.

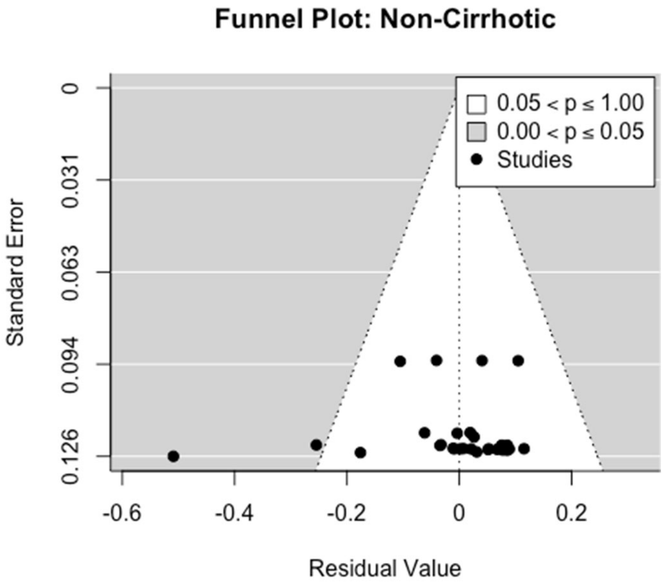

Figure 2: Funnel Plot for non-cirrhotic stage

Regression test for funnel plot asymmetry: z = -2.5974, p = 0.0094. A regression test was performed using residual values and standard errors from the meta-regression model.

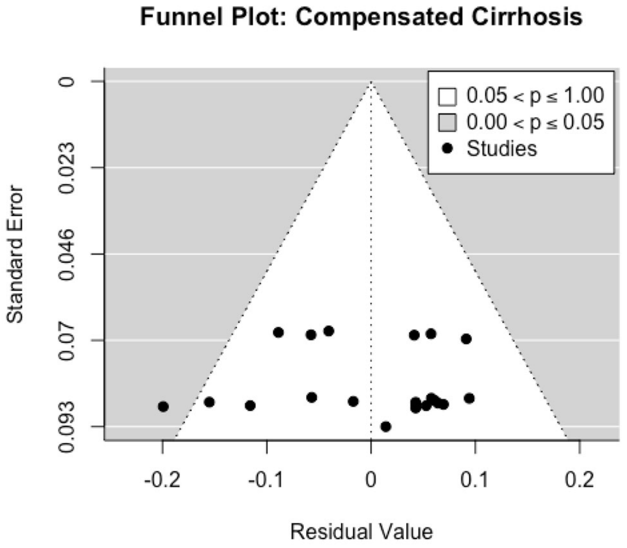

Figure 3: Funnel Plot for compensated cirrhosis stage

Regression test for funnel plot asymmetry: z = -0.0136, p = 0.9891. A regression test was performed using residual values and standard errors from the meta-regression model.

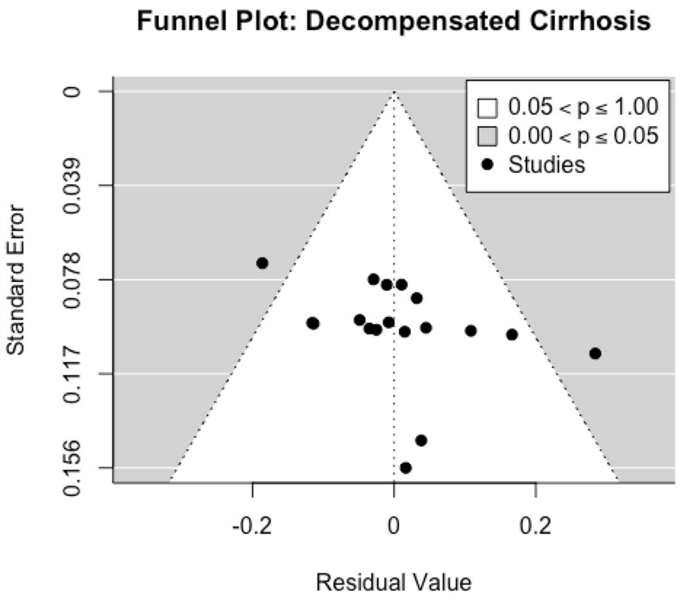

Figure 4: Funnel Plot for decompensated cirrhosis stage

Regression test for funnel plot asymmetry: $z = 2.1938$, $p = 0.0283$. A regression test was performed using residual values and standard errors from the meta-regression model.

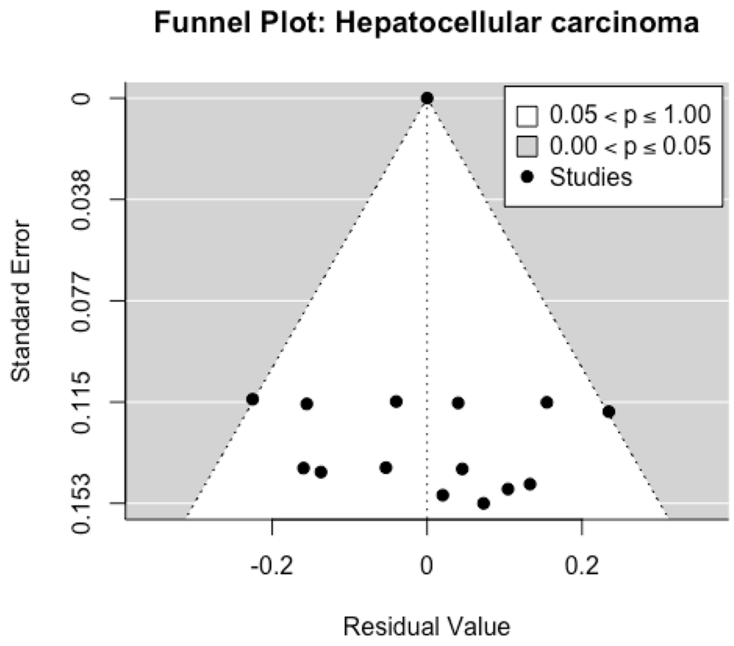

Figure 5: Funnel Plot for hepatocellular carcinoma stage

Regression test for funnel plot asymmetry: $z = 1.8272$, $p = 0.0677$. A regression test was performed using residual values and standard errors from the meta-regression model.

Supplementary references

1. Papaioannou D, Brazier J, Paisley S. NICE DSU TECHNICAL SUPPORT DOCUMENT 9: THE IDENTIFICATION, REVIEW AND SYNTHESIS OF HEALTH STATE UTILITY VALUES FROM THE LITERATURE REPORT BY THE DECISION SUPPORT UNIT. Published online 2010. Accessed April 6, 2022. www.nicedsu.org.uk

2. Critical appraisal checklist for health-related quality-of-life studies - The INTRABEAM® Photon Radiotherapy System for the adjuvant treatment of early breast cancer: a systematic review and economic evaluation - NCBI Bookshelf. Accessed April 6, 2022. https://www.ncbi.nlm.nih.gov/books/NBK311394/

3. World Health Organization. Current health expenditure (CHE) as percentage of gross domestic product (GDP) (%). 2021. Accessed January 15, 2024. https://www.who.int/data/gho/data/indicators/indicator-details/GHO/current-health-expenditure-(che)-as-percentage-of-gross-domestic-product-(gdp)-(-)

4. Levy AR, Kowdley K V., Iloeje U, et al. The impact of chronic hepatitis B on quality of life: a multinational study of utilities from infected and uninfected persons. *Value Health*. 2008;11(3):527-538. doi:10.1111/J.1524-4733.2007.00297.X

5. Younossi ZM, Stepanova M, Janssen HLA, et al. Effects of Treatment of Chronic Hepatitis B Virus Infection on Patient-Reported Outcomes. *Clin Gastroenterol Hepatol*. 2018;16(10):1641-1649.e6. doi:10.1016/J.CGH.2018.02.037
